# Examining what works for whom and how in mindfulness-based cognitive therapy (MBCT) for recurrent depression: moderated-mediation analysis in the PREVENT trial

**DOI:** 10.1192/bjp.2024.178

**Published:** 2024-11-08

**Authors:** Jesus Montero-Marin, Verena Hinze, Shannon Maloney, Anne Maj van der Velden, Rachel Hayes, Edward R. Watkins, Sarah Byford, Tim Dalgleish, Willem Kuyken

**Affiliations:** Teaching, Research & Innovation Unit, Parc Sanitari Sant Joan de Déu, Sant Boi de Llobregat, Spain; Department of Psychiatry, Warneford Hospital, University of Oxford, UK; and Consortium for Biomedical Research in Epidemiology and Public Health (CIBER Epidemiology and Public Health-CIBERESP), Madrid, Spain; Department of Psychiatry, Warneford Hospital, University of Oxford, UK; Department of Psychiatry, Warneford Hospital, University of Oxford, UK; and Department of Psychiatry, Radboudumc, Donders Institute for Brain and Behaviour, Radboud University, Nijmegen, the Netherlands; Mood Disorders Centre, School of Psychology, University of Exeter, UK; King’s College London, Centre for the Economics of Mental Health, Institute of Psychiatry, London, UK; Medical Research Council Cognition and Brain Sciences Unit, University of Cambridge, UK; Department of Psychiatry, Warneford Hospital, University of Oxford, UK

**Keywords:** Recurrent depression, mindfulness-based cognitive therapy, moderated mediation, depression severity, mindfulness skills

## Abstract

**Background:**

Personalised management of recurrent depression, considering individual patient characteristics, is crucial.

**Aims:**

This study evaluates the potentially different mediating role of mindfulness skills in managing recurrent depression using mindfulness-based cognitive therapy (MBCT) among people with varying depression severity.

**Method:**

Data from the Prevention of Depressive Relapse or Recurrence (PREVENT) trial, comparing MBCT (with antidepressant medication (ADM) tapering support, MBCT-tapering support) versus maintenance-ADM, were used. The study included pre, post, 9-, 12-, 18- and 24-month follow-ups. Adults with ≥3 previous major depressive episodes, in full/partial remission (below threshold for a current episode), on ADM, were assessed for eligibility in primary care practices in the UK. People were randomised (1:1) to MBCT-tapering support or maintenance-ADM. We used the Beck Depression Inventory-II to evaluate depressive symptom changes over the six time points. Pre-post treatment, we employed the Five Facets of Mindfulness Questionnaire to gauge mindfulness skills. Baseline symptom and history variables were used to identify individuals with varying severity profiles. We conducted Latent Profile Moderated-Mediation Growth Mixture Models.

**Results:**

A total of 424 people (mean (s.d.) age = 49.44 (12.31) years; with 325 (76.7%) self-identified as female) were included. A mediating effect of mindfulness skills, between trial arm allocation and the linear rate of depressive symptoms change over 24 months, moderated by depression severity, was observed (moderated-mediation index = −0.27, 95% CI = −0.66, −0.03). Conditional indirect effects were −0.42 (95% CI = −0.78, −0.18) for higher severity (expected mean BDI-II reduction = 10 points), and −0.15 (95% CI = −0.35, −0.02) for lower severity (expected mean BDI-II reduction = 3.5 points).

**Conclusions:**

Mindfulness skills constitute a unique mechanism driving change in MBCT (versus maintenance-ADM). Individuals with higher depression severity may benefit most from MBCT-tapering support for residual symptoms. It is unclear if these effects apply to those with a current depressive episode. Future research should investigate individuals who are not on medication. This study provides preliminary evidence for personalised management of recurrent depression.

**Trial Registration:**

ISRCTN26666654.

Depression causes significant disability to people and is a high economic burden to societies. There is a growing call to move beyond treatment to secondary prevention, focussing on the broader societal impact.^1,2^ Maintenance antidepressant medication (m-ADM) is the leading approach in preventing depressive relapse.^3^ But this does not work for everyone and can have contraindications, side-effects and some experience unpleasant withdrawal effects if they taper or discontinue use.^4^ A recent study in the UK and USA showed a 15% benefit for ADM compared with no treatment after 2 months. Only one in three people accepted antidepressants given the side-effects, and two in three expected greater treatment benefits than they experienced.^5^ Thus, there is a need for alternatives to m-ADM that support recovery without these costs.^6^ There is now compelling evidence that psychological interventions are comparably effective to m-ADM (hazard ratio = 0.86; 95% CI = 0.60, 1.23).^7^ MBCT is one such psychological intervention in which people with a history of depression learn mindfulness skills to stay well,^8^ with proven effectiveness, including as an alternative to m-ADM.^9^ However, further knowledge is needed to better tailor these interventions to individuals and maximise their effectiveness.

Translational science enhances our understanding of who benefits most from which treatment, and through which mechanisms different treatments operate. This enables us to innovate the treatments themselves by better targeting the mechanisms and also ensuring that treatments most likely to be effective are offered to people most likely to benefit. A recent scoping review suggests improvements in mindfulness skills (which include present-moment attention and non-judgemental acceptance) as a potential target mechanism of mindfulness-based programmes across different mental health strategies (e.g. treatment and prevention).^10^ This supports theoretical assumptions on how these programmes work by helping people to break established cognitive patterns that maintain and enhance depressive symptoms. However, more studies are required to determine whether this potential mechanism is shared across, or is unique to, different subgroup populations.^10^ The recent scoping review mentioned above also offers additional conceptual guidelines for analysing mediation, recommending the use of high-quality randomised controlled trials (RCTs), active comparators targeting different mechanisms and innovative statistical approaches within the context of an embedded process evaluation framework.^11^

## Objective

The present study explores potential moderated-mediation effects in the management of recurrent depression following treatment with m-ADM compared with MBCT. A moderated-mediation analysis offers an opportunity to better understand for whom and why MBCT (versus m-ADM) may have different effects on people with depression. Building on previous research suggesting that MBCT may be especially helpful in the context of entrenched depression, we hypothesised that the putative mediating effects of improvements in mindfulness skills are specific to MBCT and are stronger in the subgroup of individuals with a more severe history of depression.^12^

## Method

This study is a post-hoc extension on planned secondary analysis of the Prevention of Depressive Relapse or Recurrence (PREVENT) trial.^13^ PREVENT was a single-blind parallel RCT, examining m-ADM versus MBCT (with ADM tapering support, MBCT-tapering support) for people with recurrent depression. Results of the PREVENT trial showed that both treatments were associated with enduring positive outcomes. To better tailor treatments to individuals and maximise their effectiveness, the PREVENT trial was envisioned to explore process-outcome relationships of mindfulness skills beyond the overall effectiveness and cost-effectiveness findings.^13,14^

### Participants

The sample consisted of adult participants aged 18 years or older, with a diagnosis of recurrent depression, who were taking m-ADM. Participants were not currently experiencing a depressive episode at the time of the study. They were recruited from various locations within the south-west region of England (Bristol, north Devon, east Devon and mid Devon), totalling 424 participants. The sample size was not established a priori for moderated-mediation analyses, thus the secondary analyses reported in the current paper were exploratory.^14^ Nevertheless, the exploration of mindfulness skills as a potential mechanism of MBCT to be tested was already established in the protocol,^14^ and we carried out sensitivity analysis to control for potential confounding effects. Participants gave informed consent to participate in the study before taking part (available upon request).

### Randomisation and masking

The 424 participants were randomly assigned (1:1) to m-ADM or MBCT-tapering support and stratified by recruitment site and symptomatic status (asymptomatic or partially symptomatic) via computer-generated random permuted blocks, using a password-protected website externally hosted by the Peninsula Clinical Trial Units. Researchers working on the PREVENT were blind to treatment allocation. However, given the nature of the interventions, participants were aware of their treatment group assignment.

### Procedures

#### Recruitment

Participants were recruited and received treatment in primary care settings. The inclusion/exclusion criteria were refined during a feasibility trial,^15^ to maximise real-world applicability for the primary care patient population who experienced recurrent depression and were treated with m-ADM and were interested in exploring a psychological approach. Inclusion criteria for study participation required individuals to have a diagnosis of recurrent Major Depressive Disorder (MDD) in full or partial remission as per DSM-IV,^16^ with a history of three or more previous MDD episodes, to be 18 years or older, and to be on a therapeutic dose of m-ADM, following the British National Formulary (BNF) and the National Institute for Health and Care Excellence (NICE) guidelines.^17^ Exclusion criteria included a current depressive episode, concurrent substance misuse, organic brain damage, current or past psychosis (including bipolar disorder), antisocial behaviour, ongoing self-injury requiring clinical management and/or therapy, and receiving concurrent psychotherapy. Participants provided written informed consent after receiving a description of the study.

#### Interventions

MBCT is a manualised group programme aimed at teaching skills to prevent depression relapse. The goal of MBCT is to increase people’s awareness of bodily sensations, thoughts and feelings linked to depressive relapse and to help them respond to these experiences in a constructive manner. Participants practice mindfulness exercises during sessions and through homework, with therapists supporting them in developing adaptive responses to potential triggers of depression. The original MBCT programme was adapted, placing emphasis on developing a relapse and recurrence signature and response plan, which involved participants considering the reduction or discontinuation of m-ADM (Supplementary Material S1 available at https://doi.org/10.1192/bjp.2024.178).^8^ The MBCT-tapering support programme consisted of eight 2.25-hour group sessions, typically held over consecutive weeks, with up to four booster sessions offered in the year following the end of the 8-week programme. Participants in the MBCT-tapering support arm were encouraged to taper and discontinue their m-ADM towards the end of the 8-week programme. The research team provided information for general practitioners (GPs) and participants, regarding typical tapering and discontinuation regimens and potential withdrawal effects. A total of 21 MBCT-TS groups (around ten individuals per group) were led by four experienced MBCT teachers. Teachers were mental health professionals (two clinical psychologists and two occupational therapists), with extensive training and experience in leading MBCT groups (≥4 years) and a long-standing ongoing personal mindfulness practice (≥7 years). An independent check on competency was established before teachers progressed to running trial groups. For that, an experienced MBCT therapist independent of the trial rated at least two videotapes of MBCT-tapering support sessions and, using the Mindfulness-Based Interventions–Teacher Assessment Criteria (MBI-TAC), made an overall judgement about whether the teachers were competent. During the trial, MBCT teachers received 3-hour supervision biweekly. Trial groups were videotaped for checks on therapist competence and adherence. Randomly selected samples of two sessions (42 sessions in total) were assessed by a MBCT expert independent of the trial. Transcription coding of the MBCT-tapering support trial sessions indicated that the teachers delivered the groups at or above the required levels of competence (Supplementary Material S2). The mean (s.d.) total adherence score in the trial was 23.6 (4.30) – potential range 0–34 – indicating acceptable adherence to protocol, with no differences found between teachers.^13^

The m-ADM arm consisted of maintenance of the ADM treatment. Participants were monitored and treated by GPs in a primary care setting in line with standard clinical practice. Primary care physicians were asked to meet with patients regularly to review their medication. Changes in medication sometimes occurred, but physicians and participants were asked to ensure that the dose remained within therapeutic limits. The trial GPs and trial psychiatrist provided materials for all participants and participating GPs on m-ADM and ongoing support as required.

#### Ethics

The authors assert that all procedures contributing to this work comply with the ethical standards of the relevant national and institutional committees on human experimentation and with the Helsinki Declaration of 1975, as revised in 2013. All procedures involving human patients in the PREVENT trial were approved by the UK National Health Service South-West Research Ethics Committee (09/H0206/43), obtained research governance approval from local primary care trusts or health boards, and were overseen by a data monitoring and ethics committee and the PREVENT trial steering committee.

#### Measures

Supplementary Material S3 provides details on the measures and their corresponding references. The following sociodemographic information was collected prior to trial randomisation at baseline: age, self-identified gender, self-identified ethnicity, level of education, relationship status and employment status.

Informed by previous theoretical and empirical research describing factors that predict whether MBCT could offer superior relapse prevention for recurrent depression compared with m-ADM,^12,18–23^ we used a series of baseline-measured variables to define different latent profiles characterised by distinct severity of clinical history. For that, we considered (a) markers of symptoms intensity and clinical history, (b) cognitive and emotional factors, and (c) relational and social variables (all assessed prior to randomisation). Symptoms intensity and clinical history variables included clinician-rated residual symptoms of depression (Hamilton Depression Rating Scale (HAMD)); childhood abuse (Measure of Parenting Scale (MOPS)); age of first onset of depression (years); number of previous depressive episodes; severity of last episode (number of symptoms present from the Structured Clinical Interview for DSM-IV (SCID); potential range: 5–9); chronicity of last episode (months); previous suicide attempt (yes, no); and number of comorbid DSM-IV axis I diagnoses.^16^ Cognitive and emotional factors involved cognitive rumination (negative and un-resolution rumination, from the Cambridge-Exeter Repetitive Thought Scale (CERTS)); self-blame and lack of acceptance (from the Cognitive Emotion Regulation Questionnaire (CERQ)); ability to recognise early warning signs of depression (bespoke single item); acting with awareness (from the Five Facet Mindfulness Questionnaire (FFMQ)); self-efficacy (General Self-Efficacy Scale (GSE)); and positive affect (contentment and joy, from the Dispositional Positive Emotion Scale (DPES)). Relational and social factors covered relationship satisfaction (bespoke 7-items) and stigmatisation (bespoke 7-items). We assessed quality of life at baseline using the World Health Organization Quality of Life questionnaire (WHOQOL-BREF) as a distal measure (not included in the analytical processes of identifying/confirming the latent profiles) to assist in understanding the observed latent profiles.

We focused on mindfulness skills as a potential MBCT-tapering support mediator because they have been widely supported empirically, despite variable effects. Variations suggest that the influence of mindfulness skills might depend on participant characteristics, warranting further investigation.^10^ Mindfulness skills were measured at baseline and 1 month after the MBCT-tapering support training (or the equivalent in the m-ADM arm) using the FFMQ total score to examine improvements in mindfulness skills as a hypothesised mechanism.

We selected the total score of the Beck Depression Inventory (BDI-II), taken as a continuous variable, as our primary measure to monitor changes in the intensity of depressive symptomatology over time (from baseline to 1 month after the end of the MBCT-tapering support training, and at 9, 12, 18 and 24 months after baseline). The BDI-II is one of the most widely used instruments for assessing the presence and intensity of depressive symptoms. It is a self-reported measure composed of 21 items covering cognitive, emotional and somatic domains associated with depression. It aligns with diagnoses from the DSM-IV and has demonstrated strong concordance with clinical diagnoses of depression. The BDI-II inquires about the preceding 2 weeks and requires participants to rate symptom levels ranging from 0 (‘not present’) to 3 (‘severe’), with a total score ranging from 0 to 63 (0–13: minimal depression; 14–19: mild depression; 20–28: moderate depression; 29–63: severe depression). It demonstrates high convergent validity with other depression rating scales, such as the Hamilton Depression Rating Scale, and exhibits robust psychometric properties, including strong internal consistency and test-retest reliability. Its comprehensive coverage ensures a nuanced evaluation of depressive symptoms, making it an invaluable tool for monitoring shifts in symptom severity throughout the course of treatment. A clinically relevant improvement generally depends on the initial levels of depression and is usually established at around a 15% improvement.^24^ However, NICE guidance suggests that a change of three or more BDI-II points is clinically significant. This BDI-II change has, in fact, been demonstrated to be relevant for service use patients with average BDI-II scores of around 14 points.^24^

### Statistical analyses

A moderated-mediation growth mixture model was used to evaluate the indirect effects at varying levels of the moderator (i.e. the latent profiles). Moderated-mediation analyses examine the conditional indirect effect of a potential moderating variable on the relationship between a predictor variable and an outcome, through a potential mediator. The predictor was the allocation group (m-ADM versus MBCT-tapering support). The outcome was the linear growth (β_1_) of the latent change trajectory of depressive symptoms (BDI-II) across all trial time points (from baseline to 24 months later), as a measure of the rate of change of depressive symptoms over time.^25^ The potential mediator was the change in mindfulness skills (ΔFFMQ) from baseline to 1 month after the end of the MBCT training. The potential moderator was the latent profile variable, reflecting distinct subgroups of individuals with recurrent depression in remission, characterised by a distinct degree of clinical severity. Details of the latent profile and latent growth curve model (LGCM) analyses are provided in Supplementary Material S4.

We tested the potential moderating effect of the latent profile on the predictor-to-mediator path (*a*-path), on the mediator-to-outcome path (*b*-path) and on the predictor-to-outcome direct path after controlling for the indirect effect (*c*′-path). We used the index of moderated-mediation (i.e. the difference in the indirect effects across levels of the potential moderator) to test the significance of the moderated-mediation.^26^ Significant effects are supported by the absence of zero within the bootstrapped 95% CIs.^27^ Sensitivity analyses were conducted whereby the moderated-mediation model was adjusted to account for the potential confounding effects of the amount of home-based formal meditation practice (‘not at all’, ‘sometimes’, ‘regularly’, ‘more days than not’) and discontinuation of antidepressant intake (i.e. whether they remained on a therapeutic dose of antidepressants for the duration of the trial). A graphical representation and more details of the moderated-mediation analyses are provided in Supplementary Material S5.

We examined missing data and applied Full Information Maximum Likelihood assuming missingness at random.^28,29^ We used 2-tailed tests with an alpha level of 0.05 and 95% bootstrapped CIs for the indirect effects. Because of the exploratory nature of this study, we opted not to correct for multiple testing. Analyses were performed using Mplus v8.10 for MacOS.

## Results

Descriptive statistics for baseline sociodemographic, quality-of-life and depressive symptoms variables are presented by trial arm in Table 1. In the MBCT-tapering support arm, 176 participants (83.0%) completed four or more MBCT-tapering support sessions. Additionally, 140 participants (66.1%) engaged in home-based formal meditation practices (‘sometimes’, ‘regularly’ or ‘more days than not’), and 133 participants (62.7%) did not remain on a therapeutic dose of antidepressant medication for the duration of the trial (m-ADM use in people who attended ≥4 sessions of MBCT-tapering support can be seen elsewhere).^13^ In the m-ADM arm, 23 participants (10.8%) practised home-based formal meditation exercises, and 50 participants (23.6%) did not remain on a therapeutic dose of antidepressant medication for the duration of the trial (Supplementary Material S6). Details on antidepressant use according to participants’ self-reports, and the number of visits registered in the GP record by group can be seen in Supplementaries S7 and S8. Of the participants recruited to the trial, 348 (82.1%) provided data on the BDI-II at one month after the end of the MBCT-tapering support training; 293 (69.1%) provided data at 9 months after baseline; 324 (76.4%) at 12 months; 291 (68.6%) at 18 months; and 336 (79.3%) at 24 months. Participant baseline characteristics by trial arm and follow-up status are presented in Supplementaries S9–S13.

### Latent profiles

**Fig. 1:**
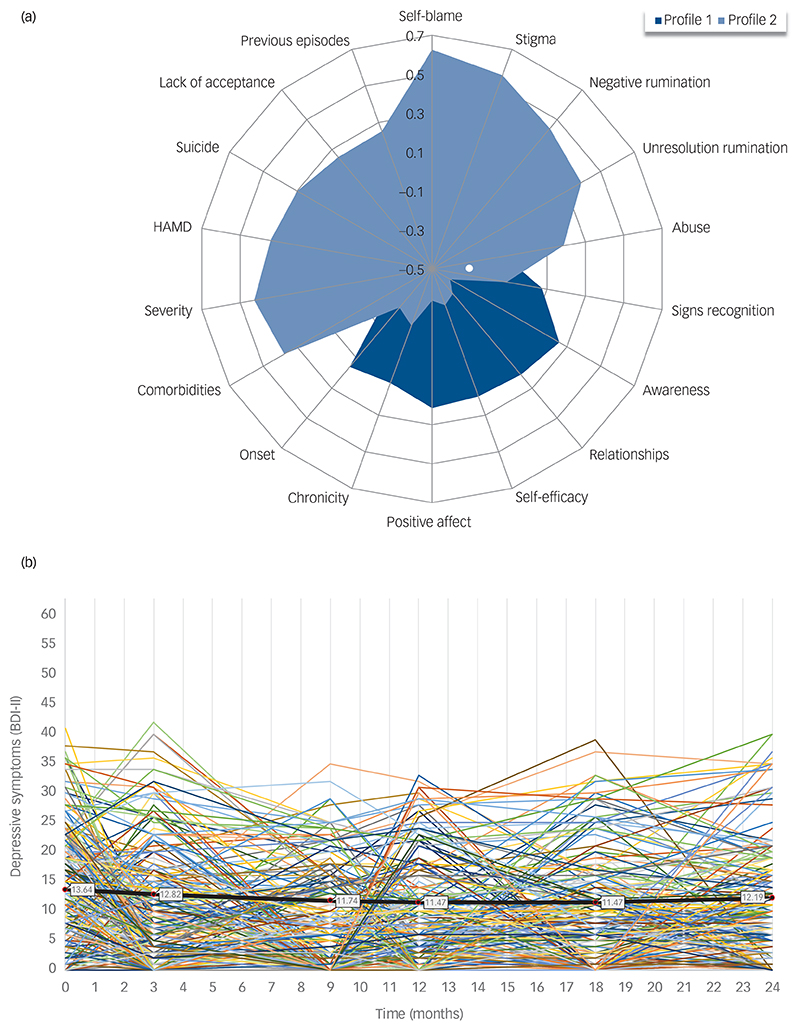
Latent profiles and individual trajectories. (a) Latent profiles graphical representation. Profile 1: latent profile 1 (i.e. less severe history of depression). Profile 2: latent profile 2 (i.e. more severe history of depression). Total sample *N* = 424. Because of the different scaling of the continuous and dichotomous items included in the latent profile analysis, all mean scores and proportions for each predictor were standardised, and z-scores were used to present the distribution between mean scores and proportions for each profile. We used a range of potential predictor variables to define latent profiles demarcated by a different depression severity. These variables fall into three categories: (1) symptoms intensity and clinical history (clinician-rated residual symptoms of depression (Hamilton Depression Rating Scale (HAMD)), childhood abuse (Measure of Parenting Scale (MOPS)), age of first depression onset, number of previous episodes of depression, severity of the last episode (Structured Clinical Interview for DSM-IV (SCID)), chronicity of the last episode, previous suicide attempt, number of comorbid DSM-IV axis I psychiatric diagnoses (SCID)); (2) cognitive and emotional factors (rumination (negative and un-resolution rumination from the Cambridge-Exeter Repetitive Thought Scale (CERTS)), self-blame and lack of acceptance (from the Cognitive Emotion Regulation Questionnaire (CERQ)), ability to recognise early warning signs of depression (bespoke single item), acting with awareness (from the Five Facet Mindfulness Questionnaire (FFMQ)), self-efficacy (General Self-Efficacy Scale (GSE)), positive affect (contentment and joy from the Dispositional Positive Emotion Scale (DPES))); (3) relational and social variables (relationship satisfaction (bespoke 7-item questionnaire), stigmatisation (bespoke 7-item questionnaire)). These variables were measured at baseline (T0) and were selected based on theoretical and empirical research.^20^ (b) Individual trajectories and estimated means of depressive symptoms over time. The coloured lines represent the observed individual trajectories, with this representation including a subset of *n* = 250 randomly selected participants. The thick black line represents the mean estimated by the quadratic latent curve growth model for the total group (*N* = 424). BDI-II is the Beck Depression Inventory-II (range: 0–63). Participants were assessed on the BDI-II at baseline (0 months), and then post-treatment (3 months, i.e. one month after the end of the mindfulness-based cognitive therapy with support to taper or discontinue antidepressant medication (ADM) training, or the equivalent time in the maintenance ADM arm). Follow-up measures include 9, 12, 18 and 24 months after randomisation.

Supplementary Material S14 presents the model selection, latent profile interpretation and patient classification according to the latent profile analysis. A two-level model (latent profile 1: ‘lower severity of depression’; latent profile 2: ‘higher severity of depression’) was estimated. Figure 1(a) includes a graphical representation of the distribution of predictor variables between latent profiles. Latent profile 2 did not differ from latent profile 1 in any sociodemographic data but had significantly worse values of a moderate-to-large effect size in history of abuse, number of previous episodes of depression and suicide, stigmatisation, self-blame, negative and un-resolution rumination, residual symptom severity at intake and in the distal quality-of-life variables. Supplementaries S15 and S16 provide details on the latent profiles and their baseline characteristics by trial arm.

### Latent growth curve

Supplementary Material S17 provides details of the LGCM analysis. The quadratic (heteroscedastic auto-correlated) LGCM outperformed both the intercept-only and the linear models, indicating a curvilinear trend with a decreasing initial phase that diminishes and may eventually turn into an increase of symptoms (Fig. 1(b)). The LGCM suggested individual variability in the initial level of residual depressive symptoms (estimated BDI-II mean = 13.64, which is around the cut-off that differentiates mild from minimal depression). However, there was no significant variability either in the linear rate of change (estimated BDI-II mean = −0.30, suggesting an average decrease of 0.3 BDI-II points per unit of time, i.e. per month) or in the quadratic rate of change (estimated BDI-II mean = 0.01).

### Moderated-mediation

**Fig. 2:**
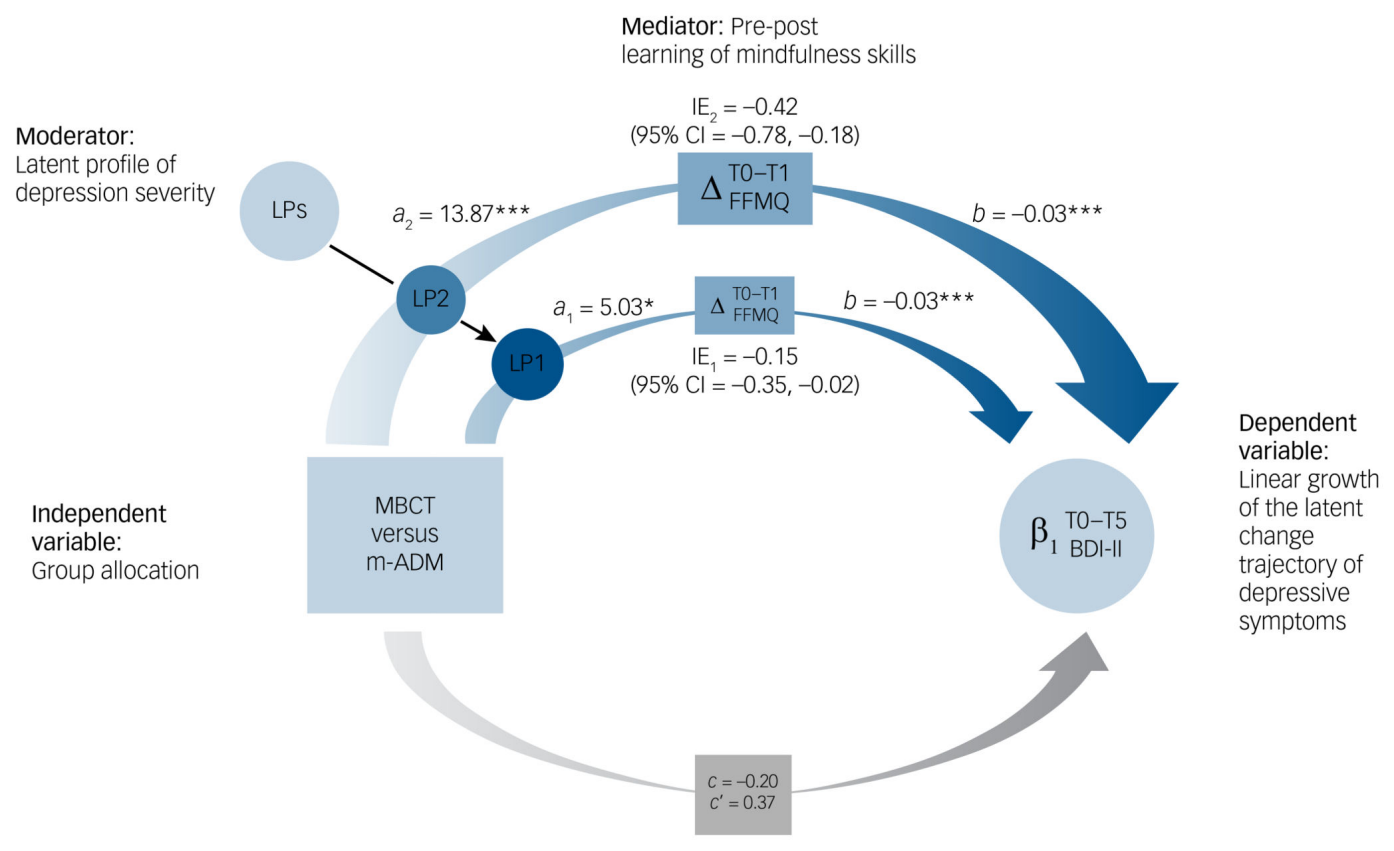
Latent profiles moderating the mediation of change in mindfulness skills on depressive symptoms. MBCT, mindfulness-based cognitive therapy with support to taper or discontinue antidepressant medication; m-ADM, maintenance antidepressant medication; LPs, latent profiles; LP1, latent profile 1 (less severe history of depression); LP2, latent profile 2 (more severe history of depression). ΔFFMQ, baseline to one month after the end of the MBCT training (T0–T1) change in mindfulness skills. β_1_ (BDI-II), linear slope of depressive symptoms (i.e. rate of change in depressive symptoms, as measured by the Beck Depression Inventory-II) over time from baseline to two-years follow-up (T0–T5). *a*_1_, allocation group → ΔFFMQ path for LP1. *a*_2_, allocation group → ΔFFMQ path for LP2. *b*, ΔFFMQ → β_1_ (BDI-II) path (this is shared across LPs, as there were no moderating effects in this path). IE_1_, indirect effect for LP1. IE_2_, indirect effect for LP2. 95% CI, bootstrapped 95% confidence interval for the indirect effect. *c*’, direct effect of allocation group on β_1_ (BDI-II) after adjustment for the mediating effects (this is shared across LPs, as there were no moderating effects in this path). *c*, total effects. Coefficients are not standardised and therefore are shown in the original units of the variables used in the moderated-mediation model. The colour transition of indirect effects represents a potential large shift from a mild-to-moderate baseline level of depressive symptoms (BDI-II mean = 19.37) to a minimal level of depressive symptoms at the 24-month follow-up (BDI-II mean = 9.29) for LP2. For LP1, it indicates a small-to-moderate effect in the minimal range of depressive symptoms between baseline (BDI-II mean = 10.71) and 24-months follow-up (BDI-II mean = 7.11).^30^ * *P* < 0.05; ** *P* < 0.01; *** *P* < 0.001. Image created with support of Delphine Perrot.

Descriptive statistics for the change in mindfulness skills during the intervention by trial arm and latent profile are provided in Supplementary Material S18. As shown in Table 2 and Fig. 2, a significant moderation effect was observed between trial arm and latent profile in the *a*-path, from trial arm to change in mindfulness skills (coefficient = 8.84; 95% CI = 0.26, 17.32; *P* = 0.043) (Supplementary Material S19). The conditional effect from trial arm to change in mindfulness skills was most pronounced for higher severity of depression (latent profile 2: *a*_2_ = 13.87; 95% CI = 7.80, 20.20; *P* < 0.001) but was also significant for lower severity of depression (latent profile 1: *a*_1_ = 5.03; 95% CI = 0.26, 10.09; *P* = 0.045).

The effect from change in mindfulness skills to the linear slope of depressive symptoms over time was *b* = −0.03 (95% CI = −0.25, −0.02; *P* < 0.001). This effect was not moderated by the latent profiles (coefficient = −0.001; 95% CI = −0.04, 0.04; *P* = 0.949) (Supplementary Material S20). The index of moderated-mediation was significant (coefficient = −0.27; 95% CI = −0.66, −0.03), providing evidence for the latent profiles to moderate the mediating effect of change in mindfulness skills between trial arm and rate of change in depressive symptoms. The conditional indirect effect for higher severity of depression (latent profile 2) was the strongest (indirect effect = −0.42; 95% CI = −0.78, −0.18). For lower severity of depression (latent profile 1), it was weaker but significant (indirect effect = −0.15; 95% CI = −0.35, −0.02). For the direct effect from trial arm to the linear slope of depressive symptoms over time, there was no significant moderation between trial arm and latent profile (coefficient = −0.02; 95% CI = −1.08, 1.02; *P* = 0.965). The direct effect from trial arm to the linear slope of depressive symptoms over time, after controlling for the indirect effects, was not significant (*c*′ = 0.37; 95% CI = −0.04, 0.77; *P* = 0.080), and in the opposite direction to the total effects (*c* = −0.20; 95% CI = −0.68, 0.25; *P* = 0.398). Around 10% of the variance in the linear slope of change in depressive symptoms over time was explained by trial arm and change in mindfulness skills. Within this variance, the indirect effect explained a small amount in latent profile 1 (2%) and an intermediate amount in latent profile 2 (18%).

Descriptive data for m-ADM discontinuation and home-based formal meditation practice, by trial arm and latent profile, are presented in Supplementary Material S21. Results for the moderated-mediation sensitivity analysis are provided in Supplementary Material S22. After adjusting for the potential confounders, a significant moderation effect was observed between trial arm and the latent profiles in the *a*-path from trial arm to change in mindfulness skills (coefficient = 14.00; 95% CI = 3.00, 25.96; *P* = 0.016). The index of moderated-mediation after adjusting for the potential confounders was significant (coefficient = −0.37; 95% CI = −0.89, −0.08). This provided further evidence for the latent profiles to moderate the mediating effect of change in mindfulness skills between trial arm and rate of change in depressive symptoms, favouring the higher severity of depression profile (latent profile 2).

## Discussion

We know that both m-ADM and MBCT are effective treatments for recurrent depression,^3,7,9,30^ but there is a need to better understand which works best for which patient profile, as well as how MBCT provides its benefits. This understanding is crucial for personalising prevention efforts, thereby increasing acceptability and optimising their effectiveness. In line with previous research,^31,32^ we found two relevant subgroups of individuals with recurrent depression in remission, that can be differentiated in terms of the severity of their clinical history, psychological characteristics and impairment. This heterogeneity was also evident in the starting point (i.e. intercept) of the time series for residual depressive symptoms, showing clear differences between both subgroups.

We found evidence that mindfulness skills are a potential mediator through indirect mediation only (i.e. significant indirect effects were observed, but neither the direct effect, after controlling for the indirect effects, nor the total effects were significant).^33^ This suggests that mindfulness skills are a unique mechanism of action to this MBCT-tapering support intervention (versus m-ADM) for treating residual depressive symptoms in recurrent depression. This finding aligns with the originally hypothesised theoretical framework^8^ and supports the conclusions of recent reviews.^10,34^ As expected,^12,18–23^ the mediating effect was stronger in the subgroup of participants with a more severe history of depression. Our results suggest this is because these individuals are learning mindfulness skills. Other variables such as rumination, self-criticism or positive affect, which define severity profiles, might also act as mechanisms of change in MBCT. This dual role requires further investigation.

The impact of MBCT through mindfulness skills (i.e. the indirect effect) was three times more pronounced in the more severe subgroup of people with recurrent depression. This resulted in a clearly clinically relevant expected mean reduction of around ten points on the BDI-II for the more severe profile, suggesting large effects and a potential shift from a mild-to-moderate baseline level of depressive symptoms to a minimal level of depressive symptoms at 24-month follow-up. However, for the less severe profile, the expected mean reduction was around 3.5 points on the BDI-II, reflecting small-to-moderate effects and a potential (clinically relevant) shift within the minimal range of symptoms. These findings suggest that the subgroup of participants with recurrent depression and a more severe profile should be the optimal target population for MBCT. However, we do not know if effects would hold with those having a current depressive episode, although there is evidence for the efficacy of MBCT in reducing current depressive symptoms.^35^ For individuals with a less severe profile, other evidence-based approaches such as cognitive therapy, physical exercise and lifestyle changes might be beneficial.^36^ There is also preliminary evidence showing that innovations in cognitive therapy and MBCT that focus on lifestyle changes and enhancing positive affectivity can confer benefits (e.g. MBCT Finding Peace, MBCT For Life, MBCT Taking it Further).^37–39^

### Strengths and limitations

This study offers a unique opportunity to examine MBCT’s pathways of change by using a large RCT with process elements and innovative statistical approaches to answer for whom and how MBCT leads to the management of depressive symptoms in recurrent depression. Although mediation can be difficult to detect,^40^ we have utilised a sophisticated analytical approach that considers moderated-mediation to shed light on this process. Further strengths include the use of an evidence-based active control group (m-ADM), which effectively addresses depressive symptoms through different mechanisms and our 24-month follow-up period. Nevertheless, the representativeness of the study sample (which included people with recurrent depression fully or partially remitted, predominately female, and white British), and in turn, generalisability of our findings, are key limitations. We did not have enough numbers to address gender-based analyses, which is a limitation. In addition, we carried out exploratory analyses without adjusting for multiple comparisons. While the use of 95% CIs provides an indication of the precision and stability of our findings, future replication studies are needed. The m-ADM group received less attention compared with the MBCT-tapering support group; consequently, effects might be attributed to increased contact with services. Finally, although moderated-mediation effects persisted after controlling for the discontinuation of ADM, future studies need to incorporate interaction analyses to determine whether the effects of MBCT vary depending on whether or not people are taking medication.

## Conclusions

Individuals with recurrent depression and a more severe history of depression, partially remitted with mild-to-moderate residual symptoms, may benefit most from MBCT-tapering support in the management of residual depressive symptoms by the acquisition of mindfulness skills. Enhancing MBCT to focus on the acquisition and use of mindfulness skills might lead to further improvements. Here we provide initial evidence for the benefit of a more personalised approach to the management of recurrent depression, that may be informed by patient treatment preferences and clinical characteristics.

## Supplementary Material

1

## Figures and Tables

**Table 1:** Baseline characteristics of participants by allocation group

	m-ADM (*n* = 212)	MBCT (*n* = 212)
Demographic characteristics		
Age, years, mean (s.d.)	48.71 (12.73)	50.16 (11.85)
Gender, Women, *n* (%)	174 (82.1)	151 (71.2)
Ethnicity, White, *n* (%)	210 (99.1)	210 (99.1)
Marital status		
Single, *n* (%)	38 (17.9)	42 (19.8)
Married, cohabiting, civil partnership, *n* (%)	140 (66.0)	125 (59.0)
Separated, divorced, widowed, *n* (%)	33 (15.6)	44 (20.8)
Education		
No educational qualification, *n* (%)	10 (4.7)	10 (4.7)
O levels or GCSEs, *n* (%)	45 (21.2)	36 (17.0)
AS and A levels or vocational qualification, *n* (%)	92 (43.4)	84 (39.6)
University training, *n* (%)	61 (28.8)	77 (36.3)
Employment status		
Not working, *n* (%)	82 (38.7)	98 (46.2)
Part time, *n* (%)	70 (33.0)	53 (25.0)
Full time, *n* (%)	59 (27.8)	59 (27.8)
Quality of life		
Quality-of-life rating (range: 1–5), mean (s.d.)	3.72 (0.83)	3.66 (0.80)
Health satisfaction (range: 1–5), mean (s.d.)	3.07 (0.99)	2.92 (1.03)
Physical (range: 4–20), mean (s.d.)	14.41 (5.06)	14.47 (5.51)
Psychological (range: 4–20), mean (s.d.)	12.32 (2.59)	12.61 (2.62)
Social (range: 4–20), mean (s.d.)	13.12 (3.42)	13.39 (3.41)
Environment (range: 4–20), mean (s.d.)	15.08 (2.56)	15.03 (2.39)
Mindfulness skills		
FFMQ (range: 39–195), mean (s.d.)	117.94 (17.22)	119.26 (18.69)
Depressive symptoms		
BDI-II (range: 0–63), mean (s.d.)	14.45 (10.07)	13.77 (10.21)

m-ADM, maintenance antidepressant medication; MBCT, mindfulness-based cognitive therapy (with support to taper or discontinue antidepressant medication); GCSE, General Certificate of Secondary Education; AS and A levels, Advanced Subsidiary (AS) and Advanced (A) levels; FFMQ, Five Facets of Mindfulness Questionnaire (higher scores mean higher levels of mindfulness skills); BDI-II, Beck Depression Inventory-II (higher scores mean higher levels of depressive symptoms). Quality-of-life was measured using the World Health Organization Quality-of-Life instrument (WHO-QOL-BREF, with higher scores meaning better quality of life). In m-ADM, 1 participant did not provide data on marital status, 4 participants did not provide data on education, 2 participants did not provide data on employment status, 7 participants did not provide data on any quality-of-life measure, 10 participants did not provide data on FFMQ and 6 participants did not provide data on BDI-II. In MBCT, 1 participant did not provide data on marital status, 5 participants did not provide data on education, 2 participants did not provide data on employment status, 3 participants did not provide data on any quality-of-life measure, 5 participants did not provide data on FFMQ and 2 participants did not provide data on BDI-II.

**Table 2:** Moderated-mediation of mindfulness skills

Direct effects	Coefficient	s.e.	Boot 95% CI	*p*
Allocation group * LP → ΔFFMQ	8.84	4.38	0.26, 17.32	0.043
Allocation group → ΔFFMQ (LP1)	5.03	2.52	0.26, 10.09	0.045
Allocation group → ΔFFMQ (LP2)	13.87	3.20	7.80, 20.20	<0.001
ΔFFMQ * LP → β_1_(BDI-II)	−0.001	0.02	−0.04, 0.04	0.949
ΔFFMQ → β_1_(BDI-II)	−0.03	0.01	−0.25, −0.02	<0.001
Allocation group * LP → β_1_(BDI-II)	−0.02	0.53	−1.08, 1.02	0.965
Allocation group → β_1_(BDI-II)	0.37	0.21	−0.04, 0.77	0.080
Total	−0.20	0.24	−0.68, 0.25	0.398
Indirect effects	Coefficient	s.e.	Boot LLCI	Boot ULCI
LP1	−0.15	0.08	−0.35	−0.02
LP2	−0.42	0.15	−0.78	−0.18
Difference (index of moderated-mediation)	−0.27	1.92	−0.66	−0.03

Allocation group, m-ADM (maintenance antidepressant medication) versus MBCT (mindfulness-based cognitive therapy) with support to taper or discontinue antidepressant medication. s.e., standard error. Boot LLCI, Bootstrap Lower Limit of (95%) CI; Boot 95% CI, Bootstrap 95% CI; Boot ULCI, Bootstrap Upper Limit of (95%) CI; LP, latent profile; LP1, latent profile 1; LP2, latent profile 2. ΔFFMQ, pre-post (T0–T1) change in mindfulness skills. β_1_ (BDI-II), linear slope of depressive symptoms over time (T0–T5), as measured by the Beck Depression Inventory-II. Difference (index of moderated-mediation), indirect effects difference by latent profile. Coefficients are not standardised, and therefore maintain the original units of the variables involved in the regression. LP1: *R*^2^(mediator) = 0.02; *R*^2^(dependent variable) = 0.10. LP2: *R*^2^(mediator) = 0.15; *R*^2^(dependent variable) = 0.10.

## Data Availability

J.M.-M. and W.K. had full access to all the data in the study and take responsibility for the integrity of the data and the accuracy of the data analysis. J.M.-M. and W.K. claim that the manuscript is an honest, accurate and transparent account of the study being reported, that no important aspects of the study have been omitted and that any discrepancies from the study as planned have been explained throughout the manuscript. Authors were not precluded from accessing data and they accept responsibility to submit for publication. The de-identified baseline data and codebook from the PREVENT trial, and analytic codes and research materials are available from the corresponding author, W.K. (willem.kuyken@psych.ox.ac.uk) upon reasonable request (release of data are subject to an approved proposal and a signed data access agreement).

## Relevance statement

This study investigates the distinct mediating role of mindfulness skills in treating recurrent depression with mindfulness-based cognitive therapy (MBCT), compared with maintenance antidepressant medication (ADM), based on the varying severity of the depressive clinical history. Findings reveal mindfulness skills as a pivotal mechanism driving change in MBCT, with particularly beneficial effects for people with higher severity of depression. Psychiatrists can utilise MBCT tailored to individual severity profiles for those individuals who do not accept ADM, offering personalised management for recurrent depression. This research underscores the importance of integrating MBCT into psychiatric practice, enhancing treatment efficacy and patient outcomes.
