# Supplementary material for: Examining what works for whom and how in mindfulness-based cognitive therapy (MBCT) for recurrent depression: moderated-mediation analysis in the PREVENT trial
Source: Br J Psychiatry. 2024 Nov 8;226(4):213–21. doi: 10.1192/bjp.2024.178 (PMC7617292; doi:10.1192/bjp.2024.178)
Supplement: Montero-Marin et al. supplementary material [file S0007125024001788sup001.docx]

**Supplementary Materials**

**Examining What Works for Whom and How in Mindfulness-Based Cognitive Therapy (MBCT) for Recurrent Depression: A Moderated-Mediation Analysis in the PREVENT Trial**

Jesus Montero-Marin, Verena Hinze, Shannon Maloney, Anne Maj van der Velden, Rachel Hayes, Edward R. Watkins, Sarah Byford, Tim Dalgleish, Willem Kuyken,

**Table of Contents**

page

**Supplement S1: Adaptations in MBCT-TS from the original MBCT programme…………......….…3**

**Supplement S2: MBCT Teacher competency across the 42 sampled sessions of the trial…...…4**

**Supplement S3: Measures………………………………………………………………………….………….……….……5**

**Supplement S4: Details of latent profile and latent growth curve analyses…….……..…………….9**

**Supplement S5: Details of the moderated-mediation analyses……………………….………..…….….13**

**Supplement S6: Adherence to treatment by intervention group…………………….……….….………17**

**Supplement S7: Self-reported antidepressant medication use over time by group……….…….18**

**Supplement S8: Number of visits to the GP by group over the study period………………….……22**

**Supplements S9-S13: Participant baseline characteristics by trial arm and follow-up status...16**

**Supplement S14: Details for the latent profile analyses results…………………………………….…….28**

**Supplement S15: Latent Profiles and Baseline Characteristics by trial arm…………………….……40**

**Supplement S16: Latent Profiles and Predictive Factors by Trial Arm………………………….………41**

**Supplement S17: Details for the latent growth curve model results…………………………..……….42**

**Supplement S18: Descriptive statistics for pre-post change in mindfulness skills (FFMQ) by group and LP………………………………………………………………………………………………………………….…..52**

**Supplement S19: Baseline to post-treatment change of mindfulness skills (FFMQ) as a function of treatment received and LP…………………………………………………………………..……………53**

**Supplement S20: Linear slope of depressive symptoms over time as a function of change of mindfulness skills (FFMQ) and LP……………………………………………………………………..……..…………54**

**Supplement S21: ADM use and formal meditation practice by trial arm and LP…………….……55**

**Supplement S22: Moderated-mediation sensitivity analysis……………………………………….………56**

**References……………………………………………………………………………………………………………………….…60**

**Supplement S1: Adaptations in MBCT-TS from the original MBCT programme**

| Rationale | Adaptation | Implemented |
| --- | --- | --- |
| Preparation for tapering of medication (session 6) by increasing understanding of relapse/recurrence and ways of acting at an earlier stage | Completing a short questionnaire before the orientation session around relapse/recurrence ways of acting | In the orientation session prior to the group starting |
|  | Broadening relapse/recurrence signature to include responding and acting in line with the 8-week course |  |
|  | Basing the learning about participants’ depression at orientation around their relapse/recurrence signature |  |
|  | Having the relapse/recurrence signature and ways of responding/acting as work  in progress throughout the course | Referring to the relapse/recurrence signature at appropriate moments in inquiry throughout the course |
|  |  | Inviting dialogue as part of the home practice review (from session 3) |
| Involving GPs in the participants’ response plan | Inviting participants to provide a copy of their plan for us to send to their GP on completion of the group | Relapse/recurrence action plan introduced at the orientation session |
|  |  | Plan collected at first follow-up |
| Supporting participants around the early stages of tapering | First follow-up session shortly after the group ended (3–5 weeks) | 3–5 weeks after session 8 |
|  | Maintaining practice, turning towards the difficult and adding to relapse/recurrence signatures. | This session was with the original group, but subsequent follow-up sessions involved merged groups |
| Allowing participants to track their process with awareness, to illustrate the potential for relapse/recurrence around a drop in mood and how mindfulness may offer the possibility of somewhere else to stand rather than being dragged down the spiral | Sequence: Automatic thoughts questionnaire, brief inquiry, breathing space, brief inquiry, watching slides from The Black Dog book, brief inquiry, walking practice, brief inquiry, ‘Healing from Within’ DVD, and summary. | Session 4 |
| Allowing more space for working with difficulty | Simplifying the sitting practice to include breath, body and working with difficulty (optional CD ‘Exploring the Difficult’ for home practice) | Session 5 |

**Supplement S2: MBCT Teacher competency across the 42 sampled sessions of the trial**

|  | N (%) for each rating across 42 tapes | | | | | |
| --- | --- | --- | --- | --- | --- | --- |
| Domains | Incompetent | Beginner | Advanced Beginner | Competent | Proficient | Advanced |
| Coverage, pacing and organisation of the session curriculum | 0 (0) | 0 (0) | 1 (2.4) | 5 (11.9) | 14 (33.3) | 22 (52.4) |
| Relational skills | 0 (0) | 0 (0) | 1 (2.4) | 8 (19.0) | 10 (23.8) | 23 (54.8) |
| Embodiment | 0 (0) | 0 (0) | 1 (2.4) | 8 (19.0) | 13 (31.7) | 19 (46.3) |
| Guiding mindfulness practices | 0 (0) | 0 (0) | 3 (7.1) | 1 (2.4) | 15 (35.7) | 23 (54.8) |
| Conveying course themes through interactive inquiry and didactic teaching | 0 (0) | 0 (0) | 2 (4.8) | 7 (16.7) | 15 (35.7) | 18 (42.9) |
| Facilitation of the group learning environment | 0 (0) | 0 (0) | 2 (4.8) | 12 (28.6) | 11 (26.2) | 17 (40.5) |

Note. Under the domain of embodiment there was one occasion when the external assessor felt unable to rate.

**Supplement S3: Measures.**

The following sociodemographic were collected at baseline (prior to randomization): age (years), self-identified gender (male, female), self-identified ethnicity (White, Asian, Black, other), level of education (no qualifications, Ordinary Levels, Advanced Subsidiary levels, National Vocational Qualifications or other vocational qualification, University Bachelor’s degree, University Master’s degree, University professional training or PhD), relationship status (single, divorced, widowed, cohabiting, civil partnership, married), and employment status (not working, part time, full time).

• Informed by previous theoretical and empirical research looking for potential predictive variables of depression recurrence and treatment response comparing m-ADM vs. MBCT,^1-7^ we made use of a series of variables measured at trial baseline to explore and confirm a potential latent profile (LP) of patients that might be demarcated by a more severe history of depression in terms of adversity, longevity, and entrenchment, and for which MBCT might confer a better prognosis. For that, we included (*i*) markers of symptoms intensity and clinical history, (*ii*) cognitive and emotional factors, as well as (*iii*) relational and social variables, that were all assessed prior to randomization. These variables (which are described below) were selected because (a) they are usually available in clinical practice and are commonly included as part of routine diagnostic procedures, and (b) they have demonstrated a significant predictive value in terms of depression recurrence and/or treatment response.^1-7^

(*i*) Symptoms intensity and clinical history included *Clinician-Rated Symptoms of Depression* by means of the Hamilton Depression Rating Scale (HAMD) structured clinical interview.^8^ The HAMD is a widely used interview for assessing the severity of depressive symptoms in individuals diagnosed with depression. It consists of 17 items that cover various aspects of depression, such as mood, guilt, suicide ideation, insomnia, agitation or retardation, and other physical symptoms. Each item is scored based on the intensity or frequency of the symptom, ranging from 0 (“not present”) to 4 (“severe”), and the total score is used to categorize the severity of depression (0-7: no depression; 8-16: mild depression; 17-23: moderate depression; ≥24: severe depression). Recently, it has been observed certain heterogeneity among depressed outpatients considered to be in remission. This has raised the suggestion of using a lower cut-off to define remission, with remitters further subdivided into two groups, remitters with (scores: 3-7) and without (scores: 0-2) mild residual symptoms.^9^ The scale was administered by a trained clinician during a structured interview, providing a standardized way to quantify the severity of depressive symptoms. We included a measure of *Childhood Abuse* as reported by the Measure of Parenting Scale (MOPS).^10^ The MOPS is a self-report measure that allows to evaluate the levels of parental abuse experienced as a child. Participants need to indicate to what extent 15 statements about their mother’s and father’s (30 items total) were true for the first 16 years of their lives. Responses to the statements are rated from 0 (“not at all true”) to 3 (“extremely true”). Following Cohen et al. (2023),^1^ a median split was used to categorize participants as high or low in childhood abuse. We also included the *Age of First-Onset of Depression* (years), *Number of Previous Depressive Episodes* that lasted for 24 months or more, *Severity of Last Episode* (the number of Structured Clinical Interview for DSM- IV (SCID) symptoms present when considering the most recent depressive episode,^11^ with a range between 5 and 9), *Chronicity of Last Episode* (months), *Previous Suicide Attempt* (whether the patient has previously attempted suicide or not), and the Number of Comorbid DSM-IV axis I Psychiatric Diagnoses (SCID) (*Comorbidities*).^11^

(*ii*) Cognitive and emotional factors were composed by self-report measures of potential mechanistic variables of treatment efficacy.^1^ They included dispositional tendencies for brooding as measured by the *Negative Rumination* and *Ongoing Un-resolution rumination* subscales of the Cambridge-Exeter Repetitive Thought Scale (CERTS).^12^ The negative rumination and ongoing un-resolution rumination subscales of the CERTS self-report measure include a total of 20 and 4 items respectively. They both are rated from 1 (“almost never”) to 5 (“almost always”), with a total range from 20 to 100, and 4 to 20, respectively. We also included the *Self-Blame* and *Lack of Acceptance* subscales from the Cognitive Emotion Regulation Questionnaire (CERQ).^13^ These subscales assess maladaptive emotion regulation strategies when participants are confronted with negative events. The self-blame and lack of self-acceptance subscales from the CERQ include 4 items each. Items are ranged from 1 (“almost never”) to 5 (“almost always”), with a total range from 4 to 20 for both self-blame and lack of self-acceptance. We measured the *Ability to Recognise Early Warning Signs of Depression* using a bespoke single item (“How well do you recognise early signals of depression?”) that was responded as follows: 0 (“I don't see the early warning signs coming at all”), 1 (“When I see the early warning signs, I find I can't do anything about it”), 2 (“When I see the early warning signs, I have tried to respond healthily but it only helps a bit”), 3 (“When I see the early warning signs, I have responded healthily more times than not”), 4 (“When I see the early warning signs, I am now in the habit of responding healthily”). We included a measure of *Awareness* using the acting with awareness subscale from the Five Facet Mindfulness Questionnaire (FFMQ).^14^ This subscale includes 8 items to measure to what extent participants attend to what is happening in the present moment experience. Participants rate the extent to which they experience the state of awareness scoring from 1 (“never or very rarely true”) to 5 (“very often or always true”), with a total range from 8 to 40. We measured *Perceived Self-Efficacy* using the General Self-Efficacy Scale (GSE).^15^ The GSE is a ten-item self-report questionnaire that assesses individual’s sense of self-efficacy over the past two-week period. Participants answered the scale using a Likert-type format from 1 (“definitely disagree”) to 5 (“definitely agree”), with higher scores representing a higher sense of self-efficacy. Finally, following on an earlier work,^16^ we measured *Positive Affectivity* by pooling the joy and contentment subscales of the Dispositional Positive Emotion Scale (DPES).^17^ We used a modified Likert-type scale for each item on the DPES, asking participants to state if they “strongly disagreed” (1), “disagreed” (2), “were neutral” (3), “agreed” (4), or “strongly agreed” (5) with each statement. These items were summed to form an index composite of positive affect, with scale scores ranging between 11 and 55.

(*iii*) Relational and social variables included *Relationship Satisfaction*, using a bespoke 7-item questionnaire in which participants were asked to complete thinking of the most important relationships in their lives. Items (“Taking all things together, I am satisfied with this relationship”, “We are able to talk about and work through any worries and problems in our relationship”, “I feel ‘tuned in’ to this person”, “I feel special and valued in this relationship”, “I am able to take care of my needs in this relationship”, “I balance taking care of myself with taking care of this person”, “I feel confident of myself in the relationship”) were scored on a Likert-type scale ranging from 1 (“almost never”) to 5 (“almost always”). *Stigmatization and Normalization* was measured using a bespoke 7-item questionnaire in which participants indicate how often they experienced stigmatization due to their depression. Items (“I feel out of place in the world because I have a history of depression”, “Having depression has spoiled my life”, “People who have never had depression could not possibly understand me”, “I am embarrassed or ashamed that I have problems with depression”, “I am disappointed in myself for having problems with depression”, “I feel inferior to others who don’t have depression”, “Having problems with depression means I cannot be normal”) were scored on a Likert-type scale ranging from 1 (“almost never”) to 5 (“almost always”).

• We included a measure of *Quality of Life* at baseline using the WHO Quality of Life instrument (WHOQOL-BREF) and utilized its subdomains (e.g., quality of life, health satisfaction, physical health, psychological health, social relationships, environment/financial) as distal variables to assist in characterizing, validating, interpreting, and understanding the identified latent profiles.^18^ Scores for the subdomains range from 1 to 5, with higher scores meaning a better quality of life.

• We measured *Mindfulness Skills* using the Five Facet Mindfulness Questionnaire (FFMQ) total score,^14^ as a potential mediating variable measured at baseline and at one month after the end of the MBCT training (or the equivalent time in the m-ADM arm). The FFMQ is a self-report questionnaire that is composed by 39 items designed to measure five facets of mindfulness: observing, describing, acting with awareness, non-judging of inner experience and non-reactivity to inner experience. Observing reflects the ability to attend to or notice internal and external experiences (sensations, thoughts, or emotions), without necessarily reacting to them. Individuals scoring high in this facet are generally good at being aware of the details of their present moment experience. Describing involves the ability to express or put into words one's internal experiences. People high in this facet are able to articulate and describe their thoughts and feelings with clarity. Acting with awareness assesses the degree to which an individual is fully present and engaged in their activities, rather than being on "autopilot" or engaging in activities without full awareness. It involves being conscious and attentive to one's actions in the present moment. Non-judging refers to the tendency to observe one's thoughts and feelings without evaluating them as positive or negative. Individuals scoring high in this facet can experience thoughts and emotions without getting caught up in self-criticism or harsh judgment. Non-reactivity involves the ability to allow thoughts and feelings to come and go without getting overly caught up in or attached to them. People high in this facet can observe their thoughts and emotions without reacting impulsively or becoming overwhelmed by them. The FFMQ total score ranges from 39 to 195, with higher scores indicating higher levels of mindfulness skills.

• *Depressive Symptoms* (outcome variable) were measured using the total score of the 21-item self-report Beck Depression Inventory (BDI-II).^19^ The BDI-II requires participants to endorse symptoms levels ranging from 0 (“not present”) to 3 (“severe”), with a total range from 0 to 63 (0-13: minimal depression; 14-19: mild depression; 20-28: moderate depression; 29-63: severe depression). The BDI-II has demonstrated high convergent validity, and robust psychometric properties, including strong internal consistency and test-retest reliability.^20^ Participants were assessed on the BDI-II at baseline, and then one month after the end of the MBCT training. BDI-II follow-up measures were also taken at 9, 12, 18, and 24 months after randomization.

**Supplement S4: Details of latent profile and latent growth curve analyses.**

*Latent profile analysis*

We carried out a latent profile (LP) analysis that was developed in three steps to identify a distinct subgroup of individuals with recurrent depression in remission, demarcated by a more severe clinical history of depression in terms of adversity, longevity, and entrenchment. It has been proposed that heterogeneity (i.e., variance in psychosocial function and impairment) exists within the group of patients in remission of depression, leading to two potential subgroups: partial remitters (i.e., those with mild residual symptoms) and full remitters (those who have achieved a more complete remission with minimal residual symptoms).^9^ Building on this notion, we hypothesised that this heterogeneity can be captured by a two-profile solution. Specifically, one profile was anticipated to manifest a more severe clinical history of depression, while the other was expected to exhibit a less severe clinical history of depression. We hypothesized that this two-profile solution demonstrates an adequate fit, providing a more nuanced understanding of the potential differential effectiveness of treatments in terms of symptom trajectories within the group of patients in remission of depression. We were interested in profiles that are optimally separated and likely to reflect 'true' classes within the population, making them easier to identify clinically, rather than focusing on the entire spectrum of potential heterogeneity. Additionally, we also aimed to maintain greater statistical power, when considering subgroups (i.e., LPs) in subsequent analyses.

First, we randomly selected a sub-sample (split-half) and evaluated a series of LP models, that were fitted using maximum likelihood (ML) estimation and contained one to five latent profiles. Second, we evaluated the replicability of the models in the second half of the sample. All subsequent analyses were then developed with the total sample. Each model was estimated using 10,000 random sets of start values with 500 iterations, of which 500 of the best solutions were retained. To inform model selection, following Morin et al. (2016),^21^ we used the Akaike information criterion (AIC), consistent Akaike information criterion (CAIC), bayesian information criterion (BIC), sample-size-adjusted BIC (sBIC), Lo–Mendell–Rubin adjusted likelihood ratio test (LMR-LRT), and bootstrapped likelihood ratio test (BLRT), and we calculated the index of classification accuracy (Entropy).^22^ We considered the interpretability (e.g., meaning) and parsimony (e.g., percentage of participants) in each profile.^22^ Posterior probabilities were also inspected, such that a value of no less than 0.70 indicated a good level of confidence in the assigned profile memberships.^23^ We assigned patients into their most likely LP at baseline based on the BCH weights approach, which accounts for the measurement error of the LP variable.^24^ Although little is known about the exact effect of sample size on the ability to identify the number of underlying latent subgroups, more recent research has shown promising advances in this regard.^25-28^ The required sample size depends on the number of LPs and the distance between them, which is typically unknown in advance.^28^ A median sample size of n = 377 has been reported across 38 different studies,^29^ and simulation studies have suggested that samples ranging from 300 to 500 would be appropriate.^22,28,30,31^

*Latent growth curve analysis*

A series of univariate latent growth curve models (LGCMs) were tested to determine the overall trajectory of depressive symptoms from baseline to 24-month follow-up. First, a model specifying only an intercept was examined (i.e., unconditional, or intercept-only model), and then linear and quadratic growth factors were added sequentially. The intercept-only model assumes that there is no systematic change in depressive symptoms over time, implying a constant initial level (β_0_, i.e., intercept) for all individuals. The linear LGCM assumes that change over time is characterized by a constant and linear progression, and that can be represented by the intercept (capturing initial status) and the linear slope (β_1_, representing a consistent rate of change). Finally, the quadratic LGCM assumes that change over time follows a curvilinear pattern and incorporates the intercept, linear slope, and quadratic slope (β_2_, representing acceleration or deceleration in the rate of change).

In parallel, we adopted a progressive approach to understanding and modelling different aspects of the structure of error terms. To achieve this, we evaluated the fit of homoscedastic, heteroscedastic, and auto-regressed models. This sequential structure represents an extension of the previous one, with each model incorporating additional elements to capture potential complexities in the data. The homoscedastic model assumes equal error variances over time, serving as a simplified baseline for later comparisons. The heteroscedastic model relaxes the assumption of homoscedasticity, allowing error variances to vary over time and potentially improving the model if evidence of variation in error variances over time is present. The auto-regressed model builds on the heteroscedastic model, introducing an autocorrelation structure between consecutive errors over time. This allows the capture of temporal patterns in error variability, making it a more complex extension of the heteroscedastic model. A graphical representation of all these models, including details on the latent factors (e.g., intercept, linear and quadratic slopes, error terms), the parameters being estimated (means, variances, covariances), and how the factor loadings for the latent factors represent different temporal trends and data structures, can be found in *Figure S4a.* As standardizing the manifest variables destroys the ability to examine change because the standardization equates the means and variances,^32-34^ we present the unstandardized estimates, as it is most typical in this type of research.

We used ML estimation to analyse the data, given that it facilitates model comparison and thus aids model selection. Model fit was evaluated using common global fit indices (e.g., Comparative Fit Index (CFI), Tucker-Lewis Index (TLI), Root Mean Square Error of Approximation (RMSEA) with a 90% Confidence Interval (90% CI), Standardized Root Mean Square Residual (SRMR)), and model comparisons were made using likelihood-based indices (e.g., AIC, CAIC, BIC, sBIC). CFI and TLI values greater than 0.90 are considered adequate, and values greater than 0.95 are good. Similarly, RMSEA and SRMR values less than 0.05 are considered good, values less than 0.08 are considered adequate, and values less than 0.10 are considered marginal.^35^ For AIC, CAIC, BIC, and sBIC, the preferred model is the one with the lowest value, indicating balance between goodness of fit and model complexity.^36^ It has been found that LGCMs has adequate power to model mediation relationships when the sample size is at least n = 200 and for at least five time-points.^37^

Figure S4*a*: *Latent growth curve models tested.*

| M1: Intercept-only   \|  \|  \|  \| \| --- \| --- \| --- \|   *M1.1: Homoscedastic M1.2: Heteroscedastic M1.3: Heteroscedastic & auto-correlated* |
| --- | --- | --- | --- |
| M2: Linear LGCM   \|  \|  \|  \| \| --- \| --- \| --- \|   *M2.1: Homoscedastic M2.2: Heteroscedastic M2.3: Heteroscedastic & auto-correlated* |
| M3: Quadratic LGCM   \|  \|  \|  \| \| --- \| --- \| --- \|   *M3.1: Homoscedastic M3.2: Heteroscedastic M3.3: Heteroscedastic & auto-correlated* |

The first model (M1) tests only the intercept (β_0_), representing the mean starting point of depressive symptoms across the six time periods. This model assumes there is no systematic change in depressive symptoms over time, implying a constant initial level for all participants. It constrains all factor loadings to 1, forcing them to be equal to those of the first time period. Therefore, it does not include terms to model linear trends or curvatures over time. The second model (M2) incorporates two latent constructs: the intercept (β_0_) and the linear slope (β_1_). The linear slope represents the linear trajectory over time and is estimated by setting the factor loadings on the corresponding linear temporal scale. The third model (M3) tests for nonlinearity by introducing a latent quadratic slope (β_2_), in addition to the previously mentioned β_0_ and β_1_ terms. Factor loadings of the quadratic slope are fitted to the squared factor loadings of the linear slope. The *homoscedastic* model assumes that the variances of errors remain consistent across different time points, providing a straightforward and standardized reference point for subsequent model comparisons. For that, it assumes the error terms to be equal by constraining their factor loadings to 1. It is often assumed that measurement errors are homoscedastic over time. However, this assumption may be sometimes unrealistic.^38^ In contrast, the *heteroscedastic* model departs from this assumption, allowing for the possibility that the variances of errors can vary over time. This flexibility accommodates scenarios where there might be evidence suggesting fluctuations in error variances across different time periods. Finally, the *auto-correlated* model builds upon the heteroscedastic model by introducing an autocorrelation structure between consecutive error terms across time. This addition enables the model to capture temporal patterns in the variability of errors, rendering it a more complex extension compared to the previous heteroscedastic model. The intercept (β_0_) was defined as the first (pre-treatment) assessment in all the models. The means (μ, i.e., fixed effects) and variances (σ², i.e., random effects) of the intercept (β_0_), linear slope (β_1_), and quadratic slope (β_2_), as well as the covariances (σxy) between the intercept, linear slope, and quadratic slope, were all freely estimated.

**Supplement S5: Details of the moderated-mediation analyses.**

We employed moderated-mediation analysis to explore the conditional effects of a potential moderator variable (subgroups of patients demarcated by distinct histories of adversity, longevity, and entrenchment of depression; i.e., severity) on the relationships between the independent variable (trial arm), the mediator (pre-post improvements in mindfulness skills), and the dependent variable (the linear slope of the growth trajectory of depressive symptoms over time, from baseline to 24-months follow-up). This analysis extends traditional mediation approaches by investigating whether the strength or direction of the mediation pathway (i.e., indirect effect) varies under different levels of the moderator. Specifically, we aimed to discern whether the mediation process operates differently for distinct subgroups or conditions as defined by the moderator. The presence of a moderation effect suggests that the strength of the mediation is contingent upon the levels of the moderating variable, providing insights into the nuanced and potentially context-dependent nature of the mechanisms involved. A graphical representation of the moderated-mediation analysis is provided in *Figure S5a*.

Figure S5*a*: *Moderated-mediation main analysis.*

X = independent variable (i.e., trial arm: maintenance antidepressant medication (m-ADM) vs. mindfulness-based cognitive therapy plus m-ADM discontinuation (MBCT). M = mediator (i.e., pre-post improvements in mindfulness skills). Y = dependent variable (i.e., the linear slope of the growth trajectory of depressive symptoms over time, from baseline to 24-months follow-up). W = moderator (i.e., latent profiles (LPs) of patients demarcated by distinct depression severity). a = direct path between the independent variable and the mediator. b = direct path between the mediator and the outcome. c = total effects. c’ = direct effect of the independent variable on the dependent variable after adjustment for the mediating effects. w1 = interaction term between allocation group and LP in the relationship from trial arm to pre-post improvements in mindfulness skills. w2 = interaction term between allocation group and LP in the direct relationship from trial arm to the linear slope of the growth trajectory of depressive symptoms over time. w3 = interaction term between pre-post improvements in mindfulness skills and LP in the direct relationship from pre-post improvements in mindfulness skills to the linear slope of the growth trajectory of depressive symptoms over time.

As described in Supplement S4, we calculated the potential moderator using LP analysis to identify subgroups of patients demarcated by distinct depression severity across baseline characteristics. Secondly, we fitted the LGCM of depressive symptoms over time, spanning from baseline to 24-months follow-up. Finally, we employed a joint model that integrates (a) the measurement LP mixture model, (b) the LGCM of depressive symptoms over time, and (c) the auxiliary moderated-mediation model. BCH weights were used to calculate a joint model, combining the measurement LP mixture model with the LGCM and the moderated-mediation auxiliary model.^24^

In the moderated-mediation model, the LP acts as a potential moderator in the relationship between the trial arm and the linear slope (β_1_) of the latent growth trajectory of depressive symptoms over time, which is hypothesized to be mediated by pre-post improvements in mindfulness skills. The outcome was the linear slope as we were interested in the constant rate of change of depressive symptoms with respect to time. Thus, we computed interaction terms between (a) the trial arm and the LP variable for the relationship from the trial arm to pre-post improvements in mindfulness skills; (b) the trial arm and the LP variable for the direct relationship from the trial arm to the linear slope of the growth trajectory of depressive symptoms over time, after controlling for the potential indirect effects; and (c) the pre-post improvements in mindfulness skills and the LP variable for the relationship from pre-post improvements in mindfulness skills to the linear slope of the growth trajectory of depressive symptoms over time. In cases where there was a non-significant interaction term (i.e., there were no significant differences between LPs), we then adjusted the corresponding parameter to be equal across LPs for model parsimony.

As part of a sensitivity analysis, the potential confounding effects of the amount of home-based formal meditation practice during the intervention period (“not at all” = 0, “sometimes” = 1, “regularly” = 2, “more days than not” = 3) and the discontinuation (“yes” = 1, “no” = 0) of antidepressant intake throughout the study (which was encouraged after the intervention in the MBCT arm), were incorporated into the moderated-mediation model. The extent of meditation practice has been shown to influence the acquisition and development of mindfulness skills.^39^ Consequently, individuals demonstrating a higher commitment to practice may develop more robust skills, thereby impacting the relationship between the intervention and improvements in mindfulness skills. Moreover, those with a more substantial meditation practice might experience more pronounced reductions in depressive symptoms due to the acquisition of additional skills that could help manage symptoms.^40^ This could potentially distort the relationship between the intervention and the trajectory of depressive symptoms over time. Moreover, patients choosing to discontinue medication may show variations in their response to the intervention, possibly opting for discontinuation as a result of an improvement and feeling better.^41,42^ However, the discontinuation of medication might also lead to an elevation of depressive symptoms, which could be misinterpreted as a response to the assigned intervention.^43^ Therefore, we included direct paths from meditation practice to the mediator, from meditation practice to the outcome, and from ADM discontinuation to the outcome. A graphical representation of these potential confounding effects in the moderated-mediation model is provided in *Figure S5b*.

Figure S5*b*: *Moderated-mediation sensitivity analysis.*

X = independent variable (i.e., allocation group: maintenance antidepressant medication (m-ADM) vs mindfulness-based cognitive therapy (MBCT plus m-ADM discontinuation). M = mediator (i.e., pre-post (T0-T1) improvements in mindfulness skills). Y = dependent variable (i.e., the linear slope of the growth trajectory of depressive symptoms over time, from T0 to T5). W = moderator (i.e., latent profiles of patients demarcated by distinct depression severity). a = direct path between the independent variable and the mediator. b = direct path between the mediator and the outcome. c = total effects. c’ = direct effect of the independent variable on the dependent variable after adjustment for the mediating effects. w1 = interaction term between allocation group and latent profile in the relationship from allocation group to pre-post improvements in mindfulness skills. w2 = interaction term between allocation group and latent profile in the direct relationship from allocation group to the linear slope of the growth trajectory of depressive symptoms over time. w3 = interaction term between pre-post improvements in mindfulness skills and latent profile in the direct relationship from pre-post improvements in mindfulness skills to the linear slope of the growth trajectory of depressive symptoms over time. Meditation Practice = amount of formal meditation practice during last month measured at post-treatment (T1) as a potential confounder of the mediator and the outcome. ADM Discontinuation = discontinuation in antidepressant intake over the course of the study as a potential confounder of the outcome. p1 = direct path between meditation practice (confounder) and the mediator. p2 = direct path between meditation practice (confounder) and the outcome. d = direct path between ADM discontinuation (confounder) and the outcome. The interaction terms between the moderator and the covariates, pointing to potential differential covariate effects by LP, are considered in the analyses but are not included in the figure to facilitate understanding.

As it has been reported in a previous study, outcomes in the PREVENT trial did not cluster by therapists.^44^ Therefore, we proceeded without considering such clustering effects in our analyses. We used the ML estimation and provided unstandardized path estimates in the original scale of the corresponding dependent variable to facilitate interpretation. We calculated p-values for each path coefficient using the delta method, but we used the bootstrapped 95% confidence interval (95% CI) for the indirect effect using 10,000 samples (indirect effects are considered significant when their 95% CI does not include zero).^45^ We used the index of moderated-mediation (i.e., the difference in the indirect effects across levels of the moderator, which is significant when their 95% CI does not include zero) to test the significance of the moderated-mediation.^46^

Currently available effect size measures for mediation have important limitations. For instance, it has been suggested that certain measures, such as ratios comparing the indirect effect with the total effect, are highly dependent on the sample size.^47^ Other measures, like R^2^_mediation_ ,^44^ may yield negative values in the presence of suppression effects (i.e., reductions in prediction power as a result of considering a pair of variables when predicting variance in an outcome). Additionally, some measures lack clear boundaries, e.g., κ^2^ ,^48^ making interpretation challenging. Further research is needed to clearly establish the properties of these measures. Under this scenario, and exercising caution, we used the product of (a) the squared correlation of the mediator and the independent variable and (b) the squared partial correlation of the dependent variable and the mediator, adjusting for the independent variable. The product of these two terms represents the portion of variability in the dependent variable that is jointly explained by the independent variable and the mediator within the context of the mediation model, and therefore represents the proportion of variance accounted for by the indirect effect.^49^ To contextualize the magnitude of the effect, we introduced a scaling factor. To do so, we used the reciprocal of the total proportion of variance in the dependent variable accounted for by both the mediator and the independent variable. This scaling factor offers perspective on the effect's magnitude relative to the total proportion of variance in the dependent variable explained by all variables in the model.^50^ As a general reference, we used Cohen’s benchmarks for the interpretation of proportions of explained variance (small = 2%, medium = 15%, and large = 25%).^51^

**Supplement S6: Adherence to treatment by intervention group.**

|  | **m-ADM** | **MBCT-TS** |
| --- | --- | --- |
| **m-ADM adherence** |  |  |
| *Remained on therapeutic dose* | 162 (76.4) | 79 (37.3) |
| *Did not remain on therapeutic dose* | 50 (23.6) | 133 (62.7) |
|  |  |  |
| **Formal Meditation** |  |  |
| *Not at all* | 141 (66.5) | 27 (12.7) |
| *Sometimes* | 16 (7.5) | 54 (25.5) |
| *Regularly* | 3 (1.4) | 37 (17.5) |
| *More days than not* | 4 (1.9) | 49 (23.1) |
| *Missing* | 48 (22.6) | 45 (21.2) |
|  |  |  |
|  |  | **MBCT sessions** |
|  |  | *Completed less than 4 sessions* |
|  |  | 36 (17.0) |
|  |  | *Completed 4 or more sessions* |
|  |  | 176 (83.0) |

m-ADM: maintenance antidepressant medication. MBCT-TS: mindfulness-based cognitive therapy (tapering support). m-ADM adherence (ADM discontinuation in the models and analyses) refers to whether participants remained on a therapeutic stable dose of antidepressant medication in line with British National Formulary (BNF) and NICE guidance for the duration of the entire study. Formal meditation refers to the following question: “How much have you practised any formal meditation in last month?”. This question was introduced at the post-treatment timepoint (one month after the end of the MBCT training or the equivalent time in the m-ADM arm), and it was completed by 164 participants in the m-ADM group, and by 167 participants in the MBCT group. Numbers are frequencies (percentages). m-ADM use in patients who attended ≥4 sessions of MBCT-TS can be seen elsewhere.^44^

**Supplement S7: Self-reported antidepressant medication use over time by group**

Note: Frequencies (%) and mean (SD) of dosages, as well as details of cases taking a combination of two antidepressants (NA in the figure) can be seen in Table S7a.

*Table S7a. Types and doses of antidepressants used in the m-ADM group over time*

|  | **Baseline** | | **Post-treatment** | | **9-month follow-up** | | **12-month follow-up** | | **18-month follow-up** | | **24-month follow-up** | |
| --- | --- | --- | --- | --- | --- | --- | --- | --- | --- | --- | --- | --- |
|  | **N (%)** | **M (SD)** | **N (%)** | **M (SD)** | **N (%)** | **M (SD)** | **N (%)** | **M (SD)** | **M (SD)** | **M (SD)** | **N (%)** | **M (SD)** |
| *Amitriptyline* | 1 (0.5) | 100.0 (-) | 1 (0.5) | 100.0 (-) | 3 (1.4) | 66.7 (28.9) | 3 (1.4) | 66.7 (28.9) | 4 (1.9) | 52.5 (36.9) | 4 (1.9) | 52.5 (36.9) |
| *Citalopram* | 105 (49.5) | 27.1 (11.0) | 80 (37.7) | 27.9 (11.2) | 61 (28.8) | 25.9 (11.1) | 73 (34.4) | 25.3 (11.1) | 62 (29.2) | 24.8 (10.7) | 71 (33.5) | 26.1 (12.0) |
| *Citalopram+Amitriptyline* | 1 (0.5) | 40.0 (-) / 40.0 (-) | 0 (0) | - | 1 (0.5) | 30.0 (-) / 20.0 (-) | 0 (0) | - | 0 (0) | - | 0 (0) | - |
| *Citalopram+Dosulepin* | 1 (0.5) | 20.0 (-) / 50.0 (-) | 1 (0.5) | 20.0 (-) / 50.0 (-) | 1 (0.5) | 20.0 (-) / 25.0 (-) | 1 (0.5) | 20.0 (-) / 25.0 (-) | 1 (0.5) | 20.0 (-) / 25.0 (-) | 1 (0.5) | 20.0 (-) / 25.0 (-) |
| *Clomipramine* | 1 (0.5) | 250.0 (-) | 1 (0.5) | 250.0 (-) | 1 (0.5) | 250.0 (-) | 1 (0.5) | 250.0 (-) | 1 (0.5) | 250.0 (-) | 0 (0) | - |
| *Dosulepin* | 2 (0.9) | 112.5 (53.0) | 1 (0.5) | 75.0 (-) | 1 (0.5) | 75.0 (-) | 1 (0.5) | 75.0 (-) | 2 (0.9) | 112.5 (53.0) | 2 (0.9) | 92.9 (25.3) |
| *Duloxetine* | 1 (0.5) | 60.0 (-) | 0 (0) | - | 0 (0) | - | 1 (0.5) | 20.0 (-) | 2 (0.9) | 40.0 (28.3) | 0 (0) | - |
| *Escitalopram* | 4 (1.9) | 22.5 (12.6) | 3 (1.4) | 26.7 (11.6) | 3 (1.4) | 26.7 (11.6) | 2 (0.9) | 30.0 (14.1) | 2 (0.9) | 20.0 (0.0) | 3 (1.4) | 16.7 (5.8) |
| *Fluoxetine* | 40 (18.9) | 25.5 (10.1) | 38 (17.9) | 25.5 (10.1) | 26 (12.3) | 26.8 (12.5) | 28 (13.2) | 25.0 (11.7) | 30 (14.2) | 24.6 (9.5) | 28 (13.2) | 22.9 (6.6) |
| *Fluoxetine+Amitriptiline* | 0 (0) | - | 0 (0) | - | 1 (0.5) | 20.0 (-) / 30.0 (-) | 0 (0) | - | 0 (0) | - | 1 (0.5) | 60.0 (-) / 10.0 (-) |
| *Fluoxetine+Mirtazepine* | 0 (0) | - | 0 (0) | - | 1 (0.5) | - | 0 (0) | - | 0 (0) | - | 0 (0) | - |
| *Fluoxetine+Nortriptiline* | 1 (0.5) | 20.0 (-) / 10.0 (-) | 0 (0) | - | 0 (0) | - | 0 (0) | - | 0 (0) | - | 0 (0) | - |
| *Flupentixol* | 1 (0.5) | 35.0 (-) | 0 (0) | - | 0 (0) | - | 0 (0) | - | 0 (0) | - | 0 (0) | - |
| *Flupentixol+Mirtazepine* | 0 (0) | - | 0 (0) | - | 1 (0.5) | - | 1 (0.5) | - | 1 (0.5) | - | 1 (0.5) | - |
| *Lofepramine* | 4 (1.9) | 161.8 (43.5) | 4 (1.9) | 161.8 (43.5) | 3 (1.4) | 169.2 (50.5) | 3 (1.4) | 169.2 (50.5) | 2 (0.9) | 175.0 (49.5) | 3 (1.4) | 186.7 (40.4) |
| *Mirtazepine* | 9 (4.2) | 30.0 (7.5) | 7 (3.3) | 30.0 (8.7) | 7 (3.3) | 25.7 (7.3) | 7 (3.3) | 27.9 (5.7) | 6 (2.8) | 30.0 (0.0) | 6 (2.8) | 24.9 (7.9) |
| *Paroxetine* | 7 (3.3) | 23.3 (5.2) | 6 (2.8) | 23.3 (5.2) | 3 (1.4) | 26.7 (11.6) | 5 (2.4) | 27.1 (9.6) | 5 (2.4) | 26.0 (11.4) | 3 (1.4) | 20.0 (10.0) |
| *Sertraline* | 20 (9.4) | 92.1 (38.2) | 18 (8.5) | 86.1 (41.3) | 15 (7.1) | 83.3 (45.0) | 16 (7.5) | 100.0 (51.6) | 13 (6.1) | 96.2 (47.7) | 17 (8.0) | 91.2 (47.6) |
| *Agomelatine* | 1 (0.5) | 25.0 (-) | 0 (0) | - | 0 (0) | - | 0 (0) | - | 0 (0) | - | 0 (0) | - |
| *Venlafaxine* | 8 (3.8) | 134.4 (59.7) | 8 (3.8) | 103.1 (38.8) | 9 (4.2) | 108.3 (39.5) | 9 (4.2) | 120.8 (45.1) | 8 (3.8) | 139.1 (62.5) | 10 (4.7) | 115.3 (52.2) |
| *No medication* | 0 (0) | - | 2 (0.9) | - | 6 (2.8) | - | 8 (3.8) | - | 10 (4.7) | - | 14 (6.6) | - |

Missing values. Baseline: n = 5 (2.4%). Post-treatment: n = 42 (19.8%). 9-month follow-up: n = 70 (33.0%). 12-month follow-up: n = 53 (25.0%). 18-month follow-up: n = 63 (29.7%). 24-month follow-up: n = 48 (22.6%).

N (%) = frequency (percentage). M (SD) = mean (standard deviation), expressed in mgs.

Note: Frequencies (%) and mean (SD) of dosages, as well as details of cases taking a combination of two antidepressants (NA in the figure) can be seen in Table S7b.

*Table S7b. Types and doses of antidepressants used in the MBCT-TS group over time*

|  | **Baseline** | | **Post-treatment** | | **9-month follow-up** | | **12-month follow-up** | | **18-month follow-up** | | **24-month follow-up** | |
| --- | --- | --- | --- | --- | --- | --- | --- | --- | --- | --- | --- | --- |
|  | **N (%)** | **M (SD)** | **N (%)** | **M (SD)** | **N (%)** | **M (SD)** | **N (%)** | **M (SD)** | **M (SD)** | **M (SD)** | **N (%)** | **M (SD)** |
| *Amitriptyline* | 2 (0.9) | 125.0 (35.4) | 2 (0.9) | 125.0 (35.4) | 2 (0.9) | 125.0 (35.4) | 2 (0.9) | 125.0 (35.4) | 2 (0.9) | 100.0 (70.0) | 1 (0.5) | 50.0 (-) |
| *Citalopram* | 84 (39.6) | 27.6 (11.0) | 61 (28.8) | 22.8 (11.4) | 38 (17.9) | 22.8 (12.0) | 42 (19.8) | 26.0 (11.1) | 29 (13.7) | 29.6 (17.1) | 28 (13.2) | 24.3 (11.0) |
| *Clomipramine* | 1 (0.5) | 150.0 (-) | 0 (0) | - | 0 (0) | - | 0 (0) | - | 0 (0) | - | 1 (0.5) | 150.0 (-) |
| *Dosulepin* | 3 (1.4) | 91.7 (14.4) | 3 (1.4) | 58.3 (14.4) | 2 (0.9) | 37.5 (17.7) | 3 (1.4) | 91.7 (52.0) | 3 (1.4) | 66.7 (28.9) | 4 (1.9) | 81.3 (23.9) |
| *Doxepin* | 1 (0.5) | 50.0 (-) | 1(0.5) | 25.0 (-) | 0 (0) | - | 0 (0) | - | 0 (0) | - | 0 (0) | - |
| *Duloxetine* | 2 (0.9) | 60.0 (0.0) | 2 (0.9) | 45.0 (21.2) | 1(0.5) | 30.0 (-) | 1(0.5) | 30.0 (-) | 0(0) | - | 0 (0) | - |
| *Escitalopram* | 2 (0.9) | 15.0 (7.1) | 2 (0.9) | 8.6 (2.0) | 1 (0.5) | 10.0 (-) | 1 (0.5) | 10.0 (-) | 3 (1.4) | 15.0 (5.0) | 2 (0.9) | 12.5 (3.5) |
| *Fluoxetine* | 53 (25.0) | 28.4 (12.0) | 38 (17.9) | 24.7 (10.4) | 26 (12.3) | 22.2 (5.7) | 23 (10.8) | 22.9 (6.9) | 18 (8.5) | 22.9 (6.5) | 19 (9.0) | 25.3 (11.2) |
| *Fluvoxamine* | 1 (0.5) | 100 .0 (-) | 1 (0.5) | 50.0 (-) | 1 (0.5) | 50.0 (-) | 1 (0.5) | 50.0 (-) | 1 (0.5) | 50.0 (-) | 1 (0.5) | 50.0 (-) |
| *Lofepramine* | 3 (1.4) | 210.0 (0.0) | 2 (0.9) | 178.2 (45.0) | 1 (0.5) | 70.0 (-) | 1 (0.5) | 70.0 (-) | 1 (0.5) | 500.0 (-) | 5 (2.4) | 154.0 (58.6) |
| *Mirtazepine* | 11 (5.2) | 34.1 (7.0) | 7 (3.3) | 26.7 (5.6) | 7 (3.3) | 19.3 (10.4) | 6 (2.8) | 23.8 (13.8) | 5 (2.4) | 21.5 (15.8) | 7 (3.3) | 26.8 (14.9) |
| *Moclobemide* | 0 (0) | - | 0 (0) | - | 0 (0) | - | 0 (0) | - | 1 (0.5) | 150.0 (-) | 0 (0) | - |
| *Paroxetine* | 9 (4.2) | 23.8 (7.4) | 7 (3.3) | 24.3 (7.9) | 4 (1.9) | 27.5 (9.6) | 6 (2.8) | 26.7 (8.2) | 5 (2.4) | 25.6 (8.8) | 6 (2.8) | 23.3 (8.2) |
| *Sertraline* | 22 (10.4) | 77.3 (45.6) | 17 (8.0) | 81.0 (55.2) | 12 (5.7) | 91.7 (69.4) | 14 (6.6) | 72.6 (47.8) | 13 (6.1) | 74.6 (46.1) | 15 (7.1) | 84.7 (46.6) |
| *Sertraline+Mirtazepine* | 0 (0) | - | 0 (0) | - | 1 (0.5) | 100.0 (-) / 30.0 (-) | 1 (0.5) | 100.0 (-) / 30.0 (-) | 0 (0) | - | 2 (0.9) | 100.0 (0.0) / 30.0 (0.0) |
| *Trazodone* | 2 (0.9) | 300.0 (-) | 2 (0.9) | 250.0 (141.4) | 2 (0.9) | 100.0 (0.0) | 2 (0.9) | 50.0 (0.0) | 2 (0.9) | 50.0 (0.0) | 1 (0.5) | 50.0 (0.0) |
| *Venlafaxine* | 11 (5.2) | 139.8 (88.9) | 10 (4.7) | 110.8 (62.6) | 7 (3.3) | 112.5 (68.5) | 7 (3.3) | 139.3 (56.1) | 7 (3.3) | 133.9 (52.4) | 9 (4.2) | 133.3 (46.4) |
| *Venlafaxine+Mirtazepine* | 2 (0.9) | 150.0 (106.1) / 30.0 (-) | 2 (0.9) | 150.0 (106.1) / 30.0 (-) | 0 (0) | - | 1 0.5) | 30.0 (-) / 25.0 (-) | 1 (0.5) | 10.0 (-) / 30.0 (-) | 1 0.5) | 37.5 (-) / 30.0 (-) |
| *No medication* | 0 (0) | - | 17 (8.0) | - | 48 (22.6) | - | 56 (26.4) | - | 52 (24.5) | - | 59 (27.8) | - |

Missing values. Baseline: n = 3 (1.4%). Post-treatment: n = 38 (17.9%). 9-month follow-up: n = 59 (27.8%). 12-month follow-up: n = 45 (21.2%). 18-month follow-up: n = 69 (32.5%). 24-month follow-up: n = 51 (24.1%).

N (%) = frequency (percentage). M (SD) = mean (standard deviation), expressed in mgs.

**Supplement S8: Number of visits to the GP by group over the study period**

| Group | M | SD | Median | Q1 | Q3 | Minimum | Maximum |
| --- | --- | --- | --- | --- | --- | --- | --- |
|  |  |  |  |  |  |  |  |
| m-ADM | 14.9 | 9.2 | 15.5 | 8.8 | 14.5 | 1.0 | 64.0 |
| MBCT-TS | 14.6 | 7.9 | 14.0 | 8.5 | 14.0 | 0.0 | 35.0 |
|  |  |  |  |  |  |  |  |

Missing values: m-ADM (n=18), MBCT-TS (n=19). GP = General Practitioner.

Numbers include visits to the GP for any reason.

**Supplement S9: Selected participant baseline characteristics by post-treatment status.**

|  | **Patients lost to post-treatment*** | | | **Remaining patients**** | | |
| --- | --- | --- | --- | --- | --- | --- |
|  | **m-ADM (n = 38)** | **MBCT (n = 38)** | **Total** | **m-ADM (n = 174)** | **MBCT (n = 174)** | **Total** |
| Demographic characteristics |  |  |  |  |  |  |
| Age, M (SD) | 43.87 (12.00) | 42.71 (11.71) | 43.29 (11.79) | 49.77 (12.67) | 51.79 (11.28) | 50.78 (12.02) |
| Gender, *Female*, n (%) | 31 (81.6) | 28 (73.7) | 59 (77.6) | 143 (82.2) | 123 (70.7) | 266 (76.4) |
| Ethnicity, *White*, n (%) | 38 (100) | 38 (100) | 76 (100) | 172 (98.9) | 172 (98.9) | 344 (98.9) |
| Marital status^†^ |  |  |  |  |  |  |
| *Single, n (%)* | 12 (32.4) | 15 (40.5) | 27 (36.5) | 26 (14.9) | 27 (15.5) | 53 (15.3) |
| *Married, cohabiting, civil partnership,* n (%) | 19 (51.3) | 11 (29.7) | 30 (40.5) | 121 (69.5) | 114 (65.5) | 135 (67.5) |
| *Separated, divorced, widowed,* n (%) | 6 (16.3) | 11 (29.7) | 17 (23.0) | 27 (15.5) | 33 (19.0) | 60 (17.2) |
| Education^††^ |  |  |  |  |  |  |
| *No educational qualification,* n (%) | 1 (2.9) | 0 (0.0) | 1 (1.4) | 9 (5.2) | 10 (5.8) | 19 (5.5) |
| *O levels or GCSEs,* n (%) | 10 (28.6) | 6 (17.1) | 16 (22.9) | 35 (20.2) | 30 (17.4) | 65 (18.8) |
| *AS and A levels or vocational qualification,* n (%) | 14 (40.0) | 13 (37.1) | 27 (38.6) | 78 (45.1) | 71 (41.3) | 149 (43.3) |
| *University training,* n (%) | 10 (28.5) | 16 (45.8) | 26 (37.1) | 51 (29.5) | 61 (35.5) | 112 (32.4) |
| Employment status^†††^ |  |  |  |  |  |  |
| *Not working,* n (%) | 13 (35.1) | 21 (56.8) | 34 (46.0) | 69 (39.7) | 77 (44.5) | 146 (42.1) |
| *Part time,* n (%) | 13 (35.1) | 7 (18.9) | 20 (27.0) | 57 (32.8) | 46 (26.6) | 103 (29.7) |
| *Full time,* n (%) | 11 (29.8) | 9 (24.3) | 20 (27.0) | 48 (27.5) | 50 (28.9) | 98 (28.2) |
| Quality of life^††††^ |  |  |  |  |  |  |
| Quality of life rating (range: 1-5), M (SD) | 3.65 (1.04) | 3.54 (0.74) | 3.59 (0.90) | 3.73 (0.79) | 3.68 (0.81) | 3.70 (0.80) |
| Health satisfaction (range: 1-5), M (SD) | 2.91 (0.97) | 2.83 (0.95) | 2.87 (0.95) | 3.11 (0.99) | 2.94 (1.04) | 3.02 (1.02) |
| Physical (range: 4-20), M (SD) | 15.31 (10.72) | 15.71 (11.76) | 15.51 (11.18) | 14.23 (2.87) | 14.22 (4.84) | 14.23 (3.98) |
| Psychological (range: 4-20), M (SD) | 11.94 (3.22) | 12.55 (2.49) | 12.25 (2.87) | 12.40 (2.45) | 12.62 (2.66) | 12.51 (2.55) |
| Social (range: 4-20), M (SD) | 12.28 (4.24) | 13.37 (3.23) | 12.83 (3.77) | 13.29 (3.22) | 13.40 (3.46) | 13.34 (3.34) |
| Environment (range: 4-20), M (SD) | 13.75 (3.12) | 14.63 (2.26) | 14.19 (2.73) | 15.34 (2.36) | 15.11 (2.41) | 15.22 (2.39) |
| Mindfulness skills^†††††^ |  |  |  |  |  |  |
| FFMQ (range: 39-195), M (SD) | 117.44 (20.44) | 117.88 (13.58) | 117.66 (17.17) | 118.03 (16.62) | 119.52 (19.53) | 118.78 (18.14) |
| Depressive symptoms^††††††^ |  |  |  |  |  |  |
| BDI-II (range: 0-63), M (SD) | 15.76 (12.76) | 12.78 (9.62) | 14.23 (11.27) | 14.19 (9.47) | 13.97 (10.34) | 14.08 (9.90) |

* Defined as those participants with missing data on BDI-II at post-treatment. ** Defined as those participants with no missing data on BDI-II at post-treatment.

^†^ Sample size in lost to post-treatment = 74: m-ADM: 37; MBCT: 37. Sample size in remaining participant’s group = 348: m-ADM: 174; MBCT: 174.

^††^ Sample size in lost to post-treatment = 70: m-ADM: 35; MBCT: 35. Sample size in remaining participant’s group = 345: m-ADM: 173; MBCT: 172.

^†††^ Sample size in lost to post-treatment = 74: m-ADM: 37; MBCT: 37. Sample size in remaining participant’s group = 347: m-ADM: 174; MBCT: 173.

^††††^ Sample size in lost to post-treatment = 69: m-ADM: 34; MBCT: 35. Sample size in remaining participant’s group = 345: m-ADM: 171; MBCT: 174.

^†††††^ Sample size in lost to post-treatment = 65: m-ADM: 32; MBCT: 33. Sample size in remaining participant’s group = 344: m-ADM: 170; MBCT: 174.

^††††††^ Sample size in lost to post-treatment = 70: m-ADM: 34; MBCT: 36. Sample size in remaining participant’s group = 346: m-ADM: 172; MBCT: 174.

**Supplement S10: Selected participant baseline characteristics by 9-month follow-up status.**

|  | **Patients lost to post-treatment*** | | | **Remaining patients**** | | |
| --- | --- | --- | --- | --- | --- | --- |
|  | **m-ADM (n = 70)** | **MBCT (n = 61)** | **Total** | **m-ADM (n = 142)** | **MBCT (n = 151)** | **Total** |
| Demographic characteristics |  |  |  |  |  |  |
| Age, M (SD) | 45.61 (12.52) | 44.52 (11.67) | 45.11 (12.10) | 50.24 (12.60) | 52.44 (11.18) | 51.37 (11.92) |
| Gender, *Female*, n (%) | 58 (82.9) | 45 (73.8) | 103 (78.6) | 116 (81.7) | 106 (70.2) | 222 (75.8) |
| Ethnicity, *White*, n (%) | 69 (98.6) | 61 (100) | 130 (99.2) | 141 (99.3) | 149 (98.7) | 290 (99.0) |
| Marital status^†^ |  |  |  |  |  |  |
| *Single, n (%)* | 18 (26.2) | 23 (38.3) | 41 (31.8) | 20 (14.1) | 19 (12.6) | 39 (13.3) |
| *Married, cohabiting, civil partnership,* n (%) | 41 (59.4) | 26 (43.3) | 67 (51.9) | 99 (69.7) | 99 (65.6) | 198 (67.5) |
| *Separated, divorced, widowed,* n (%) | 10 (14.4) | 11 (18.4) | 21 (16.3) | 23 (16.2) | 33 (21.8) | 56 (19.2) |
| Education^††^ |  |  |  |  |  |  |
| *No educational qualification,* n (%) | 1 (1.5) | 3 (5.2) | 4 (3.2) | 9 (6.4) | 7 (4.8) | 16 (5.5) |
| *O levels or GCSEs,* n (%) | 19 (28.3) | 10 (17.3) | 29 (23.2) | 26 (18.4) | 26 (17.4) | 52 (17.9) |
| *AS and A levels or vocational qualification,* n (%) | 29 (43.3) | 23 (39.6) | 52 (41.6) | 63 (44.7) | 61 (41.0) | 124 (42.8) |
| *University training,* n (%) | 18 (26.9) | 22 (37.9) | 40 (32.0) | 43 (30.5) | 55 (36.8) | 98 (33.8) |
| Employment status^†††^ |  |  |  |  |  |  |
| *Not working,* n (%) | 25 (36.2) | 31 (51.7) | 56 (43.4) | 57 (40.1) | 67 (44.7) | 124 (42.5) |
| *Part time,* n (%) | 22 (31.9) | 14 (23.3) | 36 (27.9) | 48 (33.8) | 39 (26.0) | 87 (29.8) |
| *Full time,* n (%) | 22 (31.9) | 15 (25.0) | 37 (28.7) | 37 (26.1) | 44 (29.3) | 81 (27.7) |
| Quality of life^††††^ |  |  |  |  |  |  |
| Quality of life rating (range: 1-5), M (SD) | 3.55 (0.98) | 3.47 (0.84) | 3.51 (0.91) | 3.79 (0.75) | 3.73 (0.77) | 3.76 (0.76) |
| Health satisfaction (range: 1-5), M (SD) | 2.95 (1.00) | 2.83 (1.06) | 2.89 (1.03) | 3.13 (0.98) | 2.95 (1.02) | 3.04 (1.00) |
| Physical (range: 4-20), M (SD) | 14.67 (7.92) | 14.67 (9.43) | 14.67 (8.64) | 14.30 (3.00) | 14.40 (5.00) | 14.35 (4.15) |
| Psychological (range: 4-20), M (SD) | 11.81 (2.87) | 12.40 (2.52) | 12.09 (2.72) | 12.55 (2.42) | 12.68 (2.67) | 12.62 (2.55) |
| Social (range: 4-20), M (SD) | 12.00 (3.65) | 13.09 (2.99) | 12.52 (3.38) | 13.63 (3.20) | 13.51 (3.56) | 13.57 (3.39) |
| Environment (range: 4-20), M (SD) | 14.13 (2.87) | 14.54 (2.26) | 14.32 (2.59) | 15.51 (2.30) | 15.21 (2.41) | 15.36 (2.36) |
| Mindfulness skills^†††††^ |  |  |  |  |  |  |
| FFMQ (range: 39-195), M (SD) | 114.10 (18.06) | 117.02 (14.75) | 115.50 (16.56) | 119.60 (16.64) | 120.09 (19.94) | 119.85 (18.39) |
| Depressive symptoms^††††††^ |  |  |  |  |  |  |
| BDI-II (range: 0-63), M (SD) | 16.54 (11.25) | 13.95 (9.52) | 15.31 (10.50) | 13.48 (9.36) | 13.70 (10.49) | 13.59 (9.94) |

* Defined as those participants with missing data on BDI-II at 9-month follow-up. ** Defined as those participants with no missing data on BDI-II at 9-month follow-up.

^†^ Sample size in lost to post-treatment = 129: m-ADM: 69; MBCT: 60. Sample size in remaining participant’s group = 293: m-ADM: 142; MBCT: 151.

^††^ Sample size in lost to post-treatment = 125: m-ADM: 67; MBCT: 58. Sample size in remaining participant’s group = 290: m-ADM: 141; MBCT: 149.

^†††^ Sample size in lost to post-treatment = 129: m-ADM: 69; MBCT: 60. Sample size in remaining participant’s group = 292: m-ADM: 142; MBCT: 150.

^††††^ Sample size in lost to post-treatment = 122: m-ADM: 64; MBCT: 58. Sample size in remaining participant’s group = 292: m-ADM: 141; MBCT: 151.

^†††††^ Sample size in lost to post-treatment = 117: m-ADM: 61; MBCT: 56. Sample size in remaining participant’s group = 292: m-ADM: 141; MBCT: 151.

^††††††^ Sample size in lost to post-treatment = 124: m-ADM: 65; MBCT: 59. Sample size in remaining participant’s group = 292: m-ADM: 141; MBCT: 151.

**Supplement S11: Selected participant baseline characteristics by 12-month follow-up status.**

|  | **Patients lost to post-treatment*** | | | **Remaining patients**** | | |
| --- | --- | --- | --- | --- | --- | --- |
|  | **m-ADM (n = 55)** | **MBCT (n = 45)** | **Total** | **m-ADM (n = 157)** | **MBCT (n = 167)** | **Total** |
| Demographic characteristics |  |  |  |  |  |  |
| Age, M (SD) | 46.11 (11.95) | 43.33 (12.87) | 44.86 (12.39) | 49.62 (12.91) | 52.00 (10.89) | 50.85 (11.95) |
| Gender, *Female*, n (%) | 49 (61.3) | 31 (38.8) | 80 (24.6) | 125 (51.0) | 120 (49.0) | 245 (75.4) |
| Ethnicity, *White*, n (%) | 54 (98.2) | 45 (100) | 99 (99.0) | 156 (99.4) | 165 (98.8) | 321 (99.1) |
| Marital status^†^ |  |  |  |  |  |  |
| *Single, n (%)* | 16 (29.6) | 15 (34.1) | 31 (31.6) | 22 (14.0) | 27 (16.1) | 49 (15.1) |
| *Married, cohabiting, civil partnership,* n (%) | 30 (55.5) | 18 (40.9) | 48 (49.0) | 110 (70.0) | 107 (64.1) | 217 (67.0) |
| *Separated, divorced, widowed,* n (%) | 8 (14.9) | 11 (25.0) | 19 (19.4) | 25 (16.0) | 33 (19.8) | 58 (17.9) |
| Education^††^ |  |  |  |  |  |  |
| *No educational qualification,* n (%) | 0 (0.0) | 1 (2.4) | 1 (1.1) | 10 (6.5) | 9 (5.5) | 19 (5.9) |
| *O levels or GCSEs,* n (%) | 17 (32.0) | 8 (19.0) | 25 (26.3) | 28 (18.1) | 28 (17.0) | 56 (17.5) |
| *AS and A levels or vocational qualification,* n (%) | 19 (35.8) | 17 (40.5) | 36 (37.9) | 73 (47.0) | 67 (40.6) | 140 (43.8) |
| *University training,* n (%) | 17 (32.2) | 16 (38.1) | 33 (34.7) | 44 (28.4) | 61 (36.9) | 105 (32.8) |
| Employment status^†††^ |  |  |  |  |  |  |
| *Not working,* n (%) | 20 (37.1) | 25 (56.8) | 45 (45.9) | 62 (39.5) | 73 (44.0) | 135 (41.8) |
| *Part time,* n (%) | 18 (33.3) | 4 (9.1) | 22 (22.5) | 52 (33.1) | 49 (29.5) | 101 (31.3) |
| *Full time,* n (%) | 16 (29.6) | 15 (34.1) | 31 (31.6) | 43 (27.4) | 44 (26.5) | 97 (26.9) |
| Quality of life^††††^ |  |  |  |  |  |  |
| Quality of life rating (range: 1-5), M (SD) | 3.59 (1.00) | 3.58 (0.82) | 3.59 (0.92) | 3.76 (0.77) | 3.67 (0.80) | 3.71 (0.79) |
| Health satisfaction (range: 1-5), M (SD) | 2.92 (1.04) | 2.79 (1.17) | 2.86 (1.10) | 3.12 (0.97) | 2.95 (0.99) | 3.03 (0.98) |
| Physical (range: 4-20), M (SD) | 14.52 (8.94) | 15.06 (10.77) | 14.77 (9.79) | 14.38 (2.99) | 14.32 (4.88) | 14.35 (4.07) |
| Psychological (range: 4-20), M (SD) | 11.54 (2.65) | 12.36 (2.68) | 11.92 (2.68) | 12.57 (2.53) | 12.67 (2.61) | 12.62 (2.57) |
| Social (range: 4-20), M (SD) | 11.65 (3.46) | 12.68 (3.17) | 12.13 (3.35) | 13.59 (3.29) | 13.57 (3.46) | 13.58 (3.37) |
| Environment (range: 4-20), M (SD) | 14.17 (2.72) | 14.71 (2.58) | 14.42 (2.66) | 15.36 (2.45) | 15.11 (2.34) | 15.23 (2.39) |
| Mindfulness skills^†††††^ |  |  |  |  |  |  |
| FFMQ (range: 39-195), M (SD) | 111.98 (17.88) | 114.76 (17.25) | 113.27 (17.54) | 119.74 (16.66) | 120.37 (18.91) | 120.07 (17.84) |
| Depressive symptoms^††††††^ |  |  |  |  |  |  |
| BDI-II (range: 0-63), M (SD) | 18.02 (10.47) | 14.80 (10.71) | 16.51 (10.65) | 13.30 (9.69) | 13.49 (10.08) | 13.40 (9.88) |

* Defined as those participants with missing data on BDI-II at 12-month follow-up. ** Defined as those participants with no missing data on BDI-II at 12-month follow-up.

^†^ Sample size in lost to post-treatment = 98: m-ADM: 54; MBCT: 44. Sample size in remaining participant’s group = 324: m-ADM: 157; MBCT: 167.

^††^ Sample size in lost to post-treatment = 95: m-ADM: 53; MBCT: 42. Sample size in remaining participant’s group = 320: m-ADM: 155; MBCT: 165.

^†††^ Sample size in lost to post-treatment = 98: m-ADM: 54; MBCT: 44. Sample size in remaining participant’s group = 323: m-ADM: 157; MBCT: 166.

^††††^ Sample size in lost to post-treatment = 92: m-ADM: 49; MBCT: 43 Sample size in remaining participant’s group = 322: m-ADM: 156; MBCT: 166.

^†††††^ Sample size in lost to post-treatment = 88: m-ADM: 47; MBCT: 41. Sample size in remaining participant’s group = 321: m-ADM: 155; MBCT: 166.

^††††††^ Sample size in lost to post-treatment = 94: m-ADM: 50; MBCT: 44. Sample size in remaining participant’s group = 322: m-ADM: 156; MBCT: 166.

**Supplement S12: Selected participant baseline characteristics by 18-month follow-up status.**

|  | **Patients lost to post-treatment*** | | | **Remaining patients**** | | |
| --- | --- | --- | --- | --- | --- | --- |
|  | **m-ADM (n = 63)** | **MBCT (n = 70)** | **Total** | **m-ADM (n = 149)** | **MBCT (n = 142)** | **Total** |
| Demographic characteristics |  |  |  |  |  |  |
| Age, M (SD) | 45.06 (11.83) | 47.19 (13.19) | 46.18 (12.56) | 50.26 (12.82) | 51.63 (10.89) | 50.92 (11.92) |
| Gender, *Female*, n (%) | 51 (81.0) | 51 (72.9) | 102 (76.7) | 123 (82.6) | 100 (70.4) | 223 (76.6) |
| Ethnicity, *White*, n (%) | 63 (100) | 68 (97.1) | 131 (98.5) | 147 (98.7) | 142 (100) | 289 (99.3) |
| Marital status^†^ |  |  |  |  |  |  |
| *Single, n (%)* | 20 (32.3) | 19 (27.6) | 39 (29.8) | 18 (12.1) | 23 (16.2) | 41 (14.2) |
| *Married, cohabiting, civil partnership,* n (%) | 39 (51.6) | 48 (44.9) | 87 (48.1) | 108 (72.5) | 94 (66.2) | 202 (69.3) |
| *Separated, divorced, widowed,* n (%) | 10 (16.1) | 19 (27.5) | 29 (22.1) | 23 (15.4) | 25 (17.6) | 48 (16.5) |
| Education^††^ |  |  |  |  |  |  |
| *No educational qualification,* n (%) | 1 (1.7) | 1 (1.5) | 2 (1.6) | 9 (6.1) | 9 (6.4) | 18 (6.2) |
| *O levels or GCSEs,* n (%) | 12 (20.0) | 10 (15.2) | 22 (17.5) | 33 (22.2) | 26 (18.4) | 59 (20.4) |
| *AS and A levels or vocational qualification,* n (%) | 30 (49.9) | 27 (40.9) | 57 (45.3) | 62 (41.9) | 57 (40.4) | 119 (41.2) |
| *University training,* n (%) | 17 (28.4) | 28 (42.4) | 45 (35.6) | 44 (29.8) | 49 (34.8) | 93 (32.2) |
| Employment status^†††^ |  |  |  |  |  |  |
| *Not working,* n (%) | 22 (35.5) | 36 (52.9) | 58 (44.6) | 60 (40.3) | 62 (43.6) | 122 (42.0) |
| *Part time,* n (%) | 18 (29.0) | 10 (14.7) | 28 (21.6) | 52 (34.9) | 43 (30.3) | 95 (32.6) |
| *Full time,* n (%) | 22 (35.5) | 22 (32.4) | 44 (33.8) | 37 (24.8) | 37 (26.1) | 74 (25.4) |
| Quality of life^††††^ |  |  |  |  |  |  |
| Quality of life rating (range: 1-5), M (SD) | 3.48 (0.89) | 3.54 (0.87) | 3.51 (0.88) | 3.82 (0.79) | 3.71 (0.76) | 3.76 (0.78) |
| Health satisfaction (range: 1-5), M (SD) | 2.97 (1.02) | 2.81 (1.00) | 2.88 (1.01) | 3.12 (0.97) | 2.97 (1.04) | 3.05 (1.01) |
| Physical (range: 4-20), M (SD) | 14.30 (8.17) | 15.37 (10.60) | 14.86 (9.51) | 14.46 (2.92) | 14.04 (2.94) | 14.25 (2.93) |
| Psychological (range: 4-20), M (SD) | 11.83 (2.89) | 12.31 (2.69) | 12.08 (2.78) | 12.53 (2.43) | 12.75 (2.59) | 12.64 (2.51) |
| Social (range: 4-20), M (SD) | 11.79 (3.37) | 12.92 (3.19) | 12.39 (3.31) | 13.69 (3.30) | 13.62 (3.50) | 13.65 (3.39) |
| Environment (range: 4-20), M (SD) | 13.86 (2.55) | 14.76 (2.37) | 14.33 (2.49) | 15.59 (2.40) | 15.16 (2.39) | 15.38 (2.40) |
| Mindfulness skills^†††††^ |  |  |  |  |  |  |
| FFMQ (range: 39-195), M (SD) | 114.05 (17.30) | 116.83 (17.68) | 115.52 (17.49) | 119.54 (16.99) | 120.40 (19.10) | 119.96 (18.04) |
| Depressive symptoms^††††††^ |  |  |  |  |  |  |
| BDI-II (range: 0-63), M (SD) | 16.41 (11.19) | 14.87 (10.75) | 15.59 (10.95) | 13.62 (9.47) | 13.23 (9.92) | 13.43 (9.68) |

* Defined as those participants with missing data on BDI-II at 18-month follow-up. ** Defined as those participants with no missing data on BDI-II at 18-month follow-up.

^†^ Sample size in lost to post-treatment = 131: m-ADM: 62; MBCT: 69. Sample size in remaining participant’s group = 291: m-ADM: 149; MBCT: 142.

^††^ Sample size in lost to post-treatment = 126: m-ADM: 60; MBCT: 66. Sample size in remaining participant’s group = 289: m-ADM: 148; MBCT: 141.

^†††^ Sample size in lost to post-treatment = 130: m-ADM: 62; MBCT: 68. Sample size in remaining participant’s group = 291: m-ADM: 149; MBCT: 142.

^††††^ Sample size in lost to post-treatment = 129: m-ADM: 61; MBCT: 68. Sample size in remaining participant’s group = 285: m-ADM: 144; MBCT: 141.

^†††††^ Sample size in lost to post-treatment = 125: m-ADM: 59; MBCT: 66. Sample size in remaining participant’s group = 284: m-ADM: 143; MBCT: 141.

^††††††^ Sample size in lost to post-treatment = 130: m-ADM: 61; MBCT: 69. Sample size in remaining participant’s group = 286: m-ADM: 145; MBCT: 141.

**Supplement S13: Selected participant baseline characteristics by 24-month follow-up status.**

|  | **Patients lost to post-treatment*** | | | **Remaining patients**** | | |
| --- | --- | --- | --- | --- | --- | --- |
|  | **m-ADM (n = 45)** | **MBCT (n = 43)** | **Total** | **m-ADM (n = 167)** | **MBCT (n = 169)** | **Total** |
| Demographic characteristics |  |  |  |  |  |  |
| Age, M (SD) | 45.69 (12.47) | 44.95 (14.70) | 45.33 (13.53) | 49.53 (12.71) | 51.49 (10.66) | 50.51 (11.75) |
| Gender, *Female*, n (%) | 37 (82.2) | 33 (76.7) | 70 (79.5) | 137 (82.0) | 118 (69.8) | 255 (75.9) |
| Ethnicity, *White*, n (%) | 45 (100) | 43 (100) | 88 (100) | 165(98.8) | 167 (98.8) | 332 (98.8) |
| Marital status^†^ |  |  |  |  |  |  |
| *Single, n (%)* | 10 (22.7) | 16 (38.0) | 26 (30.3) | 28 (16.8) | 26 (15.4) | 54 (16.1) |
| *Married, cohabiting, civil partnership,* n (%) | 26 (59.1) | 17 (40.5) | 43 (50.0) | 114 (68.2) | 108 (63.9) | 222 (66.0) |
| *Separated, divorced, widowed,* n (%) | 8 (18.2) | 9 (21.5) | 17 (19.7) | 25 (15.0) | 35 (20.7) | 60 (17.9) |
| Education^††^ |  |  |  |  |  |  |
| *No educational qualification,* n (%) | 1 (2.3) | 1 (2.4) | 2 (2.4) | 9 (5.5) | 9 (5.4) | 18 (5.4) |
| *O levels or GCSEs,* n (%) | 11 (25.6) | 7 (17.9) | 18 (22.0) | 34 (20.5) | 29 (17.3) | 63 (18.9) |
| *AS and A levels or vocational qualification,* n (%) | 20 (46.5) | 12 (30.9) | 32 (39.0) | 72 (43.6) | 72 (42.8) | 144 (43.2) |
| *University training,* n (%) | 11 (25.6) | 19 (48.8) | 30 (36.6) | 50 (30.4) | 58 (34.5) | 108 (32.5) |
| Employment status^†††^ |  |  |  |  |  |  |
| *Not working,* n (%) | 17 (38.6) | 26 (61.9) | 43 (50.0) | 65 (39.0) | 72 (42.8) | 137 (40.9) |
| *Part time,* n (%) | 14 (31.8) | 5 (11.9) | 19 (22.1) | 56 (33.5) | 48 (28.6) | 104 (31.0) |
| *Full time,* n (%) | 13 (29.5) | 11 (26.2) | 24 (27.9) | 46 (27.5) | 48 (28.6) | 94 (28.1) |
| Quality of life^††††^ |  |  |  |  |  |  |
| Quality of life rating (range: 1-5), M (SD) | 3.60 (0.99) | 3.59 (0.84) | 3.59 (0.91) | 3.75 (0.79) | 3.67 (0.79) | 3.71 (0.79) |
| Health satisfaction (range: 1-5), M (SD) | 2.95 (1.10) | 2.66 (1.04) | 2.81 (1.08) | 3.10 (0.95) | 2.98 (1.02) | 3.04 (0.99) |
| Physical (range: 4-20), M (SD) | 14.76 (9.67) | 14.93 (11.04) | 14.84 (10.30) | 14.32 (2.93) | 14.36 (4.86) | 14.34 (4.02) |
| Psychological (range: 4-20), M (SD) | 12.22 (3.01) | 11.98 (2.58) | 12.10 (2.79) | 12.35 (2.47) | 12.76 (2.62) | 12.56 (2.55) |
| Social (range: 4-20), M (SD) | 12.75 (3.34) | 12.47 (2.75) | 12.61 (3.05) | 13.22 (3.45) | 13.62 (3.52) | 13.42 (3.49) |
| Environment (range: 4-20), M (SD) | 14.15 (2.49) | 14.90 (2.40) | 14.52 (2.46) | 15.32 (2.53) | 15.06 (2.39) | 15.19 (2.46) |
| Mindfulness skills^†††††^ |  |  |  |  |  |  |
| FFMQ (range: 39-195), M (SD) | 116.34 (18.12) | 114.33 (15.85) | 115.36 (16.97) | 118.34 (17.02) | 120.40 (19.15) | 119.40 (18.14) |
| Depressive symptoms^††††††^ |  |  |  |  |  |  |
| BDI-II (range: 0-63), M (SD) | 13.88 (9.69) | 15.48 (10.45) | 14.68 (10.05) | 14.59 (10.18) | 13.34 (10.13) | 13.96 (10.16) |

* Defined as those participants with missing data on BDI-II at 24-month follow-up. ** Defined as those participants with no missing data on BDI-II at 24-month follow-up.

^†^ Sample size in lost to post-treatment = 86: m-ADM: 44; MBCT: 42. Sample size in remaining participant’s group = 336: m-ADM: 167; MBCT: 169.

^††^ Sample size in lost to post-treatment = 82: m-ADM: 43; MBCT: 39. Sample size in remaining participant’s group = 333: m-ADM: 165; MBCT: 168.

^†††^ Sample size in lost to post-treatment = 86: m-ADM: 44; MBCT: 42. Sample size in remaining participant’s group = 335: m-ADM: 167; MBCT: 168.

^††††^ Sample size in lost to post-treatment = 83: m-ADM: 42; MBCT: 41. Sample size in remaining participant’s group = 331: m-ADM: 163; MBCT: 168.

^†††††^ Sample size in lost to post-treatment = 80: m-ADM: 41; MBCT: 39. Sample size in remaining participant’s group = 329: m-ADM: 161; MBCT: 168.

^††††††^ Sample size in lost to post-treatment = 84: m-ADM: 42; MBCT: 42. Sample size in remaining participant’s group = 332: m-ADM: 164; MBCT: 168.

**Supplement S14: Details for the latent profile analyses results.**

A random 50% split of the total sample resulted in two sub-samples of n_1_ = 212 participants (test sample) and n_2_ = 212 participants (validation sample). The baseline sociodemographic, quality of life, and depressive symptomatology variables as well as descriptive data for the predictors used to develop the LPA can be found by sub-sample in *Tables S14a* and *S14b*. There were no marked differences between treatment groups in terms of the predictors used in the LP analysis (*Table S14c*). At baseline, the mean HAMD scores of participants in both the test and validation samples suggested they were remitters with mild residual symptoms.^9^

We used LP analysis to identify baseline latent profiles (i.e., the moderator), and compared models with 1 through 5 profiles in the test sample first. All models were well-identified. As shown in *Table S14d*, having more LPs resulted in lower values for the information criteria, suggesting better model fit. However, the LMR-LRT identified no more than one profile, while the BLRT test suggested that more profiles are better. Entropy values ranged from 0.72 to 0.89, indicating that the LPs were well-separated, with a moderate to high level of accuracy in classifying individuals across LPs. Finally, the Elbow Plot (*Graph S14e*) suggested the steepest slope with only two profiles.

Given all this information, we compared the two- and three-profile models for conceptual interpretability and clarity. Consistent with the two-profile model, the three-profile model replicated the LP that was characterised by a more severe clinical history of depression. However, in the three-profile model, the largest profile was split into two less severe profiles. Confirming our expected two-profile solution, whilst considering the fit and parsimony, as well as the statistical power for subsequent analyses (the additional third profile was too small (less than 10%) to contribute to subsequent analyses), we chose the two-profile model. To validate the structure of the selected two-profile model, we repeated the process with the validation sample (*Table S14d*), in which all defining characteristics of the latent profiles were replicated, supporting the validity of our two-profile model. Following confirmation of a two-profile model structure from the two independent split-half samples, the dataset was recombined, and the same method of LP analysis was applied on the full sample (*Table S14e*). Then, we estimated the LP measurement model, generating BCH weights that reflect individual profile membership and the measurement error of the latent profile variable.

The largest subgroup of patients (LP1, 60.6%) was characterized by lower levels of adversity, longevity, and entrenchment of depression (i.e., lower depression severity). As shown in *Tables S14f-g*, this subgroup had lower scores of residual depressive symptoms, with a mean HAMD value approximately at the cut-off that distinguishes partial remitters from full remitters.^9^ While this subgroup had a slightly longer duration of the previous depressive episode, it nonetheless exhibited a lower prevalence of predictive factors such as a history of abuse, suicide attempts, previous depressive episodes, and a lower number of comorbidities. Additionally, it demonstrated a later onset of the first depression episode and lower severity of the last episode, along with reduced levels of negative and unresolved rumination, self-blame, lack of acceptance, and stigmatization. Furthermore, the LP1 subgroup displayed a greater ability to recognize early warning signs of depression, as well as higher levels of awareness, self-efficacy, positive affect, and social satisfaction. Although LP1 was not different from LP2 in terms of any sociodemographic data, it exhibited better quality of life indices in all domains and lower levels of depressive symptoms according to the BDI-II, with a mean value falling within the range of minimal depression (*Tables S14h and S14i*).

The other subgroup of patients (LP2, 39.4%) was characterized by higher levels of adversity, longevity, and entrenchment of depression (i.e., higher depression severity). As shown in *Tables S14f-g*, this subgroup had higher values of residual depressive symptoms, with a HAMD mean value close to the cut-off that distinguishes partial remitters from non-remitters.^9^ This subgroup had a shorter duration of the previous depressive episode but showed a higher prevalence of predictive factors, such as a history of abuse, suicide attempts, previous depressive episodes, and a higher number of comorbidities. Additionally, this subgroup reported an earlier onset of the first depressive episode, higher severity of the last episode, along with elevated levels of negative and unresolved rumination, self-blame, lack of acceptance, and stigmatization. Furthermore, this subgroup displayed a poorer ability to recognize early warning signs of depression, as well as lower awareness, self-efficacy, positive affect, and social satisfaction. LP2 did not differ from LPI in terms of any sociodemographic data but exhibited lower quality of life indices, and a higher level of depressive symptoms according to the BDI-II, with a mean value falling approximately at the cut-off that distinguishes moderate from mild depression (*Tables S14h and S14i*).

The two-profile model used to assign patients into LPs was characterized by high posterior probabilities (>0.9) for all LPs across both the randomly selected subsamples and the total sample, suggesting low classification error (*Table S14j*). The LP variable was used to estimate the subsequent auxiliary model (i.e., moderated-mediation), conditional on the LPs. Subsequent analyses were developed using the total sample.

Table S14*a***:** *Baseline sociodemographic in the test and validation samples.*

|  | **Test (n = 212)** | **Validation (n = 212)** |
| --- | --- | --- |
| Demographic characteristics |  |  |
| Age, M (SD) | 48.84 (12.41) | 50.03 (12.20) |
| Gender, *Women*, n (%) | 171 (80.7) | 154 (72.6) |
| Ethnicity, *White*, n (%) | 211 (99.5) | 209 (98.6) |
| Marital status |  |  |
| *Single, n (%)* | 50 (23.7) | 30 (14.2) |
| *Married, cohabiting, civil partnership,* n (%) | 123 (58.3) | 142 (67.3) |
| *Separated, divorced, widowed,* n (%) | 38 (18.0) | 39 (18.5) |
| Education |  |  |
| *No educational qualification,* n (%) | 9 (4.3) | 11 (5.3) |
| *O levels or GCSEs,* n (%) | 41 (19.7) | 40 (19.3) |
| *AS and A levels or vocational qualification,* n (%) | 19 (9.1) | 23 (11.1) |
| *University training,* n (%) | 72 (34.6) | 77 (31.8) |
| Employment status |  |  |
| *Not working,* n (%) | 79 (37.6) | 101 (47.9) |
| *Part time,* n (%) | 71 (33.8) | 52 (24.6) |
| *Full time,* n (%) | 60 (28.6) | 58 (27.5) |
| Quality of life |  |  |
| Quality of life rating (range: 1-5), M (SD) | 3.68 (0.81) | 3.69 (0.82) |
| Health satisfaction (range: 1-5), M (SD) | 2.98 (0.94) | 3.01 (1.07) |
| Physical (range: 4-20), M (SD) | 14.53 (4.41) | 14.36 (6.99) |
| Psychological (range: 4-20), M (SD) | 12.43 (2.52) | 12.50 (2.70) |
| Social (range: 4-20), M (SD) | 13.07 (3.34) | 13.45 (3.48) |
| Environment (range: 4-20), M (SD) | 15.02 (2.57) | 15.09 (2.38) |
| Mindfulness skills |  |  |
| FFMQ (range: 39-195), M (SD) | 117.66 (18.07) | 119.55 (18.87) |
| Depressive symptoms |  |  |
| BDI-II (range: 0-63), M (SD) | 13.98 (9.80) | 14.23 (10.48) |

GCSE=general certificate of secondary education. BDI-II=Beck Depression Inventory-II. FFMQ = Five Facets of Mindfulness Questionnaire. In the Test sample, 1 participant did not provide data on marital status, 4 participants did not provide data on education, 2 participants did not provide data on employment status, 4 participants did not provide data on any quality-of-life measure, 8 participants did not provide data on FFMQ, and 3 participants did not provide data on BDI-II. In the Validation sample, 1 participant did not provide data on marital status, 5 participants did not provide data on education, 1 participant did not provide data on employment status, 6 participants did not provide data on any quality-of-life measure, 7 participants did not provide data on FFMQ, and 5 participants did not provide data on BDI-II.

Table S14*b***:** *Baseline predictors of LPs in the test and validation samples.*

|  | **Test (n = 212)** | **Validation (n = 212)** |
| --- | --- | --- |
| Severity and clinical history |  |  |
| HAMD (range: 0-52), M (SD) | 4.93 (4.50) | 4.46 (4.14) |
| Age of depression first onset (years), M (SD) | 24.20 (12.74) | 25.56 (12.03) |
| Severity previous episode^†^ (range: 5-9), M (SD) | 6.76 (1.19) | 6.84 (1.23) |
| Chronicity previous episode (months), M (SD) | 19.97 (26.21) | 18.27 (23.97) |
| Comorbidities, M (SD) | 0.61 (0.86) | 0.60 (0.95) |
| History of abuse, *no*, n (%) | 105 (50.0) | 101 (47.6) |
| Suicide, *no*, n (%) | 161 (76.3) | 159 (75.7) |
| Previous episodes, *≤5*, n (%) | 116 (54.7) | 110 (51.9) |
| Cognitive and emotional |  |  |
| Negative rumination (range: 20-100), M (SD) | 73.97 (15.21) | 72.74 (14.85) |
| Un-resolution rumination (range: 2-20), M (SD) | 12.45 (2.76) | 12.26 (2.79) |
| Self-blame (range: 4-20), M (SD) | 11.43 (3.64) | 10.39 (3.51) |
| (Lack of) acceptance (range: 4-20), M (SD) | 11.82 (3.13) | 11.76 (3.12) |
| Signs recognition (range: 0-4), M (SD) | 1.78 (1.18) | 1.87 (1.25) |
| Awareness (range: 4-40), M (SD) | 20.69 (4.65) | 21.03 (4.94) |
| Self-efficacy (range: 10-50), M (SD) | 32.07 (7.64) | 32.18 (8.10) |
| Positive affect (range: 11-55), M (SD) | 30.90 (7.48) | 31.39 (7.49) |
| Relational and social |  |  |
| Relationship satisfaction (range: 7-35), M (SD) | 27.10 (6.17) | 26.67 (6.80) |
| Stigmatization (range: 7-35), M (SD) | 21.17 (6.57) | 20.55 (6.90) |

^†^ Number of SCID symptoms present when assessing most recent episode. In the test sample, 2 participants did not provide data on history of abuse, 1 participant did not provide data on suicide, 10 participants did not provide data on negative rumination, 10 participants did not provide data on un-resolution rumination, 6 participants did not provide data on self-blame, 6 participants did not provide data on (lack of) acceptance, 8 participants did not provide data on signs recognition, 8 participants did not provide data on awareness, 5 participants did not provide data on self-efficacy, 6 participants did not provide data on positive affect, 12 participants did not provide data on relationship satisfaction, 7 participants did not provide data on stigmatization. In the validation sample, 2 participant did not provide data on suicide, 9 participants did not provide data on negative rumination, 9 participants did not provide data on un-resolution rumination, 10 participants did not provide data on self-blame, 10 participants did not provide data on (lack of) acceptance, 7 participants did not provide data on signs recognition, 7 participants did not provide data on awareness, 9 participants did not provide data on self-efficacy, 10 participants did not provide data on positive affect, 13 participants did not provide data on relationship satisfaction, 12 participants did not provide data on stigmatization.

Table S14*c***:** *Baseline predictors of latent profiles by study group.*

|  | **m-ADM (n = 212)** | **MBCT (n = 212)** |
| --- | --- | --- |
| Severity and clinical history |  |  |
| HAMD (range: 0-52), M (SD) | 4.63 (4.34) | 4.75 (4.33) |
| Age of depression first onset (years), M (SD) | 25.37 (13.26) | 24.39 (11.47) |
| Severity previous episode^†^ (range: 5-9), M (SD) | 6.81 (1.19) | 6.79 (1.23) |
| Chronicity previous episode (months), M (SD) | 17.08 (22.98) | 21.16 (26.96) |
| Comorbidities, M (SD) | 0.67 (0.95) | 0.54 (0.86) |
| History of abuse, *no*, n (%) | 101 (47.6) | 105 (50.0) |
| Suicide, *no*, n (%) | 157 (74.8) | 163 (77.3) |
| Previous episodes, *≤5*, n (%) | 106 (50.0) | 120 (56.6) |
| Cognitive and emotional |  |  |
| Negative rumination (range: 20-100), M (SD) | 73.10 (15.27) | 73.60 (14.82) |
| Un-resolution rumination (range: 2-20), M (SD) | 12.26 (2.68) | 12.44 (2.86) |
| Self-blame (range: 4-20), M (SD) | 10.76 (3.45) | 11.07 (3.76) |
| (Lack of) acceptance (range: 4-20), M (SD) | 11.78 (3.06) | 11.80 (3.18) |
| Signs recognition (range: 0-4), M (SD) | 1.85 (1.23) | 1.80 (1.21) |
| Awareness (range: 4-40), M (SD) | 20.78 (4.64) | 20.94 (4.95) |
| Self-efficacy (range: 10-50), M (SD) | 32.12 (7.73) | 32.13 (8.00) |
| Positive affect (range: 11-55), M (SD) | 31.44 (7.34) | 30.85 (7.62) |
| Relational and social |  |  |
| Relationship satisfaction (range: 7-35), M (SD) | 26.43 (6.81) | 27.34 (6.14) |
| Stigmatization (range: 7-35), M (SD) | 21.01 (6.39) | 20.71 (7.07) |

MBCT=mindfulness-based cognitive therapy (with support to taper or discontinue antidepressant medication). m-ADM=maintenance antidepressant medication. ^†^ Number of SCID symptoms present when considering the most recent depressive episode when assessing most recent episode. In m-ADM, 2 participants did not provide data on suicide, 15 participants did not provide data on negative rumination, 15 participants did not provide data on un-resolution rumination, 11 participants did not provide data on self-blame, 11 participants did not provide data on (lack of) acceptance, 10 participants did not provide data on signs recognition, 10 participants did not provide data on awareness, 10 participants did not provide data on self-efficacy, 11 participants did not provide data on positive affect, 14 participants did not provide data on relationship satisfaction, 11 participants did not provide data on stigmatization. In MBCT, 2 participants did not provide data on history of abuse, 1 participant did not provide data on suicide, 4 participants did not provide data on negative rumination, 4 participants did not provide data on un-resolution rumination, 5 participants did not provide data on self-blame, 5 participants did not provide data on (lack of) acceptance, 5 participants did not provide data on signs recognition, 5 participants did not provide data on awareness, 4 participants did not provide data on self-efficacy, 5 participants did not provide data on positive affect, 11 participants did not provide data on relationship satisfaction, 8 participants did not provide data on stigmatization.

Table S14*d***:** *Fit indices for models containing 1 to 5 latent profiles in the test, validation, and total samples.*

| Test sample (N = 212) | | | | | | | | | |
| --- | --- | --- | --- | --- | --- | --- | --- | --- | --- |
| LPs | AIC | CAIC | BIC | sBIC | LL | Npar | LMR-LRT | BLRT | Entropy |
| 1 | 19482.69 | 19451.03 | 19593.46 | 19488.90 | -9708.35 | 33 | - | - | - |
| 2 | 19308.56 | 19256.79 | 19483.10 | 19318.33 | -9602.28 | 52 | 210.07 (.259) | <0.001 | 0.72 |
| 3 | 19152.72 | 19190.05 | 19391.04 | 19166.07 | -9505.36 | 71 | 191.95 (.372) | <0.001 | 0.83 |
| 4 | 19091.84 | 19002.26 | 19393.94 | 19108.76 | -9455.92 | 90 | 98.15 (.667) | <0.001 | 0.82 |
| 5 | 19065.57 | 18956.98 | 19431.43 | 19086.05 | -9423.78 | 109 | 62.02 (.782) | <0.001 | 0.81 |
| Validation sample (N = 212) | | | | | | | | | |
| LPs | AIC | CAIC | BIC | sBIC | LL | Npar | LMR-LRT | BLRT | Entropy |
| 1 | 19224.68 | 19191.84 | 19335.44 | 19230.88 | -9579.34 | 33 | - | - | - |
| 2 | 18940.81 | 18889.06 | 19115.36 | 18950.58 | -9418.41 | 52 | 318.73 (.052) | <0.001 | 0.78 |
| 3 | 18779.51 | 18708.84 | 19017.82 | 18792.85 | -9318.75 | 71 | 197.36 (.228) | <0.001 | 0.87 |
| 4 | 18681.70 | 18592.12 | 18983.80 | 18698.62 | -9250.85 | 90 | 134.48 (.419) | <0.001 | 0.89 |
| 5 | 18662.28 | 18779.06 | 19028.15 | 18682.77 | -9222.14 | 109 | 56.86 (.639) | <0.001 | 0.88 |
| Total sample (N = 424) | | | | | | | | | |
| LPs | AIC | CAIC | BIC | sBIC | LL | Npar | LMR-LRT | BLRT | Entropy |
| 1 | 38706.16 | 38673.24 | 38839.80 | 38735.08 | -19320.08 | 33 | - | - | - |
| 2 | 38244.70 | 38191.82 | 38455.29 | 38290.27 | -19070.35 | 52 | 495.16 (.222) | <0.001 | 0.72 |
| 3 | 38030.90 | 38060.09 | 38318.43 | 38093.13 | -18944.45 | 71 | 251.80 (.119) | <0.001 | 0.82 |
| 4 | 37833.95 | 37744.15 | 38198.43 | 37912.82 | -18826.97 | 90 | 249.15 (.255) | <0.001 | 0.86 |
| 5 | 37687.57 | 37578.80 | 38128.99 | 37783.09 | -18734.79 | 109 | 183.12 (.666) | <0.001 | 0.86 |

LPs = latent profiles. AIC = Akaike information criterion. CAIC = consistent Akaike information criterion. BIC = Bayesian information criterion. sBIC = sample-size-adjusted BIC. LL = Loglikelihood value. Npar = Number of free parameters. LMR-LRT = Lo–Mendell–Rubin adjusted likelihood ratio test. BLRT = bootstrapped likelihood ratio test. Entropy = index of classification accuracy.

Graph S14*e***:** Elbow plot for LPA involving the test, validation, and total sample.

| Test sample (N = 212)   |
| --- |

| Validation sample (N = 212)   |
| --- |

| Total sample (N = 424)   |
| --- |
| AIC = Akaike information criterion. CAIC = consistent Akaike information criterion. BIC = Bayesian information criterion. sBIC = sample-size-adjusted BIC. |

Table S14*f***:** LPs and predictive factors in the test and validation samples.

| **Test sample** | **LPI (n = 123)** | **LPII (n = 89)** |
| --- | --- | --- |
| Severity and clinical history |  |  |
| HAMD (range: 0-52), M (SE) | 3.71 (0.41) | 6.69 (0.65) |
| Age of depression first onset (years), M (SE) | 26.10 (1.32) | 21.46 (1.41) |
| Severity previous episode^†^ (range: 5-9), M (SE) | 6.46 (0.13) | 7.21 (0.14) |
| Chronicity previous episode (months), M (SE) | 20.73 (2.74) | 18.88 (3.29) |
| Comorbidities, M (SE) | 0.43 (0.07) | 0.86 (0.14) |
| History of abuse, *no*, % (SE) | 63.5 (0.06) | 30.4 (0.07) |
| Suicide, *no*, % (SE) | 82.3 (0.04) | 67.6 (0.06) |
| Previous episodes, *≤ 5*, % (SE) | 64.5 (0.05) | 40.6 (0.06) |
| Cognitive and emotional |  |  |
| Negative rumination (range: 20-100), M (SE) | 69.79 (1.79) | 80.10 (1.84) |
| Un-resolution rumination (range: 4-20), M (SE) | 11.70 (0.30) | 13.55 (0.36) |
| Self-blame (range: 4-20), M (SE) | 9.69 (0.41) | 14.00 (0.66) |
| (Lack of) acceptance (range: 4-20), M (SE) | 11.18 (0.29) | 12.77 (0.54) |
| Signs recognition (range: 0-4), M (SE) | 1.90 (0.13) | 1.61 (0.13) |
| Awareness (range: 4-40), M (SE) | 28.29 (1.06) | 20.18 (0.76) |
| Self-efficacy (range: 10-50), M (SE) | 33.74 (0.83) | 29.66 (0.99) |
| Positive affect (range: 11-55), M (SE) | 32.64 (0.87) | 28.34 (0.84) |
| Relational and social |  |  |
| Relationship satisfaction (range: 7-35), M (SE) | 28.34 (0.66) | 25.22 (0.97) |
| Stigmatization (range: 7-35), M (SE) | 18.06 (0.74) | 25.71 (0.96) |
| **Validation sample** | **LPI (n = 134)** | **LPII (n = 78)** |
| Severity and clinical history |  |  |
| HAMD (range: 0-52), M (SE) | 2.83 (0.41) | 5.39 (0.52) |
| Age of depression first onset (years), M (SE) | 28.16 (1.84) | 24.07 (1.56) |
| Severity previous episode^†^ (range: 5-9), M (SE) | 6.21 (0.15) | 7.20 (0.17) |
| Chronicity previous episode (months), M (SE) | 21.21 (3.98) | 16.59 (1.74) |
| Comorbidities, M (SE) | 0.22 (0.07) | 0.82 (0.13) |
| History of abuse, *no*, % (SE) | 71.1 (0.06) | 34.3 (0.06) |
| Suicide, *no*, % (SE) | 86.9 (0.05) | 69.3 (0.05) |
| Previous episodes, *≤ 5*, % (SE) | 52.8 (0.07) | 51.4 (0.05) |
| Cognitive and emotional |  |  |
| Negative rumination (range: 20-100), M (SE) | 62.35 (3.18) | 78.58 (1.62) |
| Un-resolution rumination (range: 4-20), M (SE) | 10.31 (0.72) | 13.35 (0.23) |
| Self-blame (range: 4-20), M (SE) | 8.20 (0.45) | 11.65 (0.48) |
| (Lack of) acceptance (range: 4-20), M (SE) | 10.78 (0.36) | 12.32 (0.35) |
| Signs recognition (range: 0-4), M (SE) | 2.07 (0.21) | 1.76 (0.12) |
| Awareness (range: 4-40), M (SE) | 30.36 (1.27) | 23.02 (0.75) |
| Self-efficacy (range: 10-50), M (SE) | 34.26 (1.14) | 30.99 (0.82) |
| Positive affect (range: 11-55), M (SE) | 35.48 (1.69) | 29.03 (0.70) |
| Relational and social |  |  |
| Relationship satisfaction (range: 7-35), M (SE) | 30.16 (1.21) | 24.65 (0.68) |
| Stigmatization (range: 7-35), M (SE) | 15.62 (1.33) | 23.31 (0.83) |

^†^ Number of SCID symptoms present when considering the most recent depressive episode.

Table S14*g***:** LPs and predictive factors in the total sample.

| **Total sample** | **LPI (n = 257)** | **LPII (n = 167)** |  | **ES** | **95% CI** |
| --- | --- | --- | --- | --- | --- |
| Severity and clinical history |  |  |  |  |  |
| HAMD (range: 0-52), M (SE) | 3.47 (0.42) | 6.45 (0.68) |  | -0.39 | -0.59, -0.20 |
| Age of depression first onset (years), M (SE) | 27.15 (1.16) | 21.63 (1.31) |  | 0.32 | 0.13, 0.52 |
| Severity previous episode^†^ (range: 5-9), M (SE) | 6.44 (0.21) | 7.33 (0.11) |  | -0.32 | -0.52, -0.13 |
| Chronicity previous episode (months), M (SE) | 20.70 (1.78) | 16.85 (2.45) |  | 0.17 | -0.02, 0.37 |
| Comorbidities, M (SE) | 0.35 (0.05) | 0.98 (0.22) |  | -0.33 | -0.53, -0.14 |
| History of abuse, *no*, % (SE) | 63.4 (0.06) | 27.8 (0.07) |  | 0.84 | 0.64, 1.04 |
| Suicide, *no*, % (SE) | 83.9 (0.04) | 64.6 (0.05) |  | 0.58 | 0.38, 0.78 |
| Previous episodes, *≤ 5*, % (SE) | 61.2 (0.04) | 41.9 (0.07) |  | 0.43 | 0.23, 0.63 |
| Cognitive and emotional |  |  |  |  |  |
| Negative rumination (range: 20-100), M (SE) | 68.19 (1.97) | 80.80 (2.60) |  | -0.39 | -0.59, -0.19 |
| Un-resolution rumination (range: 4-20), M (SE) | 11.52 (0.38) | 13.54 (0.35) |  | -0.37 | -0.56, -0.17 |
| Self-blame (range: 4-20), M (SE) | 9.22 (0.36) | 13.39 (1.12) |  | -0.41 | -0.61, -0.21 |
| (Lack of) acceptance (range: 4-20), M (SE) | 11.10 (0.21) | 12.79 (0.67) |  | -0.28 | -0.48, -0.08 |
| Signs recognition (range: 0-4), M (SE) | 1.91 (0.12) | 1.71 (0.10) |  | 0.12 | -0.08, 0.31 |
| Awareness (range: 4-40), M (SE) | 28.36 (1.14) | 20.98 (1.26) |  | 0.36 | 0.17, 0.56 |
| Self-efficacy (range: 10-50), M (SE) | 33.72 (0.74) | 29.83 (1.08) |  | 0.31 | 0.11, 0.50 |
| Positive affect (range: 11-55), M (SE) | 33.10 (1.07) | 28.29 (0.78) |  | 0.33 | 0.13, 0.52 |
| Relational and social |  |  |  |  |  |
| Relationship satisfaction (range: 7-35), M (SE) | 28.18 (0.81) | 24.95 (0.69) |  | 0.28 | 0.08, 0.48 |
| Stigmatization (range: 7-35), M (SE) | 17.96 (1.20) | 25.04 (1.06) |  | -0.41 | -0.61, -0.21 |

^†^ Number of SCID symptoms present when considering the most recent depressive episode.

ESs (effect sizes) are Cohen’s d (ESs for categorical data were transformed from log(ORs) following Borenstein et al (2009).^52^

Table S14*h*: Latent Profiles and participant sociodemographic characteristics in the test and validation samples.

| **Test sample** | **LPI (n = 123)** | **LPII (n = 89)** |
| --- | --- | --- |
| Demographic characteristics |  |  |
| Age, M (SD) | 49.28 (13.06) | 48.24 (11.49) |
| Gender, *Women*, n (%) | 101 (82.1) | 70 (78.7) |
| Ethnicity, *White*, n (%) | 122 (99.2) | 89 (100) |
| Marital status |  |  |
| *Single, n (%)* | 29 (23.8) | 21 (23.6) |
| *Married, cohabiting, civil partnership,* n (%) | 68 (55.7) | 55 (61.8) |
| *Separated, divorced, widowed,* n (%) | 25 (20.5) | 13 (14.6) |
| Education |  |  |
| *No educational qualification,* n (%) | 4 (3.3) | 5 (5.8) |
| *O levels or GCSEs,* n (%) | 19 (15.6) | 22 (25.6) |
| *AS and A levels or vocational qualification,* n (%) | 54 (44.2) | 32 (37.2) |
| *University training,* n (%) | 45 (36.9) | 27 (31.4) |
| Employment status |  |  |
| *Not working,* n (%) | 46 (38.0) | 33 (37.1) |
| *Part time,* n (%) | 42 (34.7) | 29 (32.6) |
| *Full time,* n (%) | 33 (27.3) | 27 (30.3) |
| Quality of life |  |  |
| Quality of life rating (range: 1-5), M (SD) | 3.82 (0.83) | 3.49 (0.76) |
| Health satisfaction (range: 1-5), M (SD) | 3.26 (0.90) | 2.60 (0.87) |
| Physical (range: 4-20), M (SD) | 15.32 (5.12) | 13.42 (2.84) |
| Psychological (range: 4-20), M (SD) | 13.13 (2.47) | 11.46 (2.25) |
| Social (range: 4-20), M (SD) | 13.88 (3.17) | 11.94 (3.26) |
| Environment (range: 4-20), M (SD) | 15.42 (2.46) | 14.46 (2.62) |
| Mindfulness skills |  |  |
| FFMQ (range: 39-195), M (SD) | 123.61 (17.35) | 109..34 (15.69) |
| Depressive symptoms |  |  |
| BDI-II (range: 0-63), M (SD) | 11.10 (9.10) | 18.01 (9.36) |
| **Validation sample** | **LPI (n = 134)** | **LPII (n = 78)** |
| Demographic characteristics |  |  |
| Age, M (SD) | 50.75 (12.67) | 48.81 (11.33) |
| Gender, *Women*, n (%) | 96 (71.6) | 58 (74.4) |
| Ethnicity, *White*, n (%) | 131 (97.8) | 78 (100) |
| Marital status |  |  |
| *Single, n (%)* | 22 (16.5) | 8 (10.3) |
| *Married, cohabiting, civil partnership,* n (%) | 91 (68.5) | 51 (65.4) |
| *Separated, divorced, widowed,* n (%) | 20 (15.0) | 19 (24.4) |
| Education |  |  |
| *No educational qualification,* n (%) | 7 (5.3) | 4 (5.3) |
| *O levels or GCSEs,* n (%) | 22 (16.8) | 18 (23.7) |
| *AS and A levels or vocational qualification,* n (%) | 58 (44.3) | 32 (42.1) |
| *University training,* n (%) | 44 (33.6) | 22 (28.9) |
| Employment status |  |  |
| *Not working,* n (%) | 60 (45.1) | 41 (52.6) |
| *Part time,* n (%) | 36 (27.1) | 16 (20.5) |
| *Full time,* n (%) | 37 (27.8) | 21 (26.9) |
| Quality of life |  |  |
| Quality of life rating (range: 1-5), M (SD) | 3.84 (0.77) | 3.43 (0.85) |
| Health satisfaction (range: 1-5), M (SD) | 3.17 (1.05) | 2.74 (1.06) |
| Physical (range: 4-20), M (SD) | 15.04 (5.89) | 13.19 (8.48) |
| Psychological (range: 4-20), M (SD) | 13.31 (2.53) | 11.11 (2.42) |
| Social (range: 4-20), M (SD) | 14.25 (3.24) | 12.08 (3.47) |
| Environment (range: 4-20), M (SD) | 15.59 (2.24) | 14.23 (2.39) |
| Mindfulness skills |  |  |
| FFMQ (range: 39-195), M (SD) | 124.22 (17.46) | 111.61 (15.71) |
| Depressive symptoms |  |  |
| BDI-II (range: 0-63), M (SD) | 10.35 (8.67) | 20.92 (10.27) |

GCSE = general certificate of secondary education. FFMQ = Five Facets of Mindfulness Questionnaire. BDI-II = Beck Depression Inventory-II. In the Test sample, 1 participant did not provide data on marital status, 4 participants did not provide data on education, 2 participants did not provide data on employment status, 4 participants did not provide data on any quality-of-life measure, 8 participants did not provide data on FFMQ, and 3 participants did not provide data on BDI-II. In the Validation sample, 1 participant did not provide data on marital status, 5 participants did not provide data on education, 1 participant did not provide data on employment status, 6 participants did not provide data on any quality-of-life measure, 7 participants did not provide data on FFMQ, and 5 participants did not provide data on BDI-II.

Table S14*i*: Latent Profiles and participant sociodemographic characteristics in the total sample.

| **Total sample** | **LPI (n = 257)** | **LPII (n = 167)** |
| --- | --- | --- |
| Demographic characteristics |  |  |
| Age, M (SD) | 50.04 (12.86) | 48.50 (11.38) |
| Gender, *Women*, n (%) | 197 (76.7) | 128 (76.6) |
| Ethnicity, *White*, n (%) | 253 (98.4) | 167 (100) |
| Marital status |  |  |
| *Single, n (%)* | 51 (20.0) | 29 (17.4) |
| *Married, cohabiting, civil partnership,* n (%) | 159 (62.4) | 106 (63.5) |
| *Separated, divorced, widowed,* n (%) | 45 (17.6) | 32 (19.1) |
| Education |  |  |
| *No educational qualification,* n (%) | 11 (4.3) | 9 (5.6) |
| *O levels or GCSEs,* n (%) | 41 (16.2) | 40 (24.7) |
| *AS and A levels or vocational qualification,* n (%) | 112 (44.3) | 64 (39.5) |
| *University training,* n (%) | 89 (35.1) | 49 (30.3) |
| Employment status |  |  |
| *Not working,* n (%) | 106 (41.7) | 74 (44.3) |
| *Part time,* n (%) | 78 (30.7) | 45 (26.9) |
| *Full time,* n (%) | 70 (27.6) | 48 (28.7) |
| Quality of life |  |  |
| Quality of life rating (range: 1-5), M (SD) | 3.83 (0.79) | 3.47 (0.80) |
| Health satisfaction (range: 1-5), M (SD) | 3.21 (0.98) | 2.66 (0.96) |
| Physical (range: 4-20), M (SD) | 15.18 (5.52) | 13.32 (6.13) |
| Psychological (range: 4-20), M (SD) | 13.22 (2.50) | 11.30 (2.33) |
| Social (range: 4-20), M (SD) | 14.07 (3.21) | 12.00 (3.35) |
| Environment (range: 4-20), M (SD) | 15.51 (2.35) | 14.35 (2.51) |
| Mindfulness skills |  |  |
| FFMQ (range: 39-195), M (SD) | 123.93 (17.37) | 110.41 (15.69) |
| Depressive symptoms |  |  |
| BDI-II (range: 0-63), M (SD) | 10.71 (8.77) | 19.37 (9.87) |

GCSE = general certificate of secondary education. FFMQ = Five Facets of Mindfulness Questionnaire. BDI-II = Beck Depression Inventory-II. Two participants did not provide data on marital status, 9 participants did not provide data on education, 3 participants did not provide data on employment status, 10 participants did not provide data on any quality-of-life measure, 15 participants did not provide data on FFMQ, and 8 participants did not provide data on BDI-II. Only differences in quality-of-life, mindfulness skills, and depressive symptoms were statistically significant (all p < 0.001, except for ‘Physical’ with p < 0.01).

Table S14*j*: Average Latent Class Probabilities for Most Likely Latent Class Membership (Row) by Latent Class (Column).

| Test sample (N = 212) | | |
| --- | --- | --- |
| Latent Profiles | **I** | **II** |
| **I** | 0.92 | 0.08 |
| **II** | 0.08 | 0.92 |
| Validation sample (N = 212) | | |
| Latent Profiles | **I** | **II** |
| **I** | 0.92 | 0.08 |
| **II** | 0.05 | 0.95 |
| Total sample (N = 424) | | |
| Latent Profiles | **I** | **II** |
| **I** | 0.92 | 0.08 |
| **II** | 0.09 | 0.91 |

Latent Profile I: lower depression severity.

Latent Profile II: higher depression severity.

**Supplement S15: Latent Profiles and Baseline Characteristics by trial arm.**

|  | **LP I** (lower depression severity) | | **LP II** (higher depression severity) | |
| --- | --- | --- | --- | --- |
| Variables | **m-ADM**  (N = 127) | **MBCT**  (N = 130) | **m-ADM**  (N = 85) | **MBCT**  (N = 82) |
| Demographic characteristics |  |  |  |  |
| Age, M (SD) | 49.01 (13.57) | 51.05 (12.09) | 48.27 (11.43) | 48.74 (11.39) |
| Gender, *Women*, n (%) | 106 (83.5) | 91 (70.0) | 68 (80.0) | 60 (73.2) |
| Ethnicity, *White*, n (%) | 125 (98.4) | 128 (98.5) | 85 (100) | 82 (100) |
| Marital status |  |  |  |  |
| *Single, n (%)* | 24 (19.0) | 27 (20.9) | 14 (16.5) | 15 (18.3) |
| *Married, cohabiting, civil partnership,* n (%) | 82 (65.1) | 77 (59.7) | 58 (68.2) | 48 (58.6) |
| *Separated, divorced, widowed,* n (%) | 20 (15.9) | 25 (19.4) | 13 (15.3) | 19 (23.1) |
| Education |  |  |  |  |
| *No educational qualification,* n (%) | 4 (3.2) | 7 (5.5) | 6 (7.3) | 3 (3.8) |
| *O levels or GCSEs,* n (%) | 25 (19.8) | 16 (12.6) | 20 (24.4) | 20 (25.0) |
| *AS and A levels or vocational qualification,* n (%) | 60 (47.6) | 52 (41.0) | 32 (39.0) | 32 (40.1) |
| *University training,* n (%) | 37 (29.3) | 52 (40.9) | 24 (29.2) | 25 (31.3) |
| Employment status |  |  |  |  |
| *Not working,* n (%) | 45 (35.7) | 61 (47.7) | 37 (43.5) | 37 (45.1) |
| *Part time,* n (%) | 42 (33.3) | 36 (28.1) | 28 (32.9) | 17 (20.7) |
| *Full time,* n (%) | 39 (31.0) | 31 (24.2) | 20 (23.5) | 31 (34.1) |
| Quality of life |  |  |  |  |
| Quality of life rating (range: 1-5), M (SD) | 3.85 (0.86) | 3.81 (0.73) | 3.52 (0.76) | 3.41 (0.85) |
| Health satisfaction (range: 1-5), M (SD) | 3.30 (0.96) | 3.13 (0.99) | 2.73 (0.93) | 2.59 (0.99) |
| Physical (range: 4-20), M (SD) | 15.44 (5.89) | 14.93 (5.15) | 12.88 (2.87) | 13.76 (8.21) |
| Psychological (range: 4-20), M (SD) | 13.08 (2.55) | 13.36 (2.45) | 11.18 (2.20) | 11.42 (2.46) |
| Social (range: 4-20), M (SD) | 13.75 (3.28) | 14.39 (3.12) | 12.19 (3.43) | 11.82 (3.28) |
| Environment (range: 4-20), M (SD) | 15.66 (2.42) | 15.36 (2.28) | 14.21 (2.55) | 14.49 (2.47) |
| Mindfulness skills |  |  |  |  |
| FFMQ (range: 39-195), M (SD) | 122.52 (16.94) | 125.27 (17.73) | 111.09 (15.35) | 109.73 (16.10) |
| Depressive symptoms |  |  |  |  |
| BDI-II (range: 0-63), M (SD) | 10.83 (8.89) | 10.60 (8.68) | 19.91 (9.28) | 18.81 (10.47) |

LP=latent profile. MBCT=mindfulness-based cognitive therapy (with support to taper or discontinue antidepressant medication). m-ADM=maintenance antidepressant medication. GCSE=general certificate of secondary education. BDI-II=Beck Depression Inventory-II.

**Supplement S16: Latent Profiles and Predictive Factors by Trial Arm.**

|  | **LP I** (lower depression severity) | | **LP II** (higher depression severity) | |
| --- | --- | --- | --- | --- |
| Variables | **m-ADM**  (N = 127) | **MBCT**  (N = 130) | **m-ADM**  (N = 85) | **MBCT**  (N = 82) |
| Severity and clinical history |  |  |  |  |
| HAMD (range: 0-52), M (SD) | 3.69 (3.96) | 3.78 (3.43) | 6.05 (4.51) | 6.30 (5.10) |
| Age of depression first onset (years), M (SD) | 27.91 (13.81) | 25.75 (11.95) | 21.58 (11.46) | 22.23 (10.38) |
| Severity previous episode^†^ (range: 5-9), M (SD) | 6.48 (1.18) | 6.45 (1.12) | 7.31 (1.01) | 7.33 (1.22) |
| Chronicity previous episode (months), M (SD) | 19.65 (27.30) | 24.72 (30.30) | 13.25 (13.54) | 13.50 (19.43) |
| Comorbidities, M (SD) | 0.40 (0.70) | 0.37 (0.70) | 1.08 (1.10) | 0.80 (1.09) |
| History of abuse, *no*, n (%) | 79 (62.2) | 78 (60.5) | 22 (25.9) | 27 (33.3) |
| Suicide, *no*, n (%) | 104 (82.5) | 111 (86.0) | 53 (63.1) | 52 (63.4) |
| Previous episodes, *≤ 5*, n (%) | 77 (60.6) | 81 (62.3) | 29 (34.1) | 39 (47.6) |
| Cognitive and emotional |  |  |  |  |
| Negative rumination (range: 20-100), M (SE) | 68.85 (15.30) | 69.27 (14.63) | 79.44 (12.92) | 80.53 (12.36) |
| Un-resolution rumination (range: 4-20), M (SE) | 11.65 (2.72) | 11.66 (2.59) | 13.16 (2.37) | 13.68 (2.85) |
| Self-blame (range: 4-20), M (SE) | 9.47 (2.69) | 9.48 (2.93) | 12.75 (3.57) | 13.60 (3.55) |
| (Lack of) acceptance (range: 4-20), M (SE) | 11.22 (2.75) | 11.36 (3.16) | 12.63 (3.33) | 12.50 (3.12) |
| Signs recognition (range: 0-4), M (SE) | 1.98 (1.31) | 1.86 (1.23) | 1.67 (1.07) | 1.72 (1.16) |
| Awareness (range: 4-40), M (SE) | 22.02 (4.36) | 22.14 (4.92) | 18.93 (4.44) | 19.02 (4.41) |
| Self-efficacy (range: 10-50), M (SE) | 33.27 (7.22) | 34.05 (7.24) | 30.40 (8.19) | 29.11 (8.25) |
| Positive affect (range: 11-55), M (SE) | 32.56 (7.36) | 32.93 (7.51) | 29.72 (7.00) | 27.55 (6.58) |
| Relational and social |  |  |  |  |
| Relationship satisfaction (range: 7-35), M (SE) | 27.52 (6.60) | 28.95 (5.18) | 24.76 (6.84) | 24.68 (6.67) |
| Stigmatization (range: 7-35), M (SE) | 18.93 (6.12) | 17.99 (6.26) | 24.23 (5.43) | 25.01 (6.09) |

^†^ Number of SCID symptoms present when considering the most recent depressive episode. LP=latent profile. MBCT=mindfulness-based cognitive therapy (with support to taper or discontinue antidepressant medication). m-ADM=maintenance antidepressant medication.

**Supplement S17: Details for the latent growth curve model results.**

The raw mean scores of depressive symptoms (BDI-II) over time (*Table S17a*) revealed a decreasing trend in the first three months, dropping from 14.10 to 11.92. Subsequently, a further decrease was observed at nine months (10.77), followed by an increase at 12 months (10.98), gradually increasing at 18 months (11.52), and reaching 11.75 at 24 months. This pattern visually suggested that scores followed a nonlinear growth curve, improving rapidly during the intervention period, levelling off in the initial phase of follow-up, and slightly worsening towards the end of follow-up. A graphical representation of the raw mean scores of symptoms of depression (BDI-II) over time by group and LP can be found in *Figure S17b*.

We evaluated several LGCMs (intercept-only, linear, and quadratic), and tested the structure of the error terms (homoscedastic, heteroscedastic, heteroscedastic & auto-correlated) using fit indices and deviance statistics to determine which model more accurately reflected the change in depressive symptoms in our sample of patients with recurrent depression. As shown in *Table S17c*, the (heteroscedastic & auto-correlated) quadratic model outperformed the intercept-only model and the linear model in all parameters. Therefore, it appeared to be the best fit for the data, and we modelled the change of depressive symptoms over time as a quadratic curve (*Figure S17d*). A heteroscedastic auto-correlated pattern in the error terms reflects systematic variation in the variability of errors over time. Specifically, this means that not only the variances of errors change across different time points, but there is also a structured correlation between consecutive error terms, indicating that the errors at one time point are influenced by the errors at previous time points (*Table S17e*). Overall, the heteroscedastic & autocorrelated quadratic LGCM accounted for between 45.1% and 75.9% of the variability in the observed depressive symptoms variables.

An examination of the LGCM parameter estimates, including factor means, variances, and covariances for the intercept (β_0_), linear coefficient (β_1_), and quadratic coefficient (β_2_), was conducted to develop an understanding of the growth trajectory within our patient sample. As depicted in *Table S17f*, all growth factor means were significantly different from zero (β_0_ = 13.64, p < 0.001; β_1_ = -0.30, p < 0.001; β_2_ = 0.01, p < 0.001). This implies that (a) the initial level fell within the cut-off distinguishing mild from minimal depression, (b) there was a significant linear decrease in depressive symptoms over time, and (c) there was a curvature in the growth trajectory of depressive symptoms over time. The interplay between the two slopes captures the curvature and directionality of the latent growth curve. The negative linear slope suggests a decreasing trend in the initial phase of the curve, while the positive quadratic slope indicates that the rate of decrease diminishes and may eventually turn into an increase in the final phase, forming a U-shaped pattern over time.

Furthermore, the factor variances were statistically significant for the intercept (β_0_ = 47.58, p < 0.001), but not for the linear and quadratic slopes (β_1_ = 0.36, p = 0.178; β_2_ = 0.001, p = 0.235). This indicates that some patients started with varying initial levels of depressive symptoms, but there was no individual variability either in the rate of linear decrease, nor in the extent of curvature in the growth trajectory of depressive symptoms over time. These findings underscore the heterogeneity among patients with recurrent depression in their initial level of depressive symptoms, and highlight the need to consider these individual differences, as we have done through our definition of two distinct LPs that include this potential source of heterogeneity.

In addition, the factor covariances between the intercept and linear term (σxy = 0.61, p = 0.563), intercept and quadratic term (σxy = -0.02, p = 0.510), and linear term and the quadratic term (σxy = -0.01, p = 0.232) were not significant. This suggests that irrespective of a patient's initial status, there was a consistent pattern of reduction of depressive symptoms over time. In other words, patients with recurrent depression, regardless of their starting point, experienced a reduction in depressive symptoms. The lack of significance in the covariances between the linear and quadratic terms indicates that the linear reduction and the curvature in the trajectory were independent of each other. This implies that while patients consistently experienced a decrease in depressive symptoms, the rate of reduction did not vary systematically with the extent of curvature in their growth trajectory.

All these results highlight the robustness of the observed reduction pattern of depressive symptoms over time, which seems not to be contingent on the initial status, despite the considerable variability observed in this regard, and also suggests that the linear and quadratic components contribute independently to the overall decline of depressive symptoms over time. This pattern of LGCM parameter estimates was also observed by treatment and LP sub-groups (see *Table S17g,* and *Table S17h*).

Table S17*a*: Raw descriptive data of depressive symptoms (BDI-II) over time.

|  |  | **0mths** | **3mths** | **9mths** | **12mths** | **18mths** | **24mths** |
| --- | --- | --- | --- | --- | --- | --- | --- |
| Total sample | Mn | 14.10 | 11.92 | 10.77 | 10.98 | 11.52 | 11.75 |
|  | SD | 10.13 | 10.53 | 10.11 | 9.58 | 10.63 | 10.77 |
|  | n | 416 | 348 | 293 | 324 | 291 | 336 |
|  |  |  |  |  |  |  |  |
| *m-ADM* | Mn | 14.45 | 13.94 | 10.54 | 11.25 | 11.32 | 11.90 |
|  | SD | 10.07 | 10.93 | 9.70 | 9.15 | 10.73 | 10.66 |
|  | n | 206 | 174 | 142 | 157 | 149 | 167 |
|  |  |  |  |  |  |  |  |
| *MBCT* | Mn | 13.77 | 9.90 | 11.00 | 10.72 | 11.73 | 11.60 |
|  | SD | 10.21 | 9.74 | 10.51 | 9.98 | 10.55 | 10.91 |
|  | n | 210 | 174 | 151 | 167 | 142 | 169 |

MBCT=mindfulness-based cognitive therapy (with support to taper or discontinue antidepressant medication). m-ADM=maintenance antidepressant medication.

Figure S17*b*: Sample means of symptoms of depression over time by group and LP.

Table S17*c*: Tests of Alternative Latent Growth Curve Model Specifications.

| **Model** | **Change function** | **χ^2^** | **df** | **CFI** | **TLI** | **RMSEA**  **(90% CI)** | **SRMR** | **log** | **AIC** | **CAIC** | **BIC** | **sBIC** |
| --- | --- | --- | --- | --- | --- | --- | --- | --- | --- | --- | --- | --- |
| **M1** | *Intercept* |  |  |  |  |  |  |  |  |  |  |  |
| **.M1.1** | *M1 + homoscedastic* | 195.71 | 24 | 0.82 | 0.89 | 0.13  (0.11, 0.15) | 0.10 | -7133.87 | 14273.73 | 14271.75 | 14285.86 | 14276.34 |
| **.M1.2** | *M1 + heteroscedastic* | 122.21 | 19 | 0.89 | 0.92 | 0.11  (0.10, 0.13) | 0.12 | -7097.12 | 14210.23 | 14210.59 | 14242.57 | 14217.19 |
| **.M1.3** | *M1.2 + auto-correlated* | 79.88 | 18 | 0.94 | 0.95 | 0.09  (0.07, 0.11) | 0.11 | -7075.95 | 14169.91 | 14160.43 | 14206.29 | 14177.73 |
| **M2** | *Linear* |  |  |  |  |  |  |  |  |  |  |  |
| **.M2.1** | *M2 + homoscedastic* | 129.08 | 21 | 0.89 | 0.92 | 0.11  (0.09, 0.13) | 0.09 | -7100.55 | 14213.10 | 14207.63 | 14237.36 | 14218.32 |
| **.M2.2** | *M2 + heteroscedastic* | 84.18 | 16 | 0.93 | 0.93 | 0.10  (0.08, 0.12) | 0.09 | -7078.10 | 14178.21 | 14178.85 | 14222.68 | 14187.77 |
| **.M2.3** | *M2.2 + auto-correlated* | 56.10 | 15 | 0.96 | 0.96 | 0.08  (0.06, 0.10) | 0.08 | -7064.06 | 14152.13 | 14140.25 | 14200.64 | 14162.56 |
| **M3** | *Quadratic* |  |  |  |  |  |  |  |  |  |  |  |
| **.M3.1** | *M3 + homoscedastic* | 80.30 | 17 | 0.93 | 0.94 | 0.09  (0.07, 0.12) | 0.07 | -7076.16 | 14172.32 | 14162.34 | 14212.75 | 14181.02 |
| **.M3.2** | *M3 + heteroscedastic* | 34.50 | 12 | 0.98 | 0.97 | 0.06  (0.04, 0.08) | 0.05 | -7053.26 | 14136.53 | 14137.71 | 14197.17 | 14149.57 |
| **.M3.3** | *M3.2 + auto-correlated* | 30.11 | 11 | 0.98 | 0.97 | 0.06  (0.04, 0.08) | 0.05 | -7051.07 | 14134.13 | 14118.18 | 14196.82 | 14148.04 |

M1 assumes there is no systematic change in depressive symptoms over time, implying a constant initial level (intercept) for all participants. M2 includes the intercept factor and a linear trajectory over time. M3 tests for nonlinearity by introducing a quadratic term, in addition to the intercept and linear terms. The homoscedastic sub-model assumes the error terms are equal. The heteroscedastic sub-model allows for the possibility that the variances of errors can vary over time. The auto-correlated sub-model builds upon the heteroscedastic sub-model by introducing an autocorrelation structure between consecutive error terms over time.

Figure S17*d*: Observed and estimated mean trajectories.

Table S17*e*: Pattern of autoregressive residual correlations.

|  | *e1* | *e2* | *e3* | *e4* | *e5* | *e6* |
| --- | --- | --- | --- | --- | --- | --- |
|  |  |  |  |  |  |  |
| *e1* |  |  |  |  |  |  |
| *e2* | 0.09* |  |  |  |  |  |
| *e3* |  | 0.12* |  |  |  |  |
| *e4* |  |  | 0.17* |  |  |  |
| *e5* |  |  |  | 0.16* |  |  |
| *e6* |  |  |  |  | 0.15* |  |
|  |  |  |  |  |  |  |

Values reflect standardised relationships between the error terms after controlling for residual variances. *p <0.05.

Table S17*f*: Growth parameters estimates for the total sample.

|  | μ |  | σ² |
| --- | --- | --- | --- |
|  |  |  |  |
| *Intercept (β_0_)* | 13.64*** |  | 47.58*** |
|  |  |  |  |
| *Linear term (β_1_)* | -0.30*** |  | 0.36 |
|  |  |  |  |
| *Quadratic term (β_2_)* | 0.01*** |  | 0.001 |
|  |  |  |  |
|  | *(β_0_) - (β_1_)* | *(β_0_) - (β_2_)* | *(β_1_) - (β_2_)* |
| σ_xy_ | 0.61 | -0.02 | -0.01 |

Standardizing the manifest variables destroys the ability to examine change because the standardization equates the means and variances.^32-34^ Therefore, we present here the unstandardized estimates (as is most typical in this type of research). ***p < 0.001.

Table S17*g*: Growth parameters estimates by treatment group.

|  | m-ADM (n = 212) | | |  | MBCT (n = 212) | | |
| --- | --- | --- | --- | --- | --- | --- | --- |
|  | μ |  | σ² |  | μ |  | σ² |
|  |  |  |  |  |  |  |  |
| *β_0_* | 14.69*** |  | 51.97*** |  | 12.42*** |  | 41.06** |
|  |  |  |  |  |  |  |  |
| *β_1_* | -0.36*** |  | 0.14 |  | -0.22* |  | 0.56 |
|  |  |  |  |  |  |  |  |
| *β_2_* | 0.01** |  | <0.001 |  | 0.01* |  | 0.001 |
|  |  |  |  |  |  |  |  |
|  | *(β_0_) - (β_1_)* | *(β_0_) - (β_2_)* | *(β_1_) - (β_2_)* |  | *(β_0_) - (β_1_)* | *(β_0_) - (β_2_)* | *(β_1_) - (β_2_)* |
| σ_xy_ | 0.77 | -0.05 | -0.002 |  | 0.63 | -0.01 | -0.02 |

Standardizing the manifest variables destroys the ability to examine change because the standardization equates the means and variances.^32-34^ Therefore, we present here the unstandardized estimates (as is most typical in this type of research). ***p < 0.001. β_0_ = intercept. β_1_ = linear term. β_2_ = quadratic term.

Table S17*h*: Growth parameters estimates by latent profile.

|  | LP1 (n = 257) | | |  | LP2 (n = 167) | | |
| --- | --- | --- | --- | --- | --- | --- | --- |
|  | μ |  | σ² |  | μ |  | σ² |
|  |  |  |  |  |  |  |  |
| *β_0_* | 10.00*** |  | 30.95*** |  | 19.44*** |  | 66.03*** |
|  |  |  |  |  |  |  |  |
| *β_1_* | -0.21* |  | 0.32 |  | -0.52*** |  | 1.31 |
|  |  |  |  |  |  |  |  |
| *β_2_* | 0.01** |  | <0.001 |  | 0.02** |  | 0.001 |
|  |  |  |  |  |  |  |  |
|  | *(β_0_) - (β_1_)* | *(β_0_) - (β_2_)* | *(β_1_) - (β_2_)* |  | *(β_0_) - (β_1_)* | *(β_0_) - (β_2_)* | *(β_1_) - (β_2_)* |
| σ_xy_ | 0.77 | 0.03 | -0.01 |  | -0.84 | -0.001 | -0.04 |

Standardizing the manifest variables destroys the ability to examine change because the standardization equates the means and variances.^32-34^ Therefore, we present here the unstandardized estimates (as is most typical in this type of research). ***p < 0.001. β_0_ = intercept. β_1_ = linear term. β_2_ = quadratic term.

**Supplement S18: Descriptive statistics for pre-post change in mindfulness skills (FFMQ) by group and LP.**

|  |  | **Total** | **m-ADM** | | **MBCT-TS** | |
| --- | --- | --- | --- | --- | --- | --- |
|  |  |  | **LP1** | **LP2** | **LP1** | **LP2** |
|  |  |  |  |  |  |  |
| **T0** | M | 118.61 | 122.52 | 111.09 | 125.27 | 109.73 |
|  | SD | 17.97 | 16.94 | 15.35 | 17.73 | 16.10 |
|  | n | 409 | 121 | 81 | 127 | 80 |
|  |  |  |  |  |  |  |
| **T1** | M | 125.75 | 124.39 | 115.69 | 132.51 | 126.47 |
|  | SD | 19.16 | 17.72 | 16.32 | 17.20 | 22.77 |
|  | n | 348 | 109 | 65 | 112 | 62 |
|  |  |  |  |  |  |  |
| **ΔT0-T1** | M | 6.95 | 2.33 | 4.02 | 6.62 | 18.45 |
|  | SD | 17.54 | 14.50 | 15.70 | 18.57 | 17.45 |
|  | n | 344 | 105 | 65 | 112 | 62 |
|  |  |  |  |  |  |  |

T0 = pre-treatment. T1 = post-treatment (3-month follow-up). ΔT0-T1 = pre-post change scores.

FFMQ = Five Facets of Mindfulness Questionnaire (range: 39-195). MBCT-TS=mindfulness-based cognitive therapy (with support to taper or discontinue antidepressant medication). m-ADM=maintenance antidepressant medication. LP1 = latent profile 1. LP2 = latent profile 2. M = mean. SD = standard deviation. n = numbers.

**Supplement S19: Baseline to post-treatment change of mindfulness skills (FFMQ) as a function of treatment received and LP.**


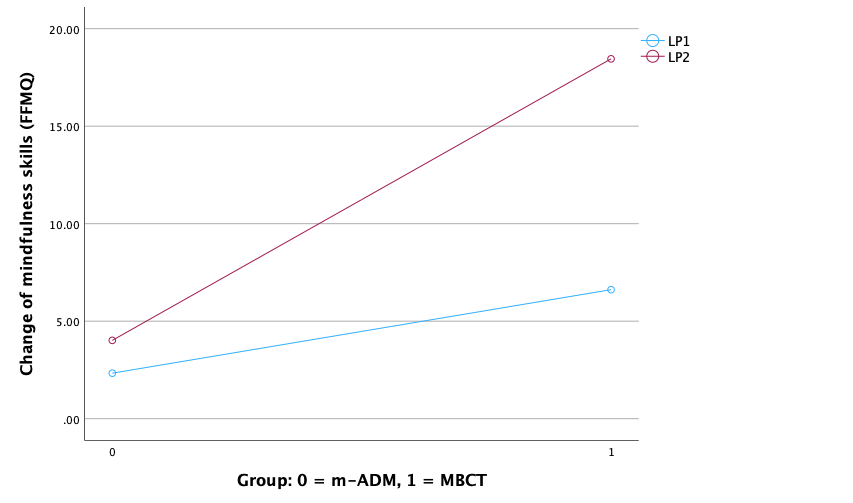


**Supplement S20: Linear slope of depressive symptoms over time as a function of change of mindfulness skills (FFMQ) and LP.**


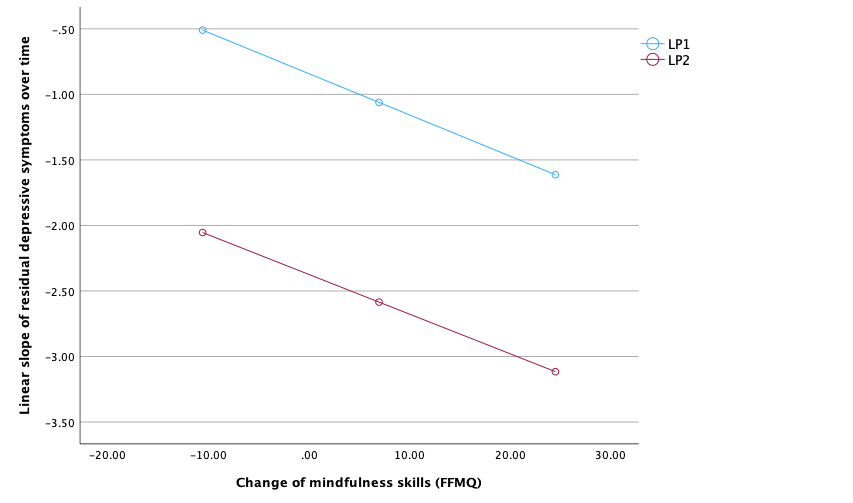


Conditioning values of change of mindfulness skills (FFMQ): Mean ±1SD.

**Supplement S21: ADM use and formal meditation practice by trial arm and LP.**

|  | **m-ADM** | | **MBCT** | |
| --- | --- | --- | --- | --- |
|  | **LP1** | **LP2** | **LP1** | **LP2** |
| **m-ADM adherence** |  |  |  |  |
| *Remained on therapeutic dose* | 93 (73.2) | 69 (81.2) | 42 (32.3) | 37 (45.1) |
| *Did not remain on therapeutic dose* | 34 (26.8) | 16 (18.8) | 88 (67.7) | 45 (54.9) |
|  |  |  |  |  |
| **Formal Meditation** |  |  |  |  |
| *Not at all* | 86 (82.7) | 55 (91.7) | 20 (18.3) | 7 (12.1) |
| *Sometimes* | 12 (11.5) | 4 (6.7) | 35 (32.1) | 19 (32.8) |
| *Regularly* | 3 (2.9) | 0 (0.0) | 24 (22.0) | 13 (22.4) |
| *More days than not* | 3 (2.9) | 1 (1.7) | 30 (27.5) | 19 (32.8) |
|  |  |  |  |  |

Numbers are frequencies and percentages. m-ADM adherence (ADM discontinuation in the models and analyses) refers to whether participants remained on a therapeutic stable dose of antidepressant medication in line with British National Formulary (BNF) and NICE guidance for the duration of the entire study. Formal meditation refers to the following question: “How much have you practised any formal meditation in last month?”. This question was introduced at the post-treatment timepoint (one month after the end of the MBCT training or the equivalent time in the m-ADM arm), and it was not completed by 23 participants in the m-ADM group and LP1, 25 participants in the m-ADM group and LP2, 21 participants in the MBCT group and LP1, and 24 participants in the MBCT group and LP2. In the m-ADM arm, 126 LP1 participants did not attend 4 or more sessions of MBCT, 1 LP1 participant attended 4 or more sessions of MBCT, 85 LP2 participants did not attend 4 or more sessions of MBCT, and 0 LP2 participants attended 4 or more sessions of MBCT. In the MBCT arm, 110 LP1 participants attended 4 or more sessions of MBCT, 20 LP1 participant did not attend 4 or more sessions of MBCT, 66 LP2 participants attended 4 or more sessions of MBCT, and 16 LP2 participants did not attend 4 or more sessions of MBCT.

**Supplement S22: Moderated-mediation sensitivity analysis.**

In the sensitivity moderated-mediation model, we adjusted for the covariates of home-based formal meditation practice and discontinuation in antidepressant intake. The inclusion of these covariates reduced the values of the likelihood-based indices (main model: loglikelihood = -8691.30, AIC = 17432.60, CAIC = 17435.89, BIC = 17533.66, sBIC = 17454.33; sensitivity model: loglikelihood = -7627.17, AIC = 15312.33, CAIC = 15260.12, BIC = 15422.60, sBIC = 15330.61). This means that the sensitivity model improved the fit compared to the main model. A graphical representation of the sensitivity analysis for the moderated-mediation, including only the significant direct and indirect effects can be seen in *Figure S22a*.

Figure S22*a*: Sensitivity analysis for the moderated-mediation.

MBCT = mindfulness-based cognitive therapy (with support to taper or discontinue antidepressant medication). m-ADM = maintenance antidepressant medication. LP1 = latent profile 1 (less severe depression). LP2 = latent profile 2 (more severe depression). ΔFFMQ = baseline to one month after the end of the MBCT training (T0-T1) change in mindfulness skills. β_1_ (BDI-II) = linear slope of depressive symptoms (i.e., rate of change in depressive symptoms) over time, from baseline to two-years follow-up (T0-T5). Meditation Practice = home-based formal meditation practice during last month (‘not at all’, ‘sometimes’, ‘regularly’, ‘more days than not’) measured at post-intervention (i.e., one month after the end of the MBCT training or the equivalent time in the m-ADM arm (T1)). Discontinuation = discontinuation in antidepressant intake over the course of the study (‘yes’, ‘no’). *a*_2_ = allocation group -> ΔFFMQ path for LP2. *a*_3_ = meditation practice -> ΔFFMQ path for LP1. b = ΔFFMQ -> β_1_ (BDI-II) path (this is shared across LPs, as there were not moderating effects in this path). IE = indirect effect. 95% CI = bootstraped 95% Confidence Interval for the indirect effect. c’ = direct effect of trial arm on β_1_ (BDI-II) after adjustment for the covariates and mediating effects (this is shared across LPs, as there were not moderating effects in this path). Coefficients are not standardized, and therefore, maintain the original units of the variables involved in the moderated-mediation model. Dashed lines represent non-significant direct effects (from the covariates to the rate of change in depressive symptoms over time).* p < 0.05; ** p < 0.01; *** p < 0.001.

As illustrated in *Figure S5b*, formal meditation practice was treated as a potential covariate for both the pre-post change in mindfulness skills and the linear slope of depressive symptoms over time. Discontinuation in antidepressant intake was regarded as a potential covariate for the linear slope of depressive symptoms over time. The sample size for this moderated-mediation sensitivity analysis decreased from n = 424 to n = 331, due to missing data in the home-based formal meditation practice covariate (see Supplement S4).

As shown in *Table S22b*, a significant moderating effect was observed between the trial arm and LP in the *a*-path from the trial arm to pre-post change in mindfulness skills (coefficient = 14.00; 95% CI = 3.00, 25.96, p = 0.016). The conditional effect from the trial arm to the pre-post change in mindfulness skills was significant for LP2 (*a_2_* = 13.61; 95% CI = 6.14, 23.03, p = 0.002), but it was non-significant for LP1 (*a_1_* = -0.39; 95% CI = -6.82, 6.22, p = 0.907). There was no significant moderating effects between pre-post improvements in mindfulness skills and LP for the b-path from pre-post change in mindfulness skills to the rate of change in depressive symptoms over time, with a difference between LPs (i.e., moderating coefficient) close to zero (coefficient = 0.002; 95% CI = -0.04, 0.05, p = 0.927). The main effect from pre-post improvements in mindfulness skills to the rate of change in depressive symptoms over time was b = -0.03 (CI = -0.04, -0.01; p < 0.001), which reflected the same value as in the main model. There was no significant moderating effect between trial arm and LP for the direct effect from trial arm to the rate of change in depressive symptoms over time (coefficient = -0.16; 95% CI = -1.34, 1.00; p = 0.796). The direct effect from trial arm to the rate of change in depressive symptoms over time (after controlling for all the indirect effects and covariates) was significant (c’ = 0.71; 95% CI = 0.19, 1.27; p = 0.009), and of the same direction than the total effects from trial arm to the rate of change in depressive symptoms over time (c = 0.36; 95% CI = -0.24, 0.96; p = 0.243). The index of moderated-mediation was significant (coefficient = -0.37; 95% CI = -0.89, -0.08), providing evidence for the LPs to moderate the mediated effect of change in mindfulness skills between trial arm and rate of change in depressive symptoms over time. The conditional indirect effect for LP2 was significant (indirect effect = -0.36; 95% CI = -0.79, -0.13), whilst it was not significant for LP1 (indirect effect = 0.01; 95% CI = -0.16, 0.21). Together, this suggests a greater benefit of MBCT thought the acquisition of mindfulness skills for patients with a higher depression severity.

There was no significant moderating effect between home-based formal meditation practice and LP in the path from formal meditation practice to pre-post change in mindfulness skills (coefficient = -2.44; 95% CI = -7.92, 3.02; p = 0.380). Nevertheless, we continued exploring the potential conditional effects from formal meditation practice to pre-post change in mindfulness skills by LP because the difference observed (i.e., coefficient) suggests this difference might be meaningful (2.44 in absolute value, indicating a difference of 2.44 points in the FFMQ total score between LPs for each unit increase in formal meditation practice, measured on a scale from 0 to 3). We observed that the conditional effect from formal meditation practice to pre-post change in mindfulness skills was not significant for LP2 (*a*_4_ = 1.10; 95% CI = -3.11, 5.13, p = 0.600), but it was significant for LP1 (*a*_3_ = 3.54; 95% CI = 0.53, 6.68, p = 0.024). The index of moderated-mediation from formal meditation practice to the rate of change in depressive symptoms over time, through pre-post improvements in mindfulness skills moderated by LP, was not significant (coefficient = 0.07; 95% CI = -0.07, 0.25). Nevertheless, there were significant conditional indirect effects for LP1 from formal meditation practice to the rate of change in depressive symptoms over time through pre-post change in mindfulness skills (indirect effect = -0.10; 95% CI = -0.22, -0.02); but the conditional indirect effect for LP2 from formal meditation practice to the rate of change in depressive symptoms over time, through pre-post change in mindfulness skills was not significant (indirect effect = -0.03; 95% CI = -0.16, 0.08). These results suggest a potential greater importance of formal meditation practice for patients with a lower depression severity, but the interaction test was not definitive in this regard.

There was no significant moderating effect between formal meditation practice and LP for the direct path from formal meditation practice to the rate of change in depressive symptoms over time (coefficient = 0.05; 95% CI = -0.59, 0.64; p = 0.862). The direct effect from formal meditation practice to the rate of change in depressive symptoms over time was not significant (c = -0.08; 95% CI = -0.33, 0.18; p = 0.528). Similarly, there was no significant moderating effects between discontinuation in antidepressant intake and LP for the direct path from discontinuation in antidepressant intake to the rate of change in depressive symptoms over time (coefficient = -0.40; 95% CI = -1.72, 1.04; p = 0.569). The direct effect from discontinuation in antidepressant intake to the rate of change in depressive symptoms over time was c = -0.47 (95% CI = -0.94, 0.03; p = 0.053).

In summary, results from the moderated-mediation sensitivity analysis consistently demonstrate a significant moderating effect between the trial arm and LP in the *a*-path, indicating the LP's influence on the relationship between the trial arm and pre-post change (i.e., learning) in mindfulness skills. LP1 and LP2 exhibited distinct conditional indirect effects. Patients with a higher depression severity (LP2) reported greater benefits following the MBCT program through the acquisition of mindfulness skills, compared to those with a lower depression severity (LP1).

Table S22*b*: Sensitivity analysis for the moderated-mediation of mindfulness skills.

*Results for the main model variables (after controlling for the covariates)*

| **Direct effects** | **coefficient** | **SE** | **Boot 95% CI** | **p** |
| --- | --- | --- | --- | --- |
| Trial arm * LP -> ΔFFMQ | 14.00 | 5.83 | 3.00, 25.96 | 0.016 |
| Trial arm -> ΔFFMQ (LP1) | -0.39 | 3.31 | -6.82, 6.22 | 0.907 |
| Trial arm -> ΔFFMQ (LP2) | 13.61 | 4.31 | 6.14, 23.03 | 0.002 |
| ΔFFMQ * LP -> β_1_ (BDI-II) | 0.002 | 0.02 | -0.04, 0.05 | 0.927 |
| ΔFFMQ -> β_1_ (BDI-II) | -0.03 | 0.01 | -0.04, -0.01 | <0.001 |
| Trial arm * LP -> β_1_ (BDI-II) | -0.16 | 0.60 | -1.34, 1.00 | 0.796 |
| Trial arm -> β_1_ (BDI-II) | 0.71 | 0.27 | 0.19, 1.27 | 0.009 |
| Total effects (trial arm) | 0.36 | 0.31 | -0.24, 0.96 | 0.243 |
| **Indirect effects** | **coefficient** | **SE** | **Boot LLCI** | **Boot ULCI** |
| LP1 | 0.01 | 0.09 | -0.16 | 0.21 |
| LP2 | -0.36 | 0.16 | -0.79 | -0.13 |
| Difference (index of moderated-mediation) | -0.37 | 0.20 | -0.89 | -0.08 |

*Results for the covariates*

| **Direct effects** | **coefficient** | **SE** | **Boot 95% CI** | **p** |
| --- | --- | --- | --- | --- |
| Meditation Practice * LP -> ΔFFMQ**^†^** | -2.44 | 2.78 | -7.92, 3.02 | 0.380 |
| Meditation Practice -> ΔFFMQ (LP1) | 3.54 | 1.57 | 0.53, 6.68 | 0.024 |
| Meditation Practice -> ΔFFMQ (LP2) | 1.10 | 2.10 | -3.11, 5.13 | 0.600 |
| Meditation Practice * LP -> β_1_ (BDI-II) | 0.05 | 0.31 | -0.59, 0.64 | 0.862 |
| Meditation Practice -> β_1_ (BDI-II) | -0.08 | 0.13 | -0.33, 0.18 | 0.528 |
| Discontinuation * LP -> β_1_ (BDI-II) | -0.40 | 0.71 | -1.72, 1.04 | 0.569 |
| Discontinuation -> β_1_ (BDI-II) | -0.47 | 0.25 | -0.94, 0.03 | 0.053 |
| **Indirect effects^††^** | **coefficient** | **SE** | **Boot LLCI** | **Boot ULCI** |
| LP1 | -0.10 | 0.05 | -0.22 | -0.02 |
| LP2 | -0.03 | 0.06 | -0.16 | 0.08 |
| Difference (index of moderated-mediation) | 0.07 | 0.08 | -0.07 | 0.25 |

SE = standard error. Boot 95% CI = Bootstrap 95% Confidence Interval. Boot LLCI = Bootstrap Lower Limit of (95%) Confidence Interval. Boot ULCI = Bootstrap Upper Limit of (95%) Confidence Interval. LP = latent profile. ΔFFMQ = baseline to post-treatment (i.e., one month after the end of the MBCT training) (T0-T1) change in mindfulness skills. β_1_ (BDI-II) = linear slope of depressive symptoms (i.e., rate of change in depressive symptoms) over time from baseline to two-years follow-up (T0-T5). Difference (index of moderated-mediation) = indirect effects difference by LP. Meditation Practice = formal home-based meditation practice during last month measured at post-intervention (one month after the end of the MBCT training or the equivalent time in the m-ADM arm (T1)). Discontinuation = discontinuation in antidepressant intake over the course of the study. Coefficients are not standardized, and therefore maintain the original units of the variables involved in the moderated-mediation model. **^†^**Although the result of this interaction was not significant, the difference in the effects that were observed in the pre-post change of mindfulness skills (2.44 in absolute value, indicating a difference of 2.44 points in the FFMQ between latent profiles for each unit increase in the formal meditation practice variable, measured on a scale from 0 to 4), suggests a further exploration of the conditional effects between home-based formal meditation practice and pre-post mindfulness improvement based on LP. Therefore, we present here the relationship from formal meditation practice and the pre-post change in mindfulness skills by LP. **^††^** Conditional indirect effects from home-based formal meditation practice to the slope of depressive symptoms (i.e., rate of change in depressive symptoms) over time, mediated by pre-post improvements in mindfulness skills and contingent on LP. N = 331. LP1: R^2^ (M) = 0.06; R^2^ (Y) = 0.11. LP2: R^2^ (M) = 0.19; R^2^ (Y) = 0.09.

**References.**

^1^ Cohen ZD, DeRubeis RJ, Hayes R, Watkins ER, Lewis G, Byng R, Byford S, Crane C, Kuyken W, Dalgleish T, Schweizer S. The development and internal evaluation of a predictive model to identify for whom Mindfulness-Based Cognitive Therapy (MBCT) offers superior relapse prevention for recurrent depression versus maintenance antidepressant medication. Clin Psychol Sci. 2023;11(1):59-76. doi:10.1177/21677026221076832

^2^ Williams JM, Crane C, Barnhofer T, Brennan K, Duggan DS, Fennell MJ, Hackmann A, Krusche A, Muse K, Von Rohr IR, Shah D, Crane RS, Eames C, Jones M, Radford S, Silverton S, Sun Y, Weatherley-Jones E, Whitaker CJ, Russell D, Russell IT. Mindfulness-based cognitive therapy for preventing relapse in recurrent depression: a randomized dismantling trial. J Consult Clin Psychol. 2014;82(2):275-86. doi:10.1037/a0035036

^3^ Hoertel N, Blanco C, Oquendo MA, Wall MM, Olfson M, Falissard B, et al. A comprehensive model of predictors of persistence and recurrence in adults with major depression: Results from a national 3-year prospective study. J Psychiatr Res. 2017;95:19-27. doi: 10.1016/j.jpsychires.2017.07.022

^4^ Steinert C, Hofmann M, Kruse J, Leichsenring F. The prospective long-term course of adult depression in general practice and the community. A systematic literature review. J Affect Disord. 2014;152–154:65-75. doi: 10.1016/j.jad.2013.10.017

^5^ Spijker J, de Graaf R, ten Have M, Nolen WA, Speckens A. Predictors of suicidality in depressive spectrum disorders in the general population: results of the Netherlands Mental Health Survey and Incidence Study. Soc Psychiatry Psychiatr Epidemiol. 2010;45(5):513-21. doi: 10.1007/s00127-009-0093-6

^6^ Patten SB, Wang JL, Williams JV, Lavorato DH, Khaled SM, Bulloch AG. Predictors of the Longitudinal Course of Major Depression in a Canadian Population Sample. Can J Psychiatry. 2010;55(10):669-76. doi: 10.1177/070674371005501006

^7^ Lam RW, Kennedy SH, Grigoriadis S, McIntyre RS, Milev R, Ramasubbu R, Parikh SV, Patten SB, Ravindran AV. Canadian Network for Mood and Anxiety Treatments (CANMAT) clinical guidelines for the management of major depressive disorder in adults. III. Pharmacotherapy. J Affect Disord. 2009;117(Suppl 1):S26-43. doi: 10.1016/j.jad.2009.06.041

^8^ Williams JB. A structured interview guide for the Hamilton Depression Rating Scale. Arch Gen Psychiatry. 198845(8):742-7. doi:10.1001/archpsyc.1988.01800320058007

^9^ Zimmerman M, Posternak MA, Chelminski I. Heterogeneity among depressed outpatients considered to be in remission. Compr Psychiatry. 2007;48(2):113-7. doi:10.1016/j.comppsych.2006.10.005

^10^ Parker G, Roussos J, Hadzi-Pavlovic D, Mitchell P, Wilhelm K, Austin MP. The development of a refined measure of dysfunctional parenting and assessment of its relevance in patients with affective disorders. Psychol Med. 1997;27(5):1193-203. doi:10.1017/s003329179700545x

^11^ Spitzer RL, Williams JBW, Gibbon M, First MB: Structured Clinical Interview for DSM-IV (SCID) Washington DC: American Psychiatric Association; 1996.

^12^ Barnard PJ, Watkins ER, Mackintosh B, Nimmo-Smith I. Getting stuck in a mental rut: Some process and experiential attributes. 35th Congress of the British Association for Behavioural and Cognitive Psychotherapies; 2007

^13^ Garnefski N, Kraaij V, Spinhoven P. Negative life events, cognitive emotion regulation and emotional problems. *Personality and Individual Differences*. 2001;30(8):1311-1327. doi:10.1016/S0191-8869(00)00113-6

^14^ Baer RA, Smith GT, Hopkins J, Krietemeyer J, Toney L. Using self-report assessment methods to explore facets of mindfulness. Assessment. 2006;13(1):27-45 doi:10.1177/1073191105283504

^15^ Schwarzer R, Jerusalem M, Wright S, Johnston M. Generalized self-efficacy scale. In: Measures in health psychology: A user’s portfolio. NFER-Nelson; 1995

^16^ Dunn B, Warbrick L, Hayes R, Montero-Marin J, Reed Nigel, Dalgleish T, Kuyken W. Does Mindfulness-Based Cognitive Therapy with tapering support reduce the risk of relapse/recurrence in major depressive disorder by enhancing positive affect? A secondary analysis of the PREVENT trial. Journal of Consulting and Clinical Psychology (in press).

^17^ Shiota MN, Keltner D, John OP. Positive emotion dispositions differentially associated with Big Five personality and attachment style. The Journal of Positive Psychology. 2006;1(2):61-71. doi:10.1080/17439760500510833

^18^ Development of the World Health Organization WHOQOL-BREF quality of life assessment. The WHOQOL Group. Psychol Med. 1998;28(3):551-558. doi:10.1017/s0033291798006667

^19^ Beck AT, Steer RA, Brown GK. The Beck Depression Inventory, 2nd edn. San Antonio, TX: The Psychological Corporation; 1996

^20^Beck AT, Steer RA, Garbin MG. Psychometric properties of the Beck Depression Inventory: Twenty-five years of evaluation. Clinical Psychology Review. 1988;8(1):77-100

^21^ Morin AJ, Meyer JP, Creusier J, Bietry F. Multiple-group analysis of similarity in latent profile solutions. *Organizational Research Methods*. 2016;19(2):231-254. doi:10.1177/10944281156211

^22^ Nylund KL, Asparouhov T, Muthén BO. Deciding on the number of classes in latent class analysis and growth mixture modeling: A Monte Carlo simulation study. *Structural Equation Modeling: A Multidisciplinary Journal*. 2007;14:535-569. doi:10.1080/10705510701575396

^23^ Nylund-Gibson K, Choi AY. Ten frequently asked questions about latent class analysis. *Translational Issues in Psychological Science*. 2018;4(4):440-461. doi:10.1037/tps0000176

^24^ Bakk Z, Vermunt JK. Robustness of stepwise latent class modeling with continuous distal outcomes. *Structural Equation Modeling: A Multidisciplinary Journal*. 2016;23:20-31. doi: 10.1080/10705511.2014.955104

^25^ Lanza ST, Rhoades BL. Latent class analysis: an alternative perspective on subgroup analysis in prevention and treatment. *Prevention science*. 2013;14(2):157-168. doi:10.1007/s11121-011-0201-1.

^26^ Gudicha D. Power analysis methods for tests in latent class and latent Markov models. Ridderkerk, the Netherlands: Ridderprint BV; 2015

^27^ Park J, Yu HT. Recommendations on the sample sizes for multilevel latent class models. *Educational and Psychological Measurement*. 2018; 78(5):737-761. doi:10.1177/0013164417719111

^28^ Tein JY, Coxe S, Cham H. Statistical power to detect the correct number of classes in latent profile analysis. Structural Equation Modeling. 2013;20(4):640-657. doi:10.1080/10705511.2013.824781.

^29^ Ferguson SLG, Moore EW, Hull DM. Finding latent groups in observed data: A primer on latent profile analysis in Mplus for applied researchers. *International Journal of Behavioral Development*. 2020;44(5):458-468. doi:10.1177/0165025419881

^30^ Finch WH, Bronk KC. Conducting confirmatory latent class analysis using Mplus. Structural Equation Modeling. 2011;18:132-151. doi:10.1080/10705511.2011.532732

^31^ Peugh J, Fan X. Enumeration index performance in generalized growth mixture models: A Monte Carlo test of Muthen’s (2003) hypothesis. *Structural Equation Modeling*. 2015;22(1):115-131. doi:10.1080/10705511.2014.919823

^32^ Rovine MJ, von Eye A. Applied computational statistics in longitudinal research. Cambridge, MA: Academic Press; 1991

^33^ Tisak J, Meredith W. Descriptive and associated developmental models. In A. von Eye (Ed.), Statistical methods in longitudinal research (Vol. 2, pp. 387–406). San Diego, CA: Academic Press; 1990

^34^ Tucker LR. Learning theory and multivariate experiment: Illustration of generalized learning curves. In Cattell RB (Ed.), Handbook of multivariate experimental psychology (pp. 476-501). Chicago: Rand McNally; 1966

^35^ Hu LT, Bentler PM. Cutoff criteria for fit indexes in covariance structure analysis: Conventional criteria versus new alternatives. *Structural Equation Modeling*. 1999;6(1):1-55. doi:10.1080/10705519909540118

^36^ Kline RB. Principles and practice of structural equation modeling (4th ed.). Guilford Press; 2016

^37^ Cheong J. Accuracy of estimates and statistical power for testing meditation in latent growth curve modeling. *Struct. Equ. Model.* 2011;18(2):195-211. doi:10.1080/10705511.2011.557334

^38^ Willett JB, Sayer AG. Using covariance structure analysis to detect correlates and predictors of individual change over time. *Psychological Bulletin*. 1994;116(2):363–381. doi:10.1037/0033-2909.116.2.363

^39^ Parsons CE, Crane C, Parsons LJ, Fjorback LO, Kuyken W. Home practice in mindfulness-based cognitive therapy and mindfulness-based stress reduction: A systematic review and meta-analysis of participants’ mindfulness practice and its association with outcomes. *Behaviour Research and Therapy*. 2017;95:29-41. doi:10.1016/j.brat.2017.05.004

^40^ van der Velden AM, Kuyken W, Wattar U, Crane C, Pallesen KJ, Dahlgaard J, Fjorback LO, Piet J. A systematic review of mechanisms of change in mindfulness-based cognitive therapy in the treatment of recurrent major depressive disorder. *Clin Psychol Rev*. 2015;37:26-39. doi:10.1016/j.cpr.2015.02.001

^41^ Tickell A, Byng R, Crane C, Gradinger F, Hayes R, Robson J, Cardy J, Weaver A, Morant N, Kuyken W. Recovery from recurrent depression with mindfulness-based cognitive therapy and antidepressants: a qualitative study with illustrative case studies. *BMJ open*. 2020;10(2):e033892. doi:10.1136/bmjopen-2019-033892

^42^ Kazdin AE, Harris MG, Hwang I, Sampson NA, Stein DJ, Viana MC, Vigo DV, Wu CS, Aguilar-Gaxiola S, Alonso J, Benjet C, Bruffaerts R, Caldas-Almeida JM, Cardoso G, Caselani E, Chardoul S, Cía A, de Jonge P, Gureje O, Haro JM, Karam EG, Kovess-Masfety V, Navarro-Mateu F, Piazza M, Posada-Villa J, Scott KM, Stagnaro JC, Ten Have M, Torres Y, Vladescu C, Kessler RC; WHO World Mental Health Survey collaborators. Patterns, predictors, and patient-reported reasons for antidepressant discontinuation in the WHO World Mental Health Surveys. *Psychol Med*. 2023;54(1):67-78. doi:10.1017/S0033291723002507

^43^ Van Leeuwen E, van Driel ML, Horowitz MA, Kendrick T, Donald M, De Sutter AI, Robertson L, Christiaens T. Approaches for discontinuation versus continuation of long-term antidepressant use for depressive and anxiety disorders in adults. *Cochrane Database Syst Rev*. 2021; 4(4):CD013495. doi:10.1002/14651858.CD013495.pub2

^44^ Kuyken W, Hayes R, Barrett B, Byng R, Dalgleish T, Kessler D, Lewis G, Watkins E, Brejcha C, Cardy J, Causley A, Cowderoy S, Evans A, Gradinger F, Kaur S, Lanham P, Morant N, Richards J, Shah P, Sutton H, Vicary R, Weaver A, Wilks J, Williams M, Taylor RS, Byford S. Effectiveness and cost-effectiveness of mindfulness-based cognitive therapy compared with maintenance antidepressant treatment in the prevention of depressive relapse or recurrence (PREVENT): a randomised controlled trial. Lancet. 2015;386(9988):63-73. doi:10.1016/S0140-6736(14)62222-4

^45^ MacKinnon DP. An introduction to statistical mediation analysis. New York NY: Routledge; 2008

^46^ Hayes AF. An Index and Test of Linear Moderated Mediation. Multivariate behavioral research. 2015;50(1):1–22. doi:10.1080/00273171.2014.962683

^47^ MacKinnon, D. P., Warsi, G., & Dwyer, J. H. (1995). A simulation study of mediated effect measures. Multivariate Behavioral Research, 30, 41–62

^48^ Preacher, K. J., & Kelley, K. (2011). Effect size measures for mediation models: Quantitative strategies for communicating indirect effects. Psy- chological Methods, 16, 93–115.

^49^ Fairchild AJ, Mackinnon DP, Taborga MP, Taylor AB. R2 effect-size measures for mediation analysis. Behav Res Methods. 2009 May;41(2):486-98

^50^ Lachowicz, M. J., Preacher, K. J., & Kelley, K. (2018). A novel measure of effect size for mediation analysis. Psychological methods, 23(2), 244–261

^51^ Cohen, J. (1988). Statistical power analysis for the behavioral sciences. Hillsdale, NJ: Erlbaum.

^52^ Borenstein, M., Hedges, L. V., Higgins, J. P. T., & Rothstein, H. R. (2009). Introduction to Meta-Analysis, Chapter 7: Converting Among Effect Sizes. Chichester, West Sussex, UK: Wiley.
